# Esters with imidazo [1,5-c] quinazoline-3,5-dione ring spectral characterization and quantum-mechanical modeling

**DOI:** 10.1007/s00894-017-3284-1

**Published:** 2017-03-08

**Authors:** K. Hęclik, A. Szyszkowska, D. Trzybiński, K. Woźniak, A. Klasek, I. Zarzyka

**Affiliations:** 1 Department of Chemistry, The University of Technology, Powstańców Warszawy 6, 35-959 Rzeszow, Poland; 2 Department of Chemistry, Biological and Chemical Research Centre, University of Warsaw, Żwirki i Wigury 101, 02-089 Warsaw, Poland; 3 Faculty of Technology, Department of Chemistry, Tomas Bata University in Zlin, CZ-762 72 Zlin, Czech Republic

**Keywords:** Ester, Imidazo[1,5-c]quinazoline ring, Quantum-mechanical modeling, Spectral characterization

## Abstract

1-phenyl-2H,6H-imidazo[1,5-c]quinazoline-3,5-dione reacts with ethyl bromoacetate under mild conditions to give 2-(ethoxycarbonylmethyl)-1-phenyl-6H-imidazo[1,5-c]quinazoline-3,5-dione (MEPIQ) and next 2,6-bis(ethoxycarbonylmethyl)-1-phenylimidazo[1,5-c]quinazoline-3,5-dione (BEPIQ). The products were isolated at high yield and identified on the basis of IR, ^1^H- and ^13^C-NMR, UV spectroscopy, and X-ray crystallography. Diester (BEPIQ) can be presented by 16 possible pair of enantiomers. Only one pair of them is the most stable and crystallizes which is shown crystallographic research. Based on quantum-mechanical modeling, with the use of DFT method, which conformers of mono- and diester and why they were formed was explained. It was calculated that 99.93% of the monoester (MEPIQ) is formed at position No. 2 and one pair of the monoester conformers, from six possible, has the largest share (51.63%). These results afforded to limit the number of diester conformers to eight. Unfortunately, the quantum-mechanical calculations performed that their shares are similar. Further quantum-mechanical modeling showed that conformers are able to undergo mutual transformations. As a result only one pair of diester conformers forms crystals. These conformers have substituents in trans position and these substituents are located parallel to imidazoquinazoline ring. This allows for the denser packing of the molecules in the unit cell.

**Electronic supplementary material:**

The online version of this article (doi:10.1007/s00894-017-3284-1) contains supplementary material, which is available to authorized users.

## Introduction

Compounds containing imidazoquinoline and imidazoquinazoline moieties have been reported to possess interesting properties. Until now, more than 100 imidazo[4,5-c]quinolin-2-one derivatives have been described in the literature, of which at least half show biological activity in various aspects. More recently, due to their biological properties, there has also been great interest in 3,3-disubstituted quinoline-2,4-diones such as, for example, 3,3-diazidoquinoline-2,4-dione which behaes as an inhibitor of the platelet aggregation and 3-hydroxy-3-alkylquinoline-2,4-dion which occurs in the bacteria organisms and it works as an antibiotic [1].

Imidazoquinazolines have been widely applied in pharmacy and medicine due to their versatile biological activities, such as antitumor, antiviral, antibacterial, and anticonvulsant activity. There are drugs containing imidazoquinazolines currently available on the market [2]. One of them is anagrelide, i.e., 6,7-dichloro-1H,5H-imidazo[2,1-b]quinazoline-2(3H)-one, used in the treatment of thrombosis, essential thrombocythemia, or the chronic myelogenous leukemia. Another example is quazinone, i.e., (3R)-6-chloro-3-methyl-5H,10H-imidazo[2,1-b]quinazoline-2(3H)-one, which is widely distributed as a component of heart disease drugs. These types of substances are also derivatives of imidazo[1,5-a]quinazoline such as NNC 14-0185 and 14-0189 NNC, which exhibit an anticonvulsant activity in rats and mice. In the future, they may be used for the production of antiepileptic drugs [2, 3].

Research has shown that compounds containing imidazoquinazoline moiety can be used as the neutralizing agents’ free radicals and thus prevent lipid peroxidation and cell damage. Tested imidazo[1,2-c]quinazoline derivatives were found to be good inhibitors of the lipid peroxidation [4].

Imidazo[1,5-c]quinazoline-3,5-dione derivatives, obtained during reaction 3-aminoquinolinediones with urea as a result of rearrangement [5, 6], can also have biological activity. Unfortunately, they are sparingly soluble and because of that fact their application is difficult. Therefore, there is a need to find more soluble derivatives of these compounds. One of the ways is to obtain their ester derivatives.

This paper illustrates the example of 1-phenyl-2H,6H-imidazo[1,5-c]quinazoline-3,5-dione (PIQ). With the use of this compound, mono- and diester were obtained and their spectral and physical properties characterization was made. In addition, the spatial structure of the obtained esters was explained based on the quantum-mechanical calculations by means of DFT method.

## Methods

### Materials

PIQ was prepared according to literature procedure [7]. The rest of the reagents were purchased and used as received: potassium carbonate, pure for analysis, POCH, Poland; N,N-dimethylformamide (DMF), pure for analysis, Chempur, Poland; ethyl bromoacetate, 98%, Sigma-Aldrich, US; chloroform, pure for analysis, Chempur, Poland; sodium sulfate, anhydrous, ≥ 99.0%, ACS Reagent, US; benzene, pure for analysis, Chempur, Poland.

### Synthetic procedures


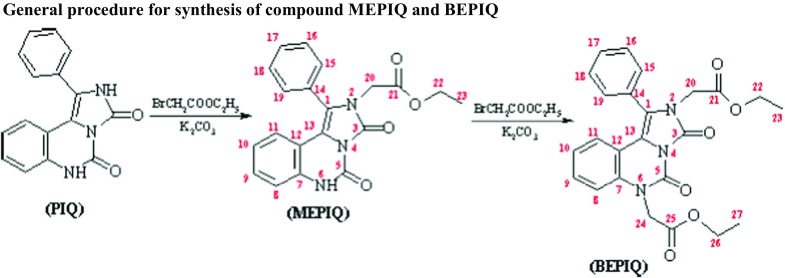


A mixture of PIQ (2.77 g, 10 mmol) and potassium carbonate (3.04 g, 22 mmol) in DMF (40 cm^3^) was stirred at room temperature. After 10 min, ethyl bromoacetate (1.33 cm^3^, 12 mmol or 2.66 cm^3^, 24 mmol) was added. Reaction mixture was protected from moisture with a tube filled with potassium hydroxide and was stirred for 4 h at room temperature and 1 h at temperature 40 °C. After cooling, the reaction mixture was extracted with chloroform. The extract was dried sodium sulfate. After evaporation of chloroform, the crude product was recrystallized from benzene. The course of reaction and also the purity of substances were monitored by TLC (elution systems chloroform-ethanol, 9:1) on Alugram SIL G/UV254 foils (Macherey-Nagel).

2-(ethoxycarbonylmethyl)-1-phenyl-6H-imidazo[1,5-c]quinazoline-3,5-dione (MEPIQ); Yield 70%, light-yellow crystals, mp. 174 °C (benzene), IR: 3570-3320 (s, N−H valence), 3066 (w, CH, deformation of phenyl ring), 2982 (w, CH_3_- and - CH_2_^-^, asymmetric, valence), 2941 (w, CH_3_- and - CH_2_^-^, symmetric, valence), 1762 (s, C = O, valence), 1683, 1613, 1506, 1443 (s, skeletal of phenyl ring), 1639, 1587, 1481 (s, skeletal of quinazoline ring), 1201 (s, C−O(O), symmetric, valence), 1029 (m, C−O(O), asymmetric, valence), 752, 700 (w, C-H in Ph ring, nonplanar deformation), [cm^−1^]; APCI-MS: m/z 365 [M + H]^+^ (100%). APCIMS/MS of precursor ion m/z 365: m/z 337 [M + H-CO]^+^, 319 [M + H-CO-H_2_O]^+^, 294 [M + H–NHCO–CO] ^+^ (100%), 263, 217; EA: Anal. calcd (found) for C_20_H_17_N_3_O_4_: C 69.31 (69.16); H 4.72 (4.69); N 11.56 (11.51); ^1^H-NMR (500 MHz, d_6_-DMSO), δ = 1.10 (3H, t, CH_3_, J_22,23_ = 6.26 Hz), 4.05 (2 H, q, CH_2_^, J^_22,23_^= 6.26 Hz^), 4.22 (2 H, s, CH_2_), 6.72 (1 H, d, J_10,11_ = 8.05 Hz), 6.78 (1 H, t, ^J^_10,9_ = 7.56 Hz), 7.02 (1 H, d, ^J^_9,8_ = 8.05 Hz), 7.14 (1 H, t, ^J^_11,10_ = 7.56 Hz), 7.46 (2 H, m), 7.62 (3 H, m), 10.80 (1 H, s); ^13^C-NMR (d_6_-DMSO), δ = 167.67 (C_21_), 147.85 (C_3_), 144.99 (C_5_), 134.54 (C_13_), 130.76 (C_15_), 130.39 (C_19_), 129.67 (C_17_), 128.48 (C_10_), 129.33 (C_14_), 127.53 (C_16_ and C_18_), 123.18 (C_9_), 121.53 (C_11_), 118.05 (C_1_), 114.86 (C_8_), 113.32 (C_7_), 111.02 (C_12_), 62.33 (C_22_), 43.40 (C_20_), 13.92 (C_23_), [ppm]; UV: 206, 261, 332, [nm].

2,6-bis(ethoxycarbonylmethyl)-1-phenylimidazo[1,5-c]quinazoline-3,5-dione (BEPIQ); Yield 85%, colorless crystals, mp. 182 °C (benzene), IR: 3057 (w, CH, deformation of phenyl ring), 2983 (w, CH_3_- and - CH_2_^-^, asymmetric, valence), 2941 (w, CH_3_- and - CH_2_^-^, symmetric, valence), 1745 (s, C = O, valence), 1681, 1604, 1505, 1444 (s, skeletal of phenyl ring), 1641, 1583, 1486 (s, skeletal of quinazoline ring), 1211 (s, C−O(O), symmetric, valence), 1026 (m, C−O(O), asymmetric, valence), 749, 664 (w, C-H in Ph ring, nonplanar deformation), [cm^−1^]; APCI-MS: m/z 450 [M + H]^+^ (100%); APCIMS/MS of precursor ion m/z 450: m/z 422 [M + H–CO]^+^, 404 [M + H–CO-H_2_O]^+^, 376 [M + H–NHCO–CO] ^+^ (100%), 348, 302; EA: Anal. calcd (found) for C_24_H_23_N_3_O_6_: C 64.11 (64.09); H 5.16 (5.23); N 9.34 (9.14); ^1^H-NMR (500 MHz, d_6_-DMSO), δ = 1.09 (3H, t, CH_3_, J_22,23_ = 6.99 Hz), 1.22 (3H, t, CH_3_, J_26,27_ = 6.99 Hz), 4.05 (2 H, q, CH_2_^, J^_22,23_^= 6.99 Hz^), 4.19 (2H, q, ^−^CH_2_, ^J^_26,27_^= 6.99 Hz^), 4.24 (2 H, s, CH_2_), 4.89 (2 H, s, CH_2_), 6.82 (1 H, d, J_10,11_ = 7.59 Hz), 6.88 (1 H, t, ^J^_10,9_ = 7.59 Hz), 7.12 (1 H, d, ^J^_9,8_ = 8.39 Hz), 7.24 (1 H, t, ^J^_11,10_ = 7.79 Hz), 7.48 (2 H, m), 7.61 (3 H, m), [ppm]; ^13^C-NMR (d_6_-DMSO), δ = 168.20 (C_25_), 167.67 (C_21_), 147.80 (C_3_), 144.99 (C_5_), 134.54 (C_13_), 130.68 (C_15_), 130.34 (C_19_), 129.63 (C_17_), 128.59 (C_10_), 128.22 (C_14_), 127.18 (C_16_ and C_18_), 123.16 (C_9_), 121.48 (C_11_), 117.63 (C_1_), 114.90 (C_8_), 113.43 (C_7_), 112.08 (C_12_), 61.21 (C_26_), 61.15 (C_22_), 43.97 (C_24_), 42.26 (C_20_), 13.96 (C_23_), 13.80 (C_27_), [ppm]; UV: 207, 263, 332, [nm].

### Methods

#### IR spectra

Infrared spectra (4000−400 cm^−1^), as obtained from KBr disks, were recorded on a Bruker ALPHA FT-IR instrument, with a resolution of 0.01 cm^−1^.

#### NMR spectra

NMR spectra were recorded using Bruker 500 MHz spectrometer in deuterated dimethyl sulfoxide (DMSO-d_6_). ^1^H and ^13^C chemical shifts were given on the δ scale (ppm) and were referenced to internal tetramethylsilane (TMS). All two-dimensional (2D) experiments for correlation spectroscopy (^1^H,^1^H-COSY), heteronuclear single-quantum correlation spectroscopy (HSQC), heteronuclear multiple-bond correlation spectroscopy (HMBC) were performed using manufacturer’s software. Proton spectra were assigned using COSY. Protonated carbons were assigned by HSQC. Quaternary carbons were assigned by HMBC.

#### Mass spectra

The positive-ion APCI mass spectra were measured on an ion trap analyzer Esquire 3000 (Bruker Daltonics, Bremen, Germany) within the mass range m/z = 50-1000. Samples were dissolved in chloroform and analyzed by direct infusion at the flow rate of 40 mL/min. The ion source temperature was 300 °C, the APCI probe temperature was 350 °C, the flow rate and the pressure of nitrogen were 3 L/min and 25 psi, respectively. For MS/MS measurements, the collision amplitude was 0.9 V and the isolation width of precursor ions was 4 m/z.

#### UV spectra

Ultraviolet-visible spectroscopy (UV-VIS) measurements were carried out at 25 °C in the range of 200-700 nm with a spectrometer Hewlett Packard 8943 in the methanol solution of a measuring cell with a thickness of 1 cm.

#### Elemental analysis

Elemental analyses (C, H, N) of products were carried out on an elemental analyzer Vario ELIII instrument. It should be noted that all kinds of other atoms were not notified of the test samples even in the residual contents.

#### X-ray crystallography

Single-crystal X-ray diffraction data were collected on a Agilent Technologies SuperNova single source diffractometer with Mo*K*α radiation (*λ* = 0.71073 Å) at 100 (2) K using CrysAlis RED software [8]. The multi-scan empirical absorption correction using spherical harmonics was applied as implemented in SCALE3 ABSPACK scaling algorithms [8]. The structural determination procedure was carried out using the SHELX package [9]. The structure was solved with direct methods and then successive least-square refinements were carried out based on the full-matrix least-squares on *F* ^2^ using the XLMP program [9]. All H-atoms bound to C-atoms were positioned geometrically, with C–H equal to 0.93 Å, 0.96 Å, and 0.97 Å for the aromatic, methyl, and methylene H-atoms, respectively, and constrained to ride on their parent atoms with *U*_iso_ (H) = x*U*_eq_ (C), where x = 1.2 for the aromatic and methylene H-atoms, and x = 1.5 for the methyl H-atoms. The figures for this publication were prepared using ORTEP-3 and Olex2 programs [10, 11].

#### Quantum mechanical calculations

The quantum-mechanical calculations were performed using a set of computational methods based on the electron density of the tested system in the stationary state, i.e., density functional theory (DFT) [12]. The main objective of DFT is to find a value of the functional, which requires an application of successive approximations. Among the proven and widely used functionals, the B3LYP functional was selected (Becke 3-term correlation functional; Lee, Yang, and Parr exchange functional) [13, 14] as the most suitable for the tasks associated with organic compounds. Every functional requires a functional sets, which allows the replacement of differential equations-integral by a system of algebraic equations. In the preliminary studies, the following function bases were taken into account: 6-31G(d,p) [15] 6-311++G(d,p) [16] and aug -CC-pVDZ [17, 18]. They are the most often used function bases in an optimization of the spatial structure of compounds which are structurally similar. Quantum-mechanical calculations were performed by using Gaussian program (version 9) [19].

It should also be mentioned that the computing cluster called HYDRA (model Blade System Actina Solar Hewlett-Packard) working under the control of the operating system Scientific Linux 6 was used for the calculations. The calculated values of Gibbs free energy and other necessary parameters were read using Notepad++ and EDA-Reader applications [20, 21]. Visualizations of the esters structures were performed with the use of Gauss View and Mercury software [22, 23].

## Results and discussion

### Synthesis and spectral characterization of esters with imidazo[1,5-c]quinazoline ring

In the course of our work, we have focused on the subject of the synthesis and characterization of the esters with imidazo[1,5-c]quinazoline ring, i.e., 2-(ethoxycarbonylmethyl)-1-phenyl-6H-imidazo[1,5-c]quinazoline-3,5-dione (MEPIQ) and 2,6-bis(ethoxycarbonylmethyl)-1-phenylimidazo[1,5-c]quinazoline-3,5-dione (BEPIQ).

The products were obtained by simple reaction of PIQ with an equimolar amount or 2-molar excess of ethyl bromoacetate in the presence of a potassium carbonate catalyst. They have been isolated in pure form in good yield 70% (MEPIQ) and 85% (BEPIQ), respectively.

The solubility of both esters is much better than PIQ. These compounds have good solubility (besides DMSO and DMF as PIQ) in benzene, chloroform, acetone, ethyl acetate, and ethyl alcohol.

MEPIQ is formed as the only reaction product of equimolar amounts of the PIQ with ethyl bromoacetate. The reasons for the reaction chemoselectivity during the obtaining of monosubstituted derivatives of PIQ were explained in detail earlier in paper [24].

**Fig. 1:**
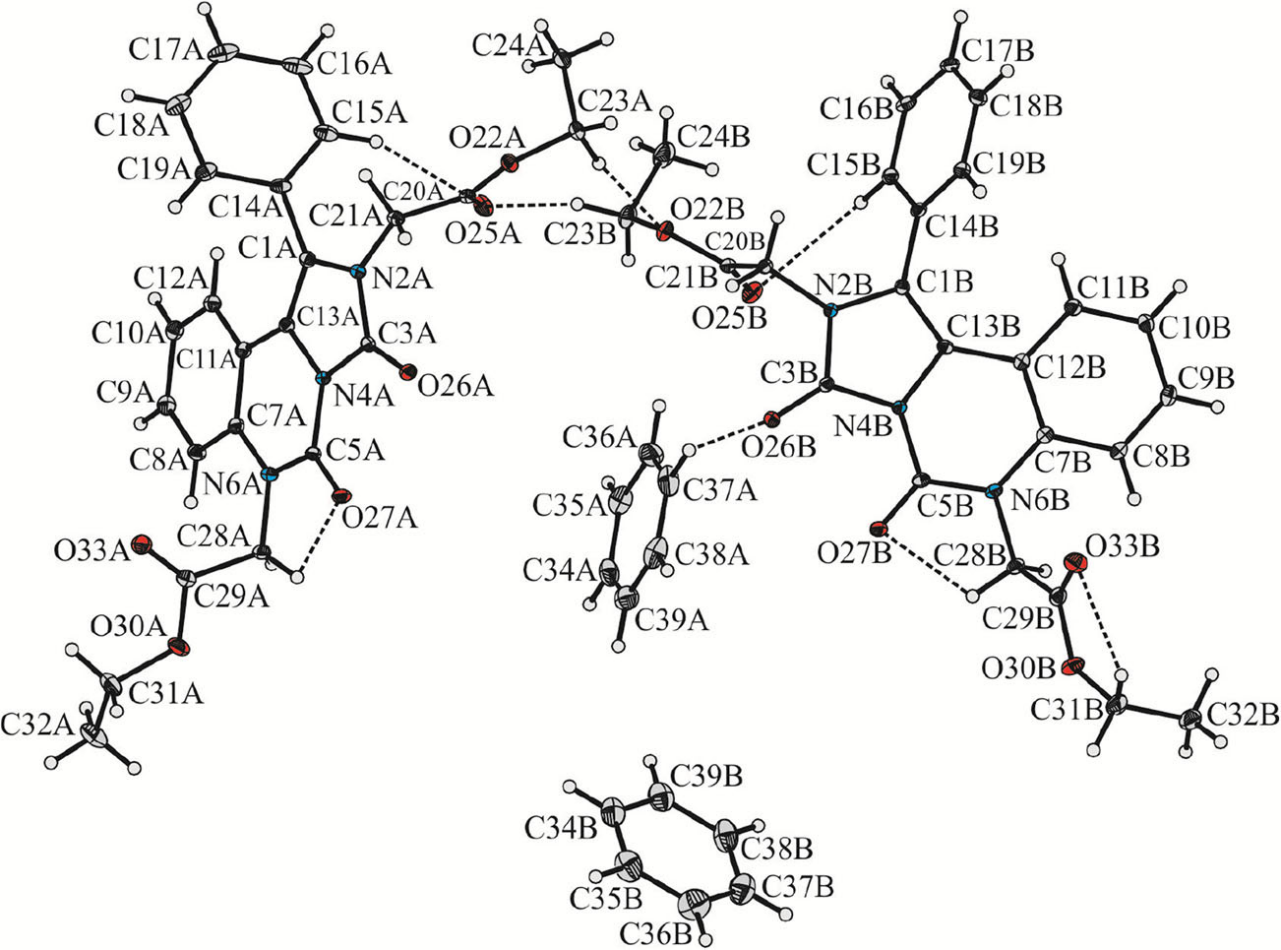
Molecular structure of benzene-solvated BEPIQ with atomic labels. Displacement ellipsoids are drawn at 25% probability level and H-atoms are shown as small spheres of arbitrary radius. The C–H · · · O intermolecular interaction is represented by the dashed line. Selected bond lengths (Å): N2A–C20A 1.451(2); N2B–C20B 1.450(2); N6A–C28A 1.457(2); N6B–C28B 1.452(2); C5A–O27A 1.213(2); C5B–O27B 1.213(2); C3A–O26A 1.222(2); C3B–O26B 1.218(2)

**Fig. 2:**
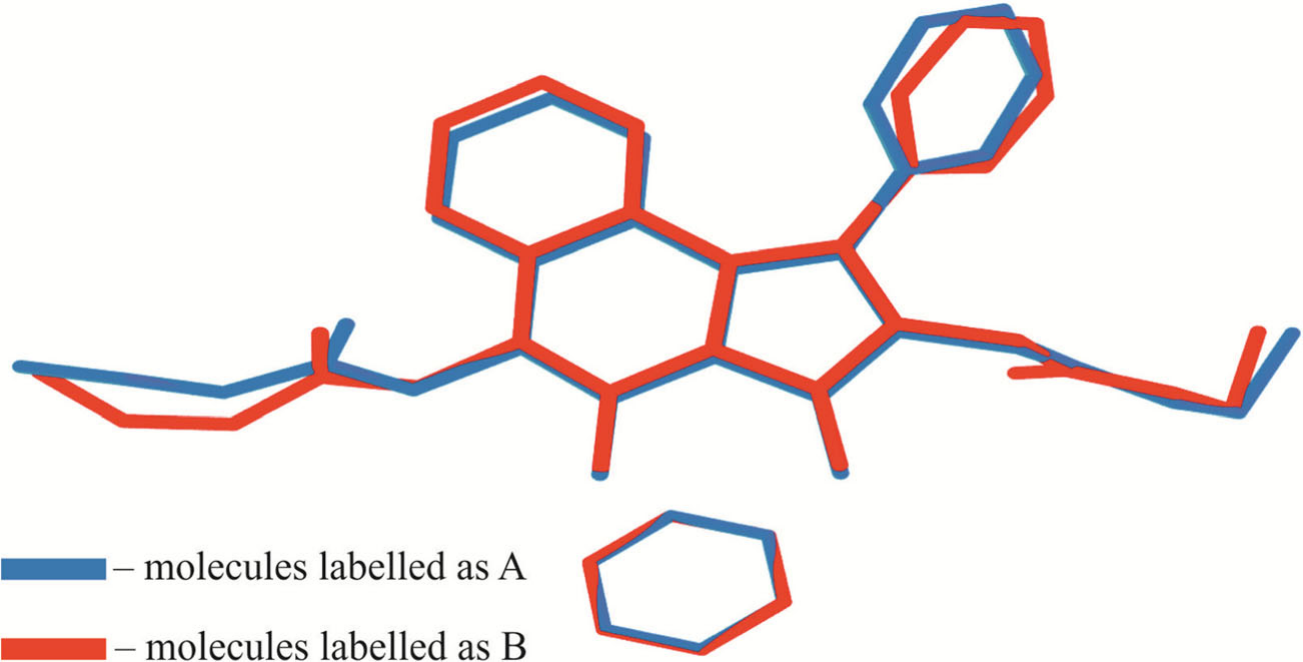
The alignment of benzene-solvated BEPIQ molecules labeled as A and B. Alignment RMSD {N2B, C1B, C13B, …} to {N2A, C1A, C13A, …} with inversion 0.382 Å, while the alignment RMSD {C39B, C38B, C37B, …} to {C39B, C38B, C37B, …} is 0.008 Å

The new esters have been identified by elemental analysis, the ^1^H- and ^13^C-NMR (Fig. 1S, 2S, 4S and 5S), and IR (3S and 8S) and APCIMS/MS measurements (see Experimental part).

All the signals in ^1^H- and ^13^C-NMR spectra of BEPIQ were assigned on the basis of COSY, HSQC, and HMBC experiments to the corresponding atoms. Signals in ^1^H- and ^13^C-NMR spectra of MEPIQ were assigned to the proper atoms based on ^1^H- and ^13^C-NMR spectra of BEPIQ. The signal positions are in accord with the proposed structure MEPIQ and BEPIQ.

A comparison of the elemental analysis results of MEPIQ or BEPIQ and the calculated amount of corresponding elements confirmed the esters compositions.

**Fig. 3:**
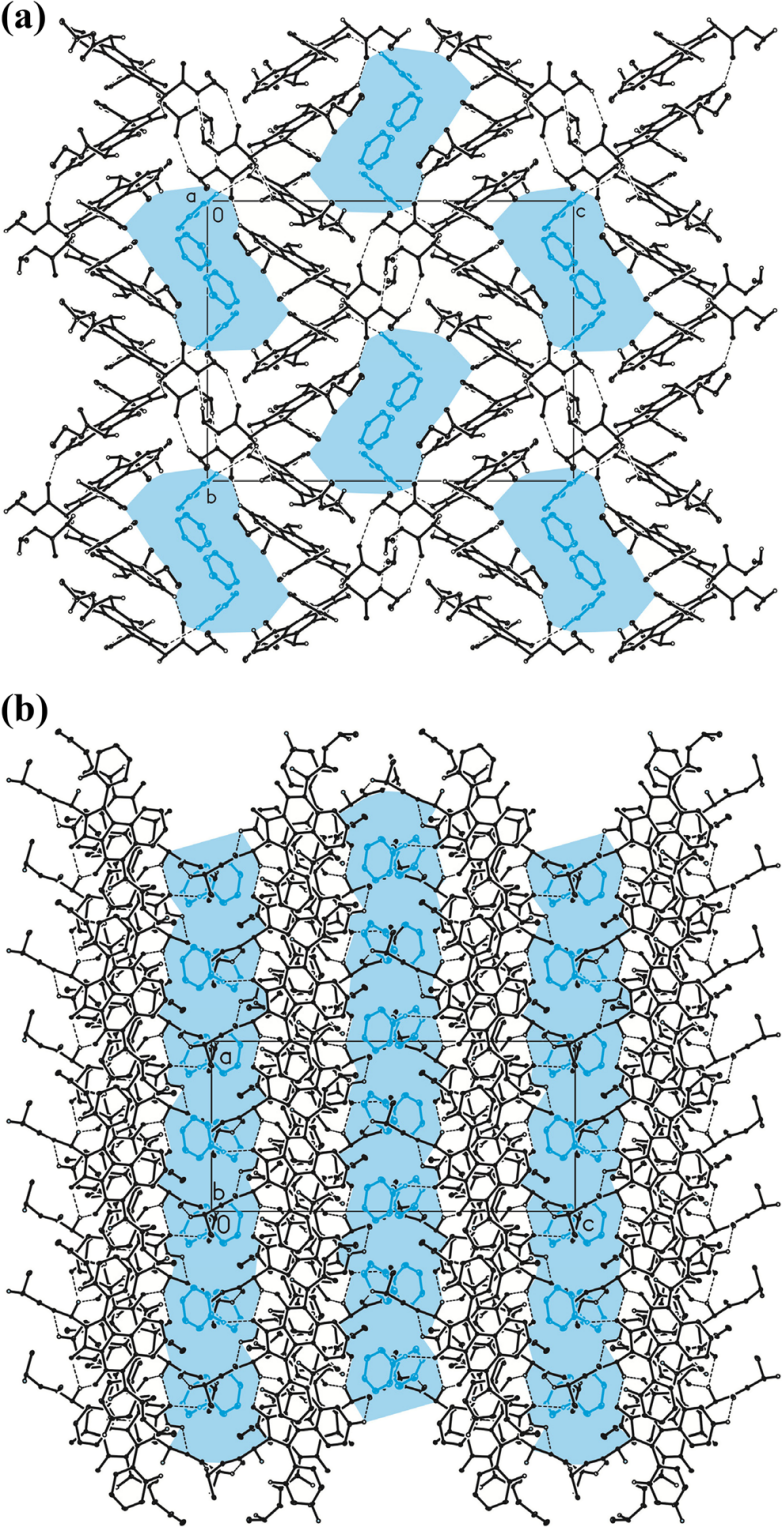
The arrangement of molecules in the crystal lattice of benzene-solvated BEPIQ viewed along *a* **a** and *b*-direction **b**. Hydrogen atoms not involved in H-bond type interactions have been omitted for clarity. 1D-channels filled with solvent molecules have been highlighted in blue. The C–H · · · O hydrogen bonds are represented by the dashed line

In the IR spectra of the new esters (Fig. 3S and 8S), the significant changes are observed in the range of 2000-3500 cm^−1^. The band of the valence vibrations of N-H bonds disappeared partially or totally in the case of MEPIQ and BEPIQ, respectively. The band of the valence vibrations C-H bonds of the aromatic ring is revealed at 3030 cm^−1^. Furthermore, there are visible bands of the symmetrical and asymmetrical valence vibrations of C-H bonds of the methylene and methyl groups at 2890 and 2960 cm^−1^. In the IR spectra (Fig. 3S and 8S), the ν_(C=O)_ stretching vibration bands of MEPIQ (1762 cm^−1^) and BEPIQ (1745 cm^−1^) shift slightly upon substitution of PIQ (1738 cm^−1^) similar to all skeletal bands of imidazoquinazoline and phenyl rings. The ν_(C-O)_ stretching asymmetrical and symmetrical vibration bands of ester (CO)-O bond appear at 1201 (1211) and 1029 (1026) cm^−1^ in the spectrum of MEPIQ (BEPIQ), indicating that substitution at nitrogen atoms takes place.

In the ^13^C-NMR spectrum of MEPIQ (Fig. 2S), the three signals of the sp^3^ hybridized carbon atoms are found at 62.33 (C22), 43.40 (C20), and 13.92 (C23) ppm. In the ^13^C-NMR spectrum of BEPIQ (Fig. 5S), the three additional signals of the sp^3^ carbon atoms are present. Thus, we observe six signals at 61.21 (C26), 61.15 (C22), 43.97 (C24), 42.26 (C20), 13.96 (C23), and 13.80 (C27) ppm. The occurrence in the ^13^C NMR spectra, shift of the carbonyl group at 167.67 (C21) (MEPIQ) or 168.20 (C25) and 167.67 (C21) (BEPIQ), is typical for the ester groups.

Confirmation of the ester structures was obtained by a complete interpretation of NMR spectra. NH protons of PIQ resonating at ca. 11.15 ppm disappear in the ^1^H-NMR spectrum of MEPIQ (Fig. 1S) compared to PIQ spectrum. It confirms the substitution at nitrogen atom No. 2. Proton signals of ethyl group appear as a triplet at 1.10 ppm and a quartet 4.05 ppm. Additionally, a singlet of methylene protons linked to nitrogen atom No. 2. lies at 4.22 ppm.

Proton signals at carbon atoms No. 8, 9, 10, and 11 in the quinazoline ring are clearly separated compared to ^1^H-NMR spectrum of PIQ and appear at 7.12, 6.88, 7.25, and 6.83 ppm, respectively. Similarly, the phenyl ring protons give two distinct signals at 7.46 and 7.62 ppm (see Experimental part).

**Fig. 4:**
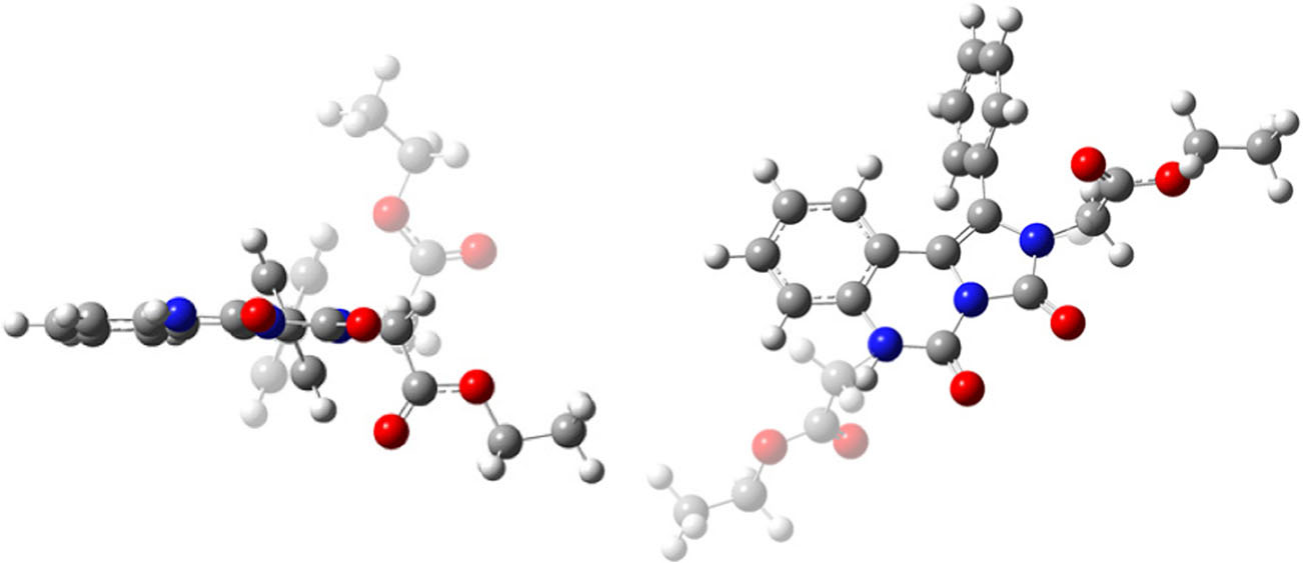
Possible conformers of monoesters PIQ

The ^1^H-NMR spectrum of BEPIQ (Fig. 4S) is quite similar to that of MEPIQ. The signals of both esters differ only slightly, both in the proton and carbon NMR spectra, and have a relative integral ratio of proton signals corresponding to calculated ones from the esters structures. Nevertheless, in ^1^H-NMR spectrum of BEPIQ, proton signal NH at 10.80 ppm is not observed. Moreover, three new signals appear: a triplet at 1.22 ppm (CH_3_), a quartet at 4.19 ppm (O-CH_2_), and a singlet at 4.89 ppm (N-CH_2_), from the ester group substituted at nitrogen atom No. 6.

The positive-ion atmospheric pressure chemical ionization (APCI) mass spectra of both esters studied show the protonated molecules [M + H]^+^ as the only ions in the spectra, which confirms unambiguously the expected molecular weights. The fragment ions observed in MS/MS spectra are in accordance with the suggested structures. The primary cleavage usually leading to the base peak of MS/MS spectrum is the neutral loss of the side chain, as the losses of CO, H_2_O, and others.

### Crystallographic characterization of 2,6-bis(ethoxycarbonylmethyl)-1-phenylimidazo[1,5-c]quinazoline-3,5-dione

Single crystal X-ray diffraction data collection was performed for the BEPIQ compound, crystals of which were obtained by recrystallization of a crude product from benzene. It appears that BEPIQ crystallizes in the orthorhombic *P*2_1_2_1_2_1_ space group in the form of two enantiomers despite of the lack of stereogenic center. The enantiomers are formed in statistical 50%:50% proportions and they are not distinguishable by NMR technique.

Asymmetric unit of the crystal lattice except of two molecules of the investigated compound contains also a pair of solvent molecules (benzene) (Fig. 1).

Table 1 below contains crystallographic data. Selected bond lengths are presented in the caption of Fig. 1 (full list of geometric parameters, including bond lengths, valence and torsion angles can be found in the Supporting information—see Table 1S).

**Table 1:** Crystallographic data and structural refinement details of benzene-solvated BEPIQ

Empirical formula	C_24_H_23_N_3_O_6_, C_6_H_6_
Formula weight	527.56
Temperature (K)	100 (2)
Wavelength (Å)	0.71073
Crystal system	Orthorhombic
Space group	*P*2_1_2_1_2_1_
Unit cell dimensions	
*a* (Å)	11.40934 (10)
*b* (Å)	18.63285 (16)
*c* (Å)	24.2812 (2)
*V* (Å^3^)	5161.90 (8)
Z	8
*D*_*calcd*_ (gcm^−3^)	1.358
Absorption coefficient (mm^−1^)	0.096
Absorption correction type	Multi-scan
*F*_(000)_	2224
Crystal description	colorless plate
Crystal size (mm)	0.297 × 0.195 × 0.075
*Θ* Range for data collection (°)	1.97–26.37
Limiting indices	−14 ≤ *h* ≤ 14
	−23 ≤ *k* ≤ 23
	−30 ≤ *l* ≤ 30
Reflections collected/unique	125676/10561 [*R*_int_ = 0.046]
Completeness of data (%)	99.8
Refinement method	Full-matrix least-squares on *F* ^2^
Data/restraints/parameters	10561/0/707
Goodness-of-fit on *F* ^2^	1.090
Final R indices [*I* >2σ(*I*)]	*R*_1_ = 0.0352; w*R*_2_ = 0.0888
*R* indices (all data)	*R*_1_ = 0.0376; w*R*_2_ = 0.0904
Largest diff. peak and hole (eÅ^−3^)	0.248 and –0.197
CCDC number	1499889

The analysis of molecular overlay (Fig. 2) revealed that the geometry of benzene molecules denoted as A and B is essentially the same. Both BEPIQ molecules have substituents in the trans position and the ethoxycarbonylmethylene groups located parallel to the imidazoquinazoline ring. However, some visible differences in their geometry can be noticed. The respective values of the average deviations from planarity of imidazo[1,5-c]quinazoline-3,5-dione ring system labeled as A and B are 0.089 Å and 0.029 Å, respectively. The phenyl substituent in BEPIQ molecules A and B is inclined to the aforementioned ring system at a dihedral angle of 64.7° and 65.8°. More evident differences occur in orientations of the ethoxycarbonylmethylene substituents. Those changes can be illustrated by specifying the values of dihedral angles between the mean-square quadratic planes defined by the atoms of carboxylic groups and the imidazo [1,5-c] quinazoline-3,5-dione moiety. Thus, the carboxylic groups C21A/O22A/O25A and C21B/O22B/25B are inclined to the specified ring system, respectively, by the angle of 77.2 and 85.7°. The carboxylic groups C29A/O30A/O33A and C29B/O30B/O33B are oriented in such a way that the dihedral angle relative to the imidazo[1,5-c]quinazoline-3,5-dione fragment equals 79.3 and 72.9°.

As mentioned previously, in the case of both BEPIQ conformers, the ester substituents are located parallel to the imidazoquinazoline ring. It allows for a denser packing of the adjacent molecules in the crystal lattice. The search for intermolecular interactions using PLATON [25] has shown that adjacent molecules of BEPIQ are linked by the network of the C–H · · · O, C = O · · · π and π-π intermolecular interactions, which causes the creation of supramolecular framework surrounding 1D-channels filled with the solvent (Fig. 3). The benzene molecules denoted as A are interacting by some weak C–H · · · O intermolecular interactions engaging the O26B carbonyl oxygen atoms of BEPIQ molecules denoted as B (Fig. 1).

### Quantum-mechanical modeling of esters with imidazo[1,5-c]quinazoline-3,5-dione ring

The BEPIQ monocrystalline study has revealed that only one pair of its conformers (Fig. 1) exists, while theoretically there are 32 possible enantiomers (16 pairs) (Fig. 10S). In order to explain this phenomenon, conformers of monoester were analyzed, at the beginning.

Spatial structures of possible conformers of PIQ monoesters substituted at nitrogen atom No. 2 or No. 6 are shown schematically in Fig. 4. The imidazoquinazoline ring is a flat molecule, whereas the phenyl ring is set at a determined angle toward the plane of the imidazoquinazoline ring. The phenyl ring can take two boundary positions. In turn, the ester substituent (ethoxycarbonylmethylene group) for a given substitution reaction can be located above or below the imidazoquinazoline ring plane. In each of these positions, it is located either perpendicular or parallel to the mentioned ring. Therefore, each monoesters substituted at the nitrogen atom No. 2 or No. 6 can theoretically exists in the form of eight conformers (Fig. 11S).

Quantum-mechanical calculations revealed that there are eight stable conformers of monoester substituted at the nitrogen atom No. 6 and only six stable conformers substituted at the nitrogen atom No. 2. Quantum-mechanical calculations were carried out based on the total energy of the conformer as an optimization criterion and on the assumption that there is no interaction between the conformers.

Next, the percentage share of all 14 conformers of MEPIQ was determined taking into account their value of Gibbs free energy (enthalpy) (Table 5S). The results, shown in Table 2, indicate that conformers substituted at nitrogen atom No. 6 almost do not exist. It has been shown that in 99.93% of cases, the formation of the monoester occurs at position No. 2, which was confirmed experimentally.

**Table 2:**
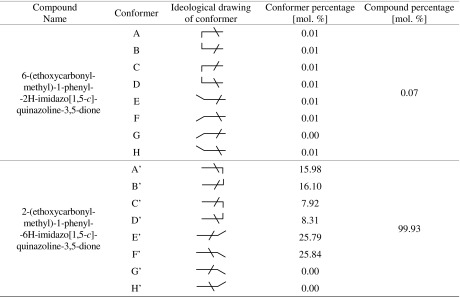
The percentage of N-substituted monoesters of PIQ

Taking into account the most probable conformers of the MEPIQ obtained by substituting at nitrogen atom No. 2, the number of diester conformers decreases to 24 (Fig. 11S).

Among the six conformers of 1-phenyl-2-(ethoxycarbonylmethyl)-2H-imidazo[1,5-c]quinazoline-3,5-dione the one pair of enantiomers (E’ and F’) has the largest share (51.63%). These enantiomers have parallel situated ester substituent to the imidazoquinazoline ring. The rest are the enantiomers with the ethoxycarbonylmethylene substituent located perpendicular to the imidazoquinazoline ring. A parallel arrangement of the substituent is responsible for the spatial discharge (as in the case of both boat and chair conformations of substituted cyclohexane derivatives [26]) and lower energy of conformers, and thus a larger participation in the total population.

**Fig. 5:**
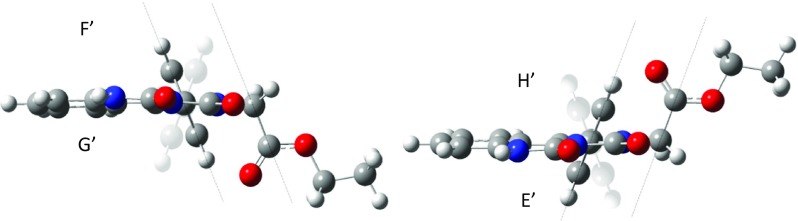
Suggested conformers monoester MEPIQ substituted at the nitrogen atom No. 2 (G’, F’, H’, E’)

Figure 5 shows the four proposed conformers for monoester substituted at the nitrogen atom No. 2 (G’, F’, H’, E’). These prevailing conformers (E’ and F’) are shown in full colors.

**Fig. 6:**
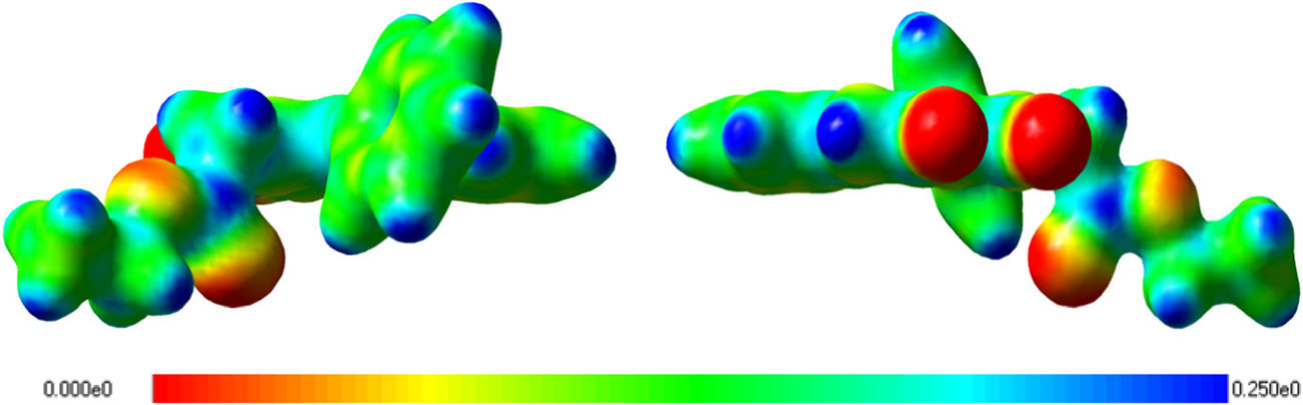
Electrostatic potential of conformer F’

In both conformer E’ and F’, the plane of the phenyl ring is parallel to the plane of the C-C bond (a methylene group–a carbonyl carbon). Therefore, the distance between the carbonyl group and the phenyl ring is the shortest, and the distance between the oxygen atom of the carbonyl group and the nearest hydrogen atom of the phenyl ring is observed to be 2.46 Å [27, 28]. This distance enables an electrostatic interaction between the phenyl ring and the carbonyl group — a high electron density on the oxygen atom of the carbonyl group and a low electron density at the edge of the phenyl ring (Fig. 6). Thus, the conformer becomes more stable. In turn, both longer distances (∼4 Å) and weaker interactions are observed for the G’ and H’ conformers.

Therefore, due to the electrostatic interactions, the ring is “pulled” toward the carbonyl group by the charge existing on the oxygen atom, and the more stable conformer is formed.

**Fig. 7:**
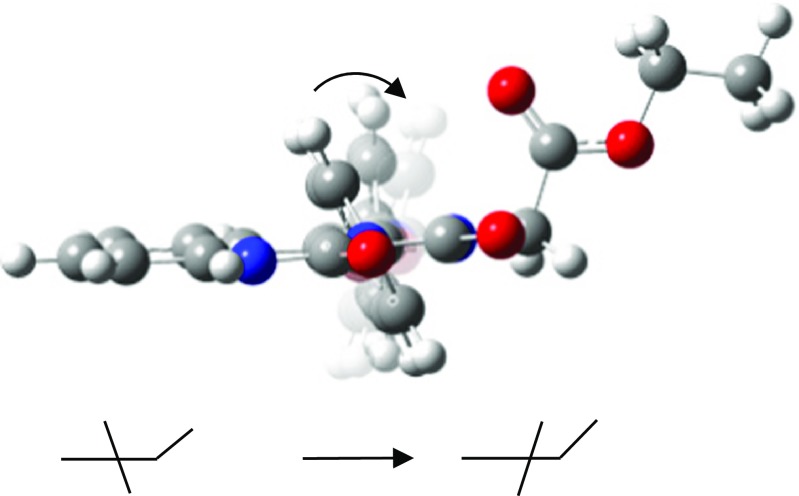
The comp image scheme of transformation conformer H’ into conformer E’

This electrostatic interaction is responsible for the conformational transition of the H’ conformer into the E’ and the G’ conformer into the F’ as a result of changing the angle of inclination of the phenyl ring plane (Fig. 7). This explains a reduction in the number of stable conformers of 1-phenyl-2-(ethoxycarbonylmethyl)-2H-imidazo [1,5*-c*] quinazoline-3,5-dione from eight to six, mentioned earlier.

**Fig. 8:**
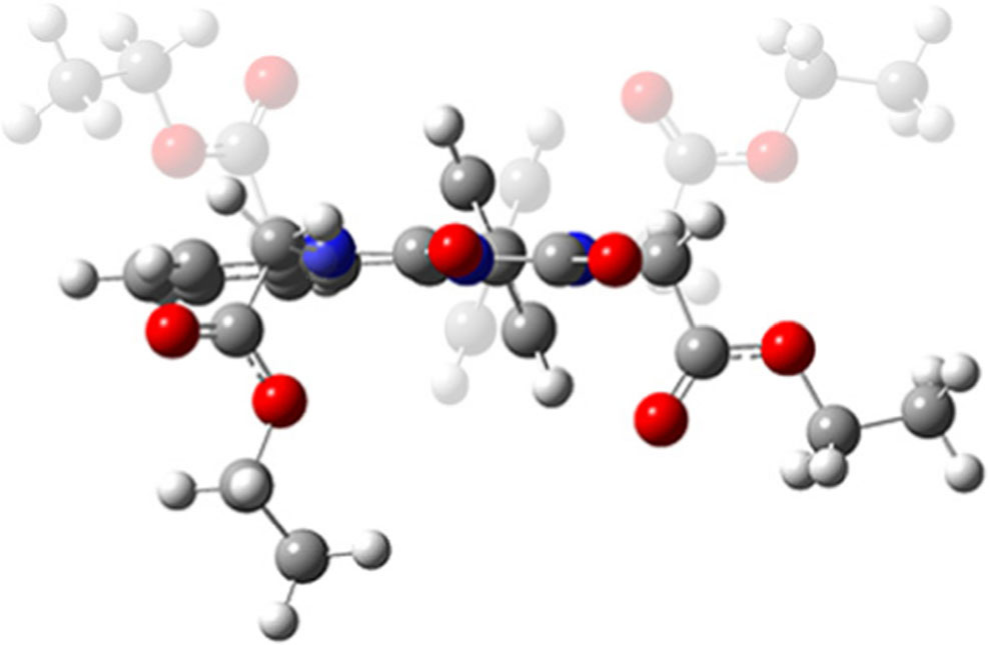
Possible conformers of 1-phenyl-2,6-bis(ethoxycarbonylmehtyl)imidazo[1,5-c]quinazoline-3,5-dione, drawn schematically

Taking into account the predominant conformers E’ and F’ of MEPIQ, four pairs of BEPIQ conformers were determined (Fig. 8). Therefore, eight conformers of diesters have been subjected to quantum-mechanical modeling in order to clarify the reason for the formation of only one conformer in the form of two enantiomers G-E’ and H-F’ (Fig. 1).

As a result of the calculation, the values of Gibbs free energy were provided and then used to determine the shares of the various conformers, i.e., four pairs of enantiomers in the total population. The shares are as shown in Table 2. The content of the enantiomer pair (H-F’ and G-E’) found by crystallography method equals 21.09 mol% (Table 3).

**Table 3:**
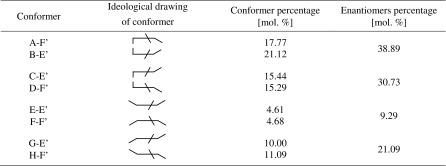
The percentage of 2,6-bis(ethoxycarbonylmehtyl)-1-phenylimidazo[1,5-c]quinazoline-3,5-dione conformers

**Fig. 9:**
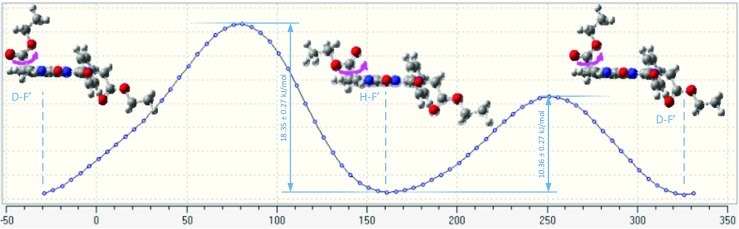
Energy torsional strain in function of the rotation angle about the C-C bond (carbonyl group–methylene group) in the molecule in 2,6-bis(ethoxycarbonyomehtyl)-1-phenylimidazo[1,5-c]quinazoline-3,5-dione

In further investigations, the possibility of mutual transformations of diester conformers was considered. Therefore, the simulation of the total energy value changes of the selected conformers was performed. At first, an analysis of the transformation of D-F’ into H-F’ (one ester substituent is below and the second one is above the imidazoquinazoline ring plane, i.e., they are in trans position) by rotation around the C-C bond (bond between a carbonyl group–a methylene group) was performed (Fig. 9). As a result of the rotation, the substituent which was perpendicular to imidazoquinazoline ring plane becomes parallel and now both substituents of H-F’ take parallel position. It was noted that the energy barrier of the free rotation depends on the rotation direction. A rotation out of the plane of the imidazoquinazoline ring involves energy about 10 kJ mol^−1^. In turn, a rotation above the plane of the imidazoquinazoline ring requires energy about 18 kJ mol^−1^. These values indicate that free rotation is slightly limited but it is possible. For comparison, the difference in total energy values of the terminal ethane conformers’ amounts to 12 kJ mol^−1^ and a rate of the conformation change is 10^6^/s at room temperature [29].

**Fig. 10:**
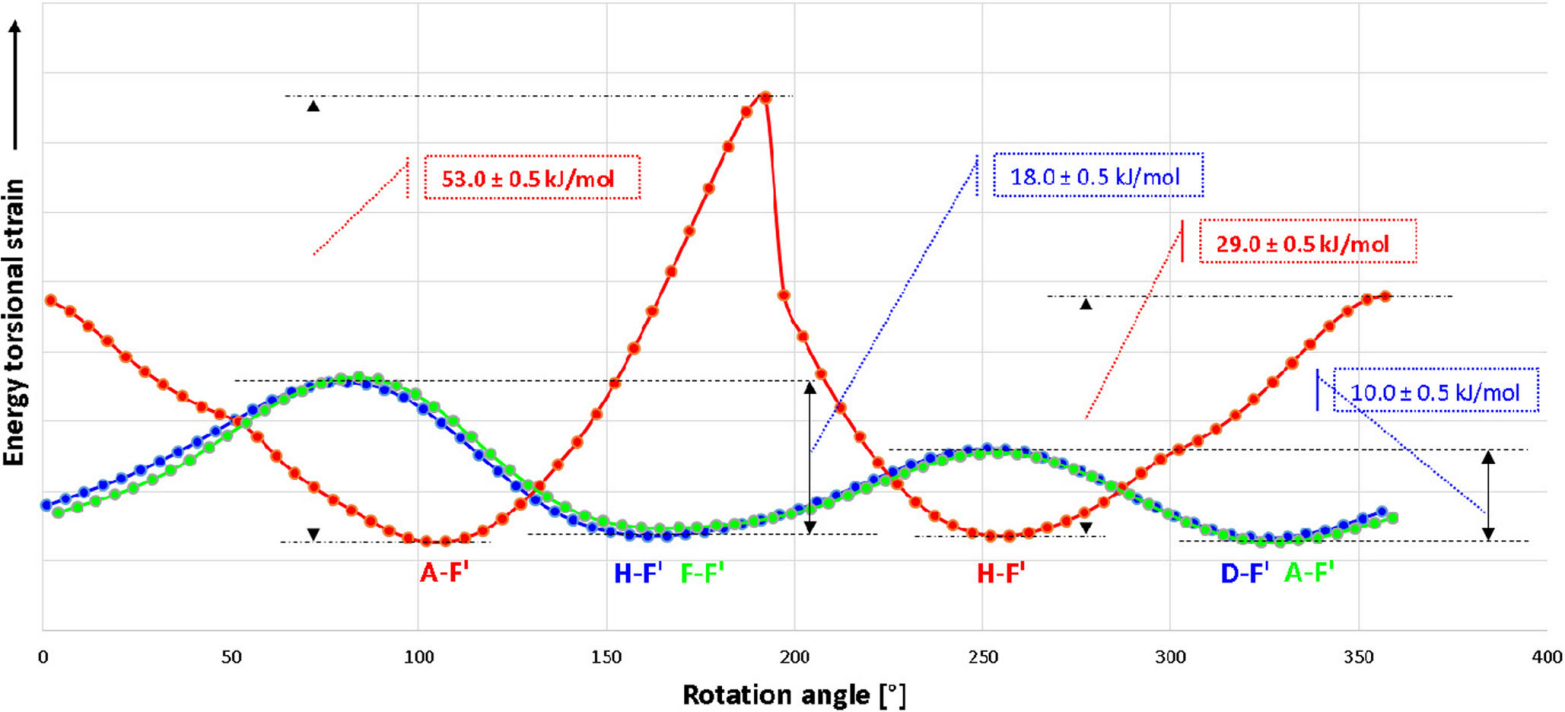
Energy torsional strain in function of the rotation angle about the C-C bond (carbonyl group - methylene group) in the molecule in 2,6-bis(ethoxycarbonyomehtyl)-1-phenylimidazo[1,5-c]quinazoline-3,5-dione

As shown in Fig. 10, an analogous situation refers to transformations of the A-F’ isomer into the F-F’ (both substituent lie at the same side of the imidazoquinazoline ring plane; they are in cis position). The ester substituent also changes its position from the perpendicular to the parallel.

Furthermore, the transformation of conformers B-E’ (cis conformer) into G-E’ (trans conformer), when the configuration change takes palce was considered. Then, the ester substituent changes its location form one side of the imidazoquinazoline ring plane to the second one, and its position changes from the parallel to the perpendicular. It was calculated that the energetic barrier of configuration change (cis into trans) B-E’ in G-E’, amounts to 29 kJ mol^−1^ (or 53 kJ mol^−1^ in the case of conversion from the steric hindrance). It should be noted that the energetic barrier of cyclohexane ring inversion is 46.2 kJ mol^−1^ at the rate of 10^5^/s at room temperature [30].

The obtained resuts indicate the possibility of spontaneous reconfiguration of all diester conformers.

**Fig. 11:**
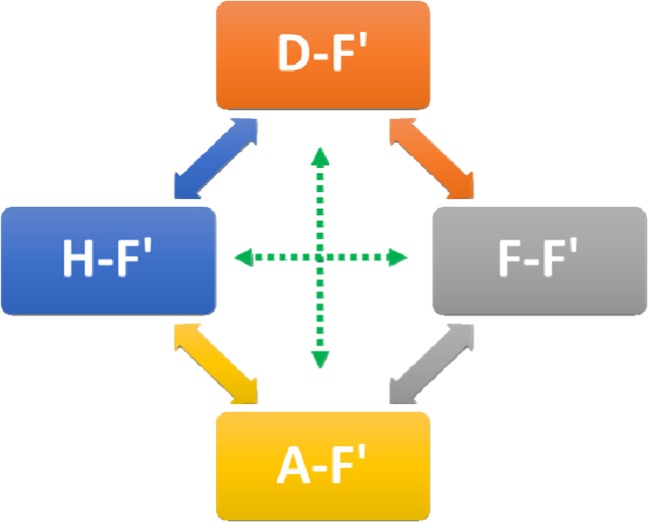
Diagram of the possible conformer transformations

Therefore, it can be thought that during the process of crystal forming, a free transformation occurs according to the scheme shown in Fig. 11.

**Fig. 12:**
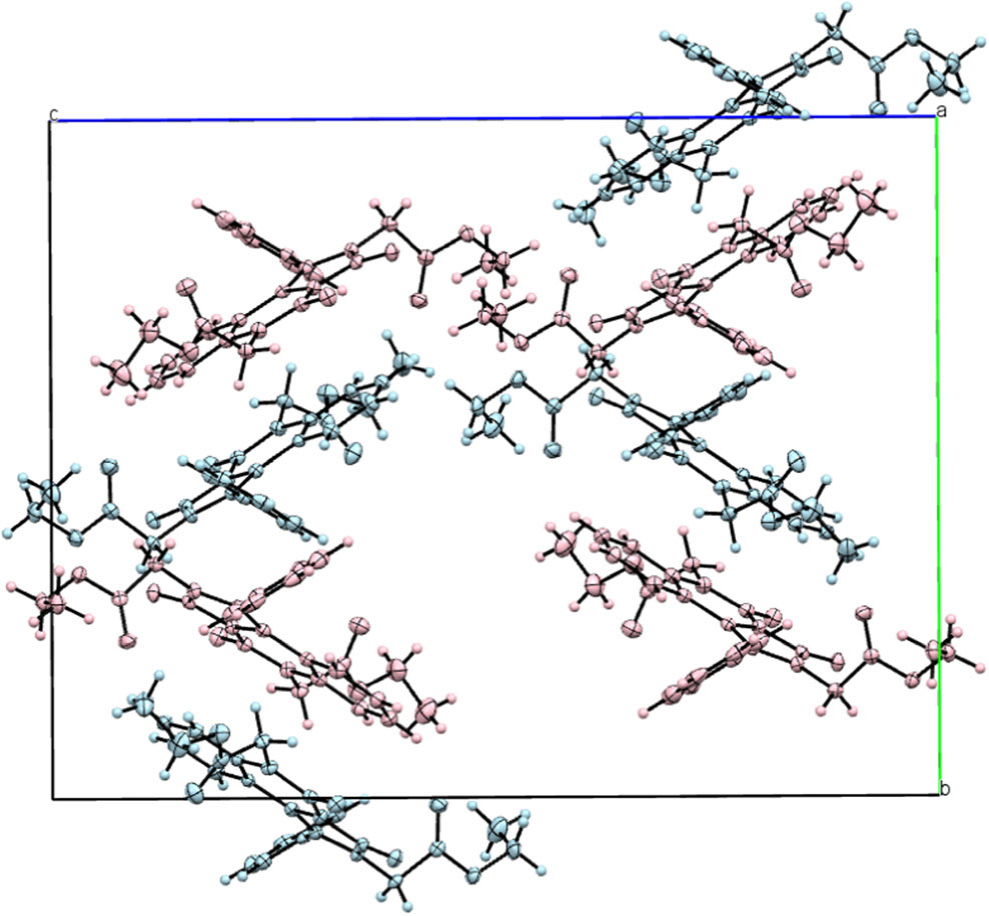
Unit cell of crystallographic network of 1-phenyl-2,6-bis(ethoxycarbonylmehtyl)imidazo[1,5-c]quinazoline-3,5-dione

Finally, only the H-F’ conformer and its mirror image (G-E’) form crystals, as is demonstrated by crystallographic studies (Fig. 1). These conformers have substituents in trans positions and the substituents lie parallel to imidazoquinazoline ring plane. It allows for the denser packing of the molecules in the unit cell. As X-ray diffraction analysis shows (Fig. 12), there are four pairs of enantiomers in the unit cell and, moreover, their imidazoquinazoline rings are parallel to each other. The detailed explanation of the diester conformer packing will be the subject of the next paper.

### Crystallographic structure versus quantum-mechanical model

Comparing the bond lengths from the energetically optimized model from Gaussian with the values measured in crystal (Table 24S), one can see that differences are mostly not bigger than 2%. Merely exceptions are lengths of all bonds between hydrogen and carbon atoms (values of differences amounts from 12 to 17%) and lengths of two bonds hydrogen-oxygen atoms in one of the enantiomers, where deviations reach 11%.

So significant values of differences could be caused by the kind of basis set we used: 6-31++G(d,p). Changing the accuracy of the used data base (e.g., 6-31G(d,p) or 6-311++G(d,p)), did not improve the values.

Differences in angles between bonds (Table 25S) do not exceed 3%, however, maximum value of difference is 3° and it is 111° for O22A-C23A-C24A angle. It is related directly to differences for dihedral angles (Table 26S) of ethoxycarbonylmethyl groups, where relatively large deviations are observed in: C24A—C23A—O22A—C21A, C32A—C31A—O30A—C29A, C32B—C31B—O30B—C29B, and C24B—C23B—O22B—C21B. As we can see, those deviations are related to positions of ethoxycarbonylmethyl groups in crystals relative to simulated model, and probably caused by getting stuck in local minimum by optimization algorithm.

## Conclusions

This work provides the synthesis and spectral characterization of two new compounds — mono- and diesters with the imidazoquinazoline ring.

Diester structure was investigated by single-crystal X-ray diffraction. Crystallographic studies have shown that only one pair of 2,6-bis(ethoxycarbonylmehtyl)-1-phenylimidazo[1,5-c]quinazoline-3,5-dione conformers exists among 16 theoretically possible pairs.

Quantum-mechanical modeling of mono- and diesters explains why only one pair of diester enantiomers is formed in a crystalline structure. The modeling results clearly indicate that the diester conformers are capable of mutual transformations. The energy barriers of the conformer changes are not very high and afford the conformer transformations. Therefore, finally, only one pair of 2,6-bis(ethoxycarbonylmehtyl)-1-phenylimidazo[1,5-c]quinazoline-3,5-dione enantiomers forms crystals. The ester substituents of these conformers have beneficial parallel position toward the imidazoquinazoline ring plane. The imidazoquinazoline rings of these conformers are located parallel to each other in the cell unit for the improved packaging of molecules.

## Electronic supplementary material

Below is the link to the electronic supplementary material.

ESM 1 (DOC 5084 kb)
