# Supplementary material for: Esters with imidazo [1,5-c] quinazoline-3,5-dione ring spectral characterization and quantum-mechanical modeling
Source: J Mol Model. 2017 Mar 8;23(4):107. doi: 10.1007/s00894-017-3284-1 (PMC5343086; doi:10.1007/s00894-017-3284-1)
Supplement: Supplementary file 1 — (DOC 5084 kb) [file 894_2017_3284_MOESM1_ESM.doc]

Esters with imidazo[1,5-c]quinazoline-3,5-dione ring spectral characterization and quantum-mechanical modelling

K. Hęclik*^a^*, A. Szyszkowska*^a^*, D. Trzybiński*^b^*, K. Woźniak*^b^*, A. Klasek*^c^*, I. Zarzyka*^a*^*

*^a.^Department of Chemistry, The University of Technology, Powstańców Warszawy 6, 35-959 Rzeszow, Poland.*

*^b.^Department of Chemistry, Biological and Chemical Research Centre, University of Warsaw, Żwirki i Wigury 101, 02-089, Warsaw, Poland.*

*^c.^Department of Chemistry, Faculty of Technology, Tomas Bata University, CZ-762 72 Zlin, Czech Republic.*


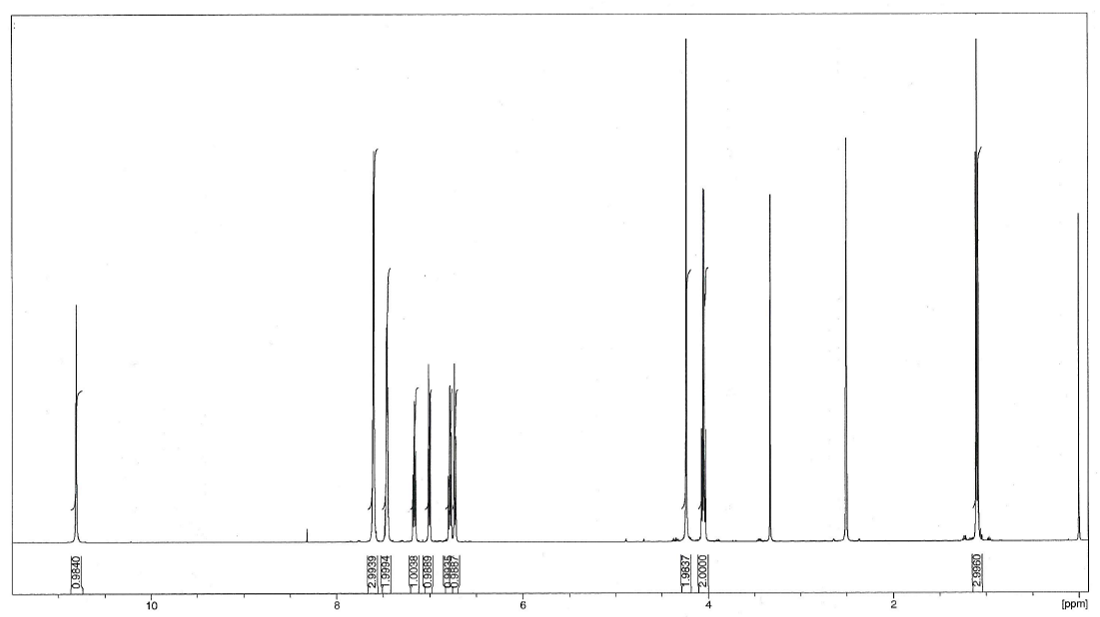


**Fig. 1S.** ^1^H-NMR spectrum of 2-(etoxycarbonylmethyl)-1-phenyl-6H-imidazo[1,5-*c*]quinazo-line-3,5-dione.


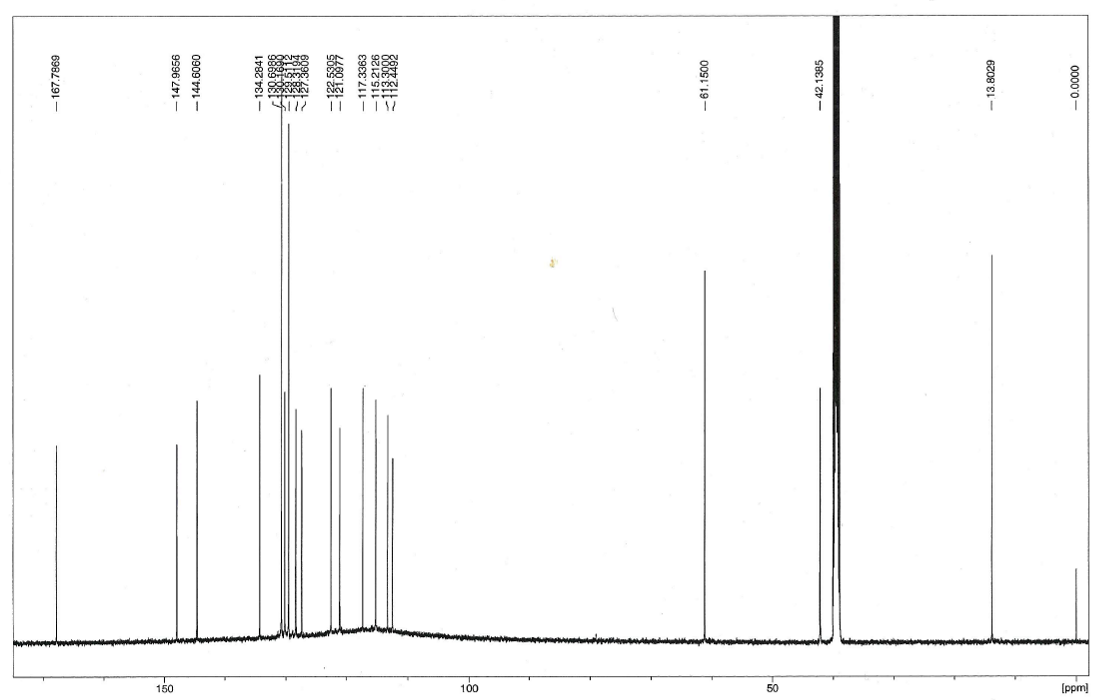


**Fig. 2S.** ^13^C-NMR spectrum of 2-(etoxycarbonylmethyl)-1-phenyl-6H-imidazo[1,5-*c*]quinazo-line-3,5-dione.

**Fig. 3S.** IR spectrum of 2-(etoxycarbonylmethyl)-1-phenyl-6H-imidazo[1,5-*c*]quinazoline-3,5-dione.


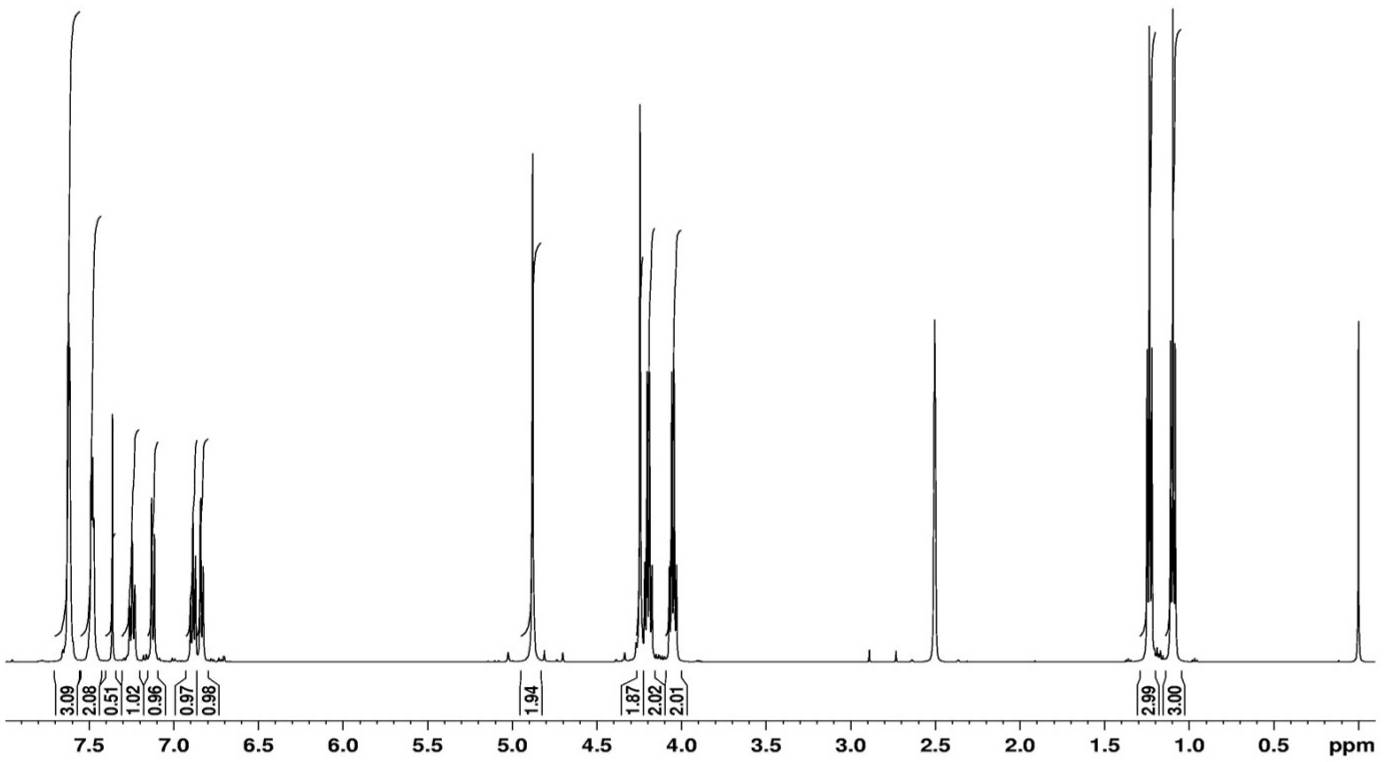


**Fig. 4S.** ^1^H-NMR spectrum of 2,6-bis(etoxycarbonylmethyl)-1-phenylimidazo[1,5-*c*]quinazo-line-3,5-dione.


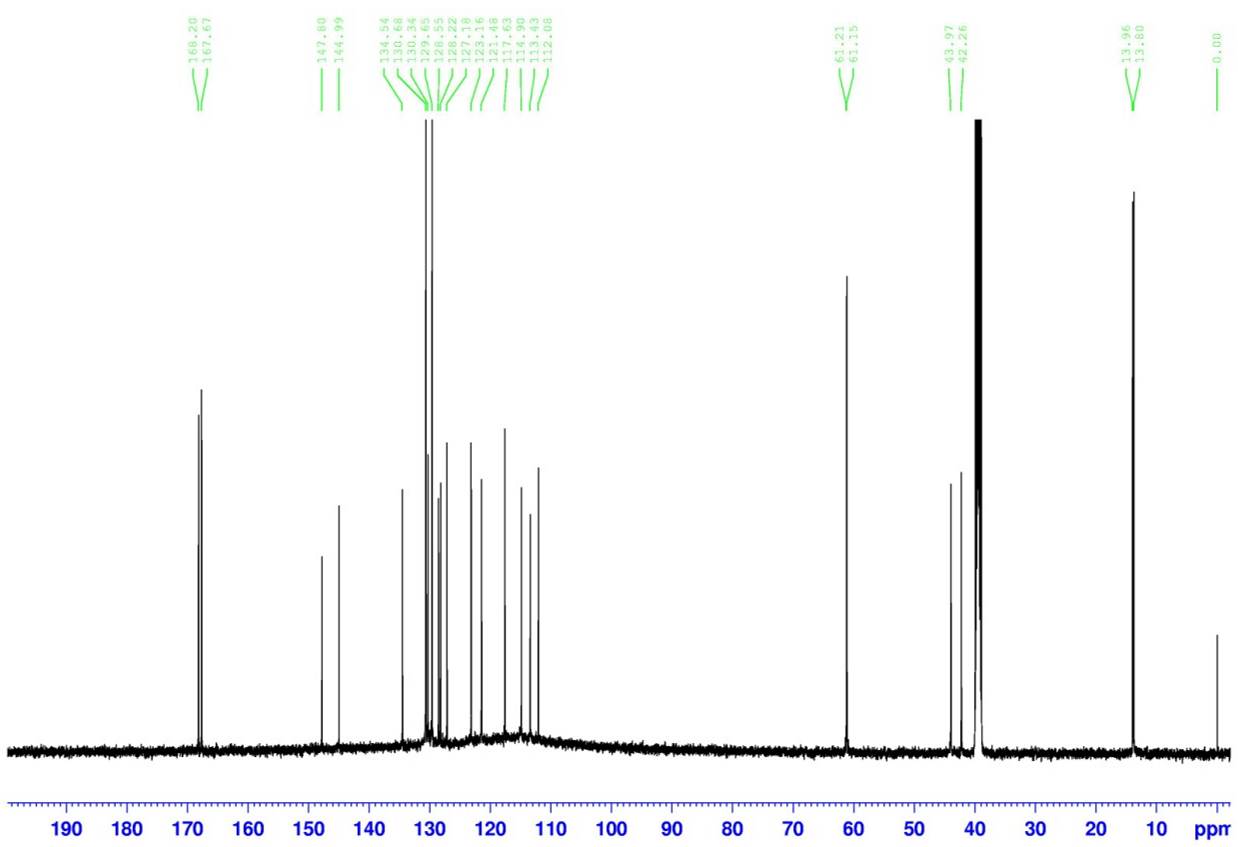
**Fig. 5S.** ^13^C-NMR spectrum of 2,6-bis(etoxycarbonylmethyl)-1-phenylimidazo[1,5-*c*]quinazo-line-3,5-dione.

**
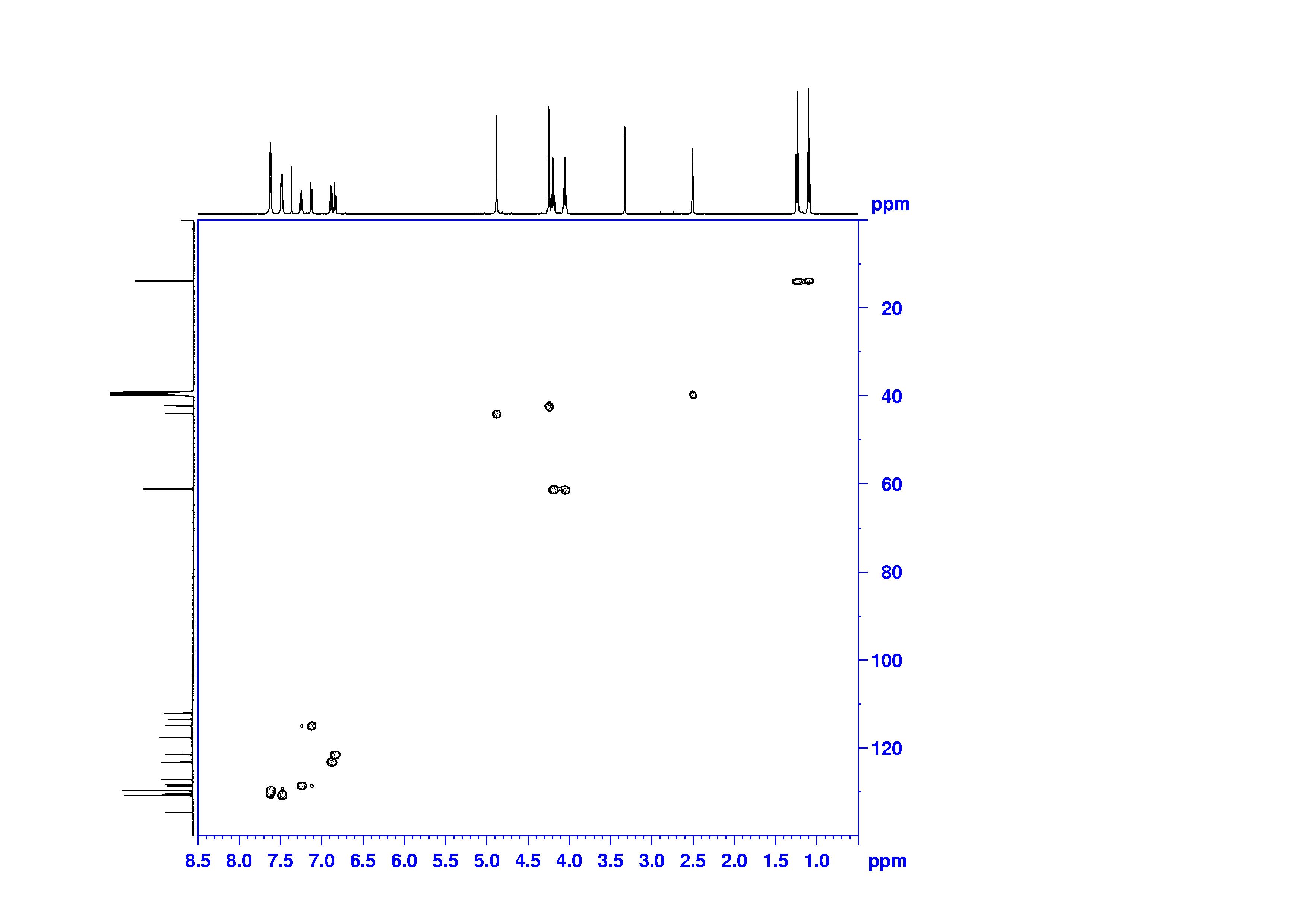
**

**Fig. 6S.** HSQC spectrum of 2,6-bis(etoxycarbonylmethyl)-1-phenylimidazo[1,5-*c*]quinazo-line-3,5-dione.


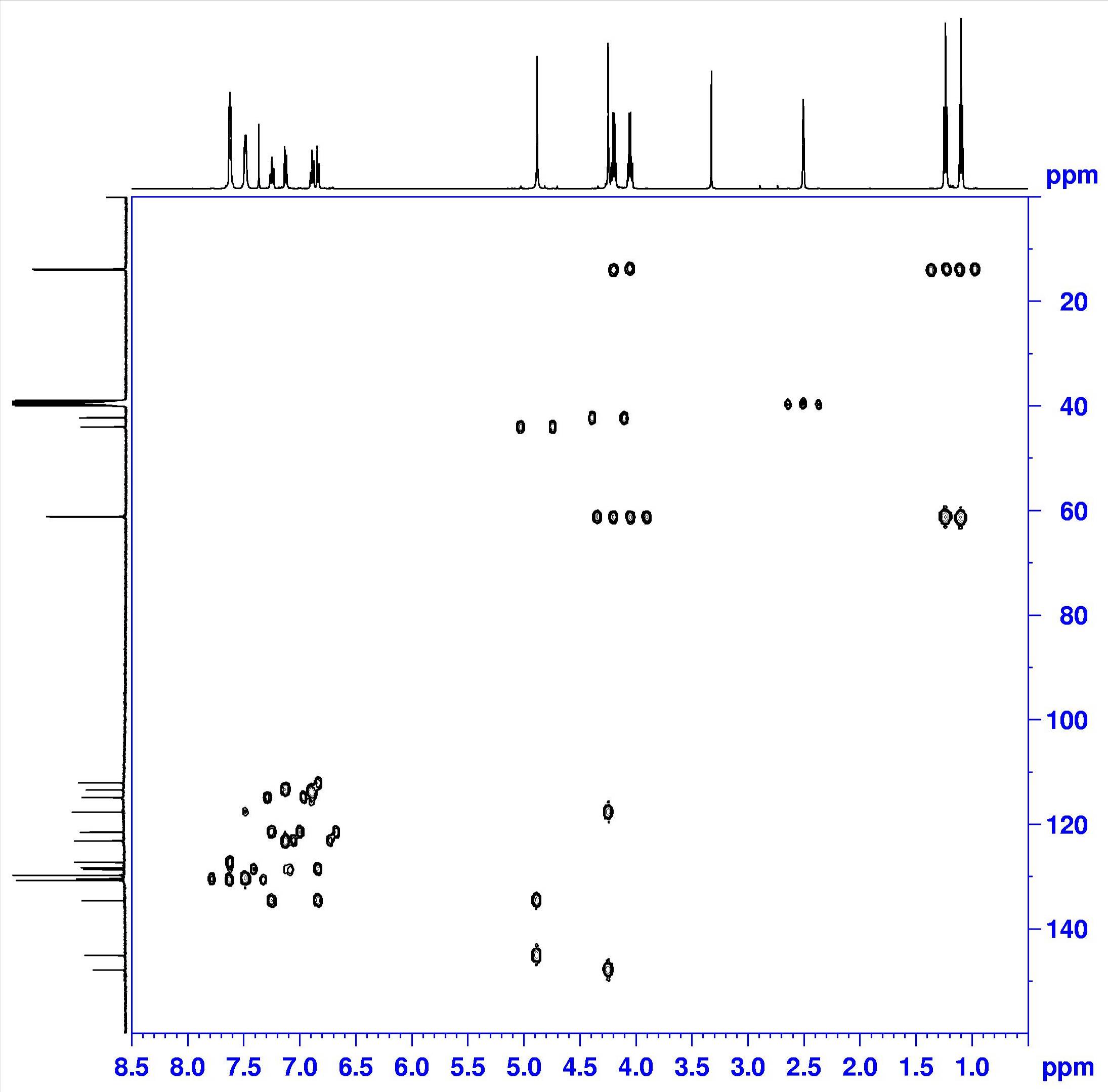


**Fig. 7S.** HMBC spectrum of 2,6-bis(etoxycarbonylmethyl)-1-phenylimidazo[1,5-*c*]quinazo-line-3,5-dione.


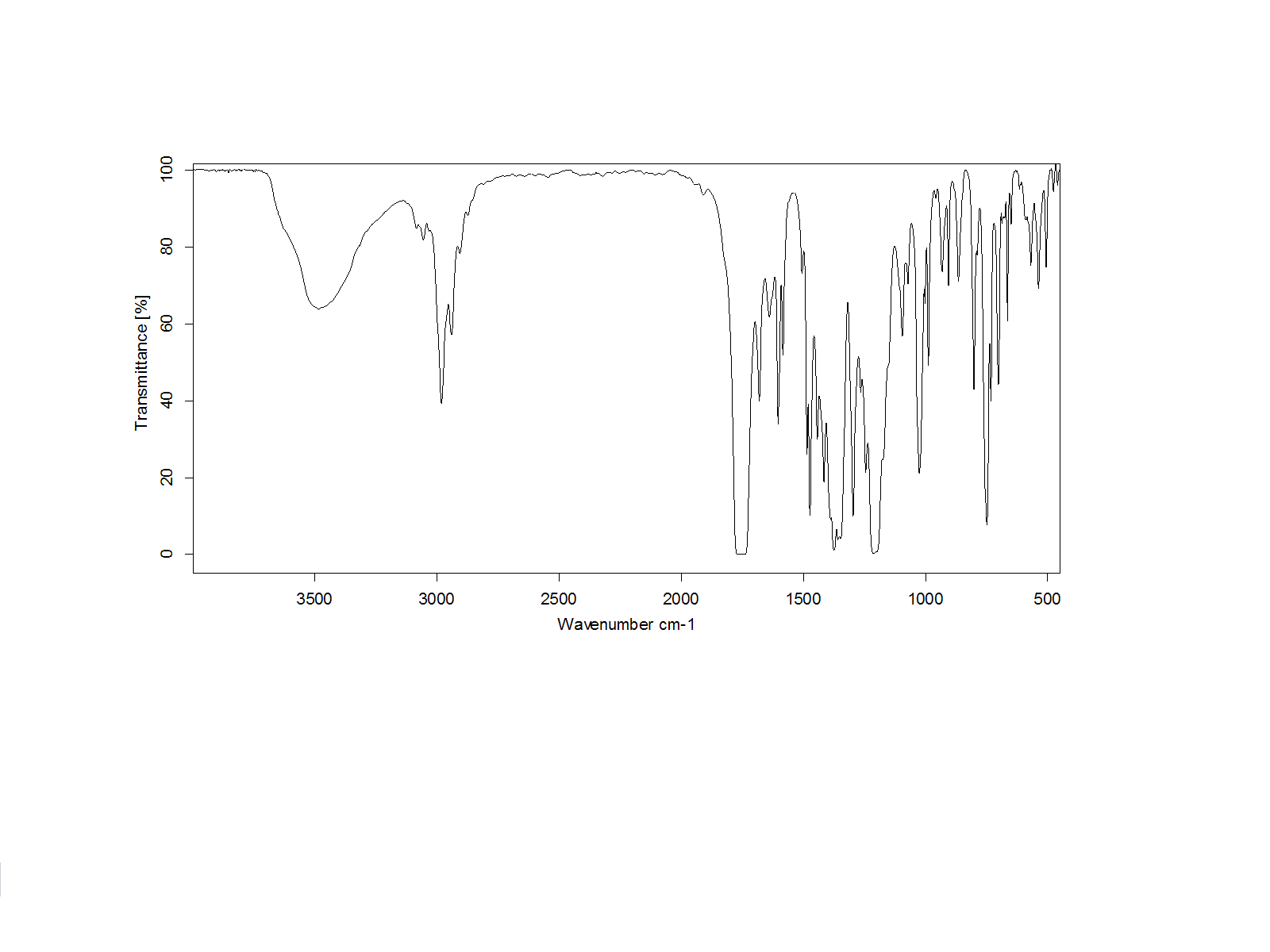


**Fig. 8S.** IR spectrum of 2,6-bis(etoxycarbonylmethyl)-1-phenylimidazo[1,5-*c*]quinazoline-3,5-dione.


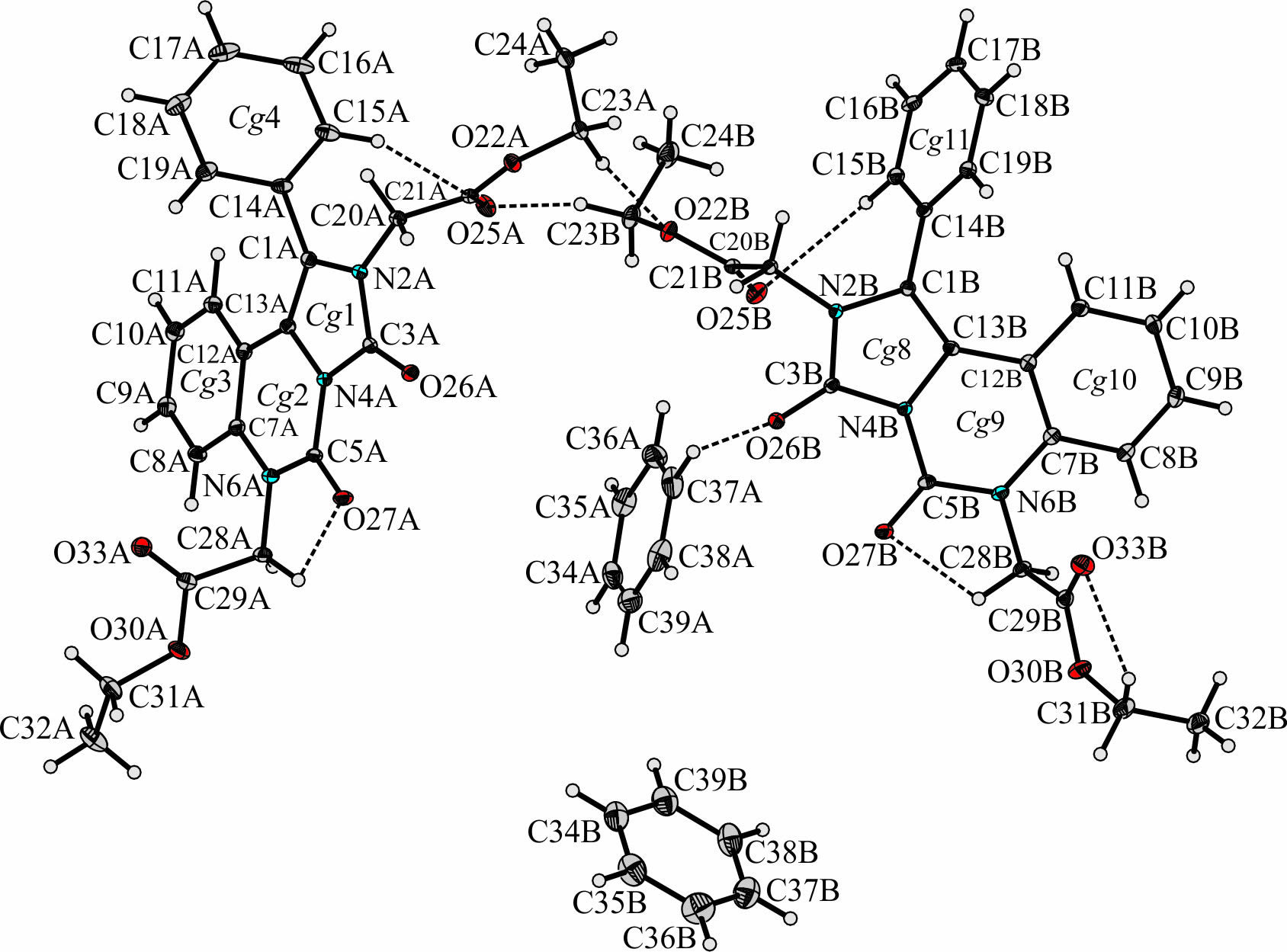


**Fig. 9S.** Molecular structure of benzene-solvated BEPIQ with crystallographic numbering. Displacement ellipsoids are drawn at 25% probability level and H-atoms are shown as small spheres of arbitrary radius. The C–H···O intermolecular interaction is represented by the dashed line. The *Cg*1, *Cg*2, *Cg*3, *Cg*4, *Cg*8, *Cg*9, *Cg*10 and *Cg*11 denote the geometric centers of gravity of the rings delineated by the C1A–N4A/C13A, N4A–C7A/C12A/C13A, C7A–C12A, C14A–C19A, C1B–N4B/C13B, N4B–C7B/C12B/C13B, C7B–C12B, C14B–C19B atoms, respectively.

**
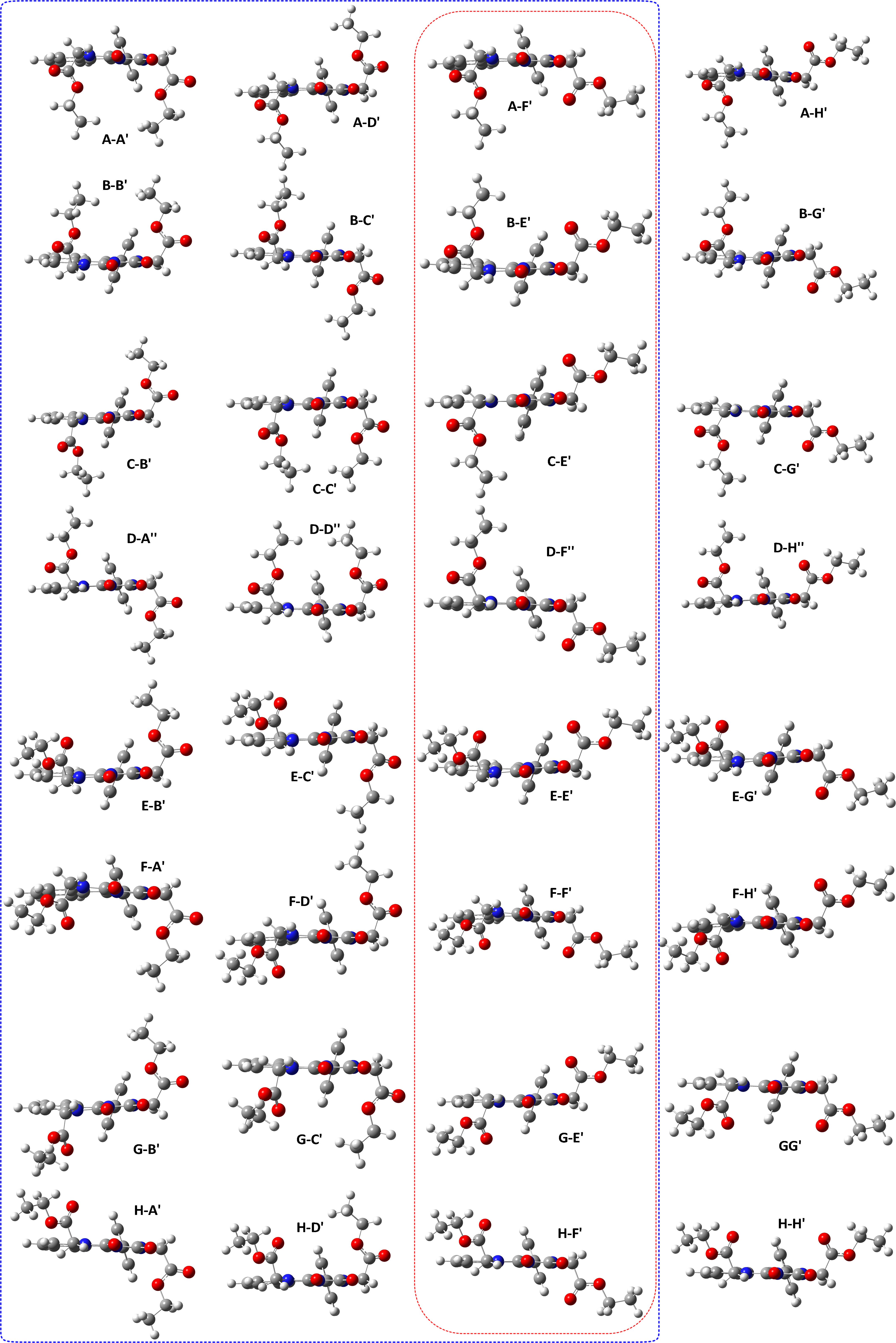
**

**Fig. 10S.** Conformers of 2,6-bis(etoxycarbonylmethyl)-1-phenylimidazo[1,5-*c*]quinazoline-3,5-dione.


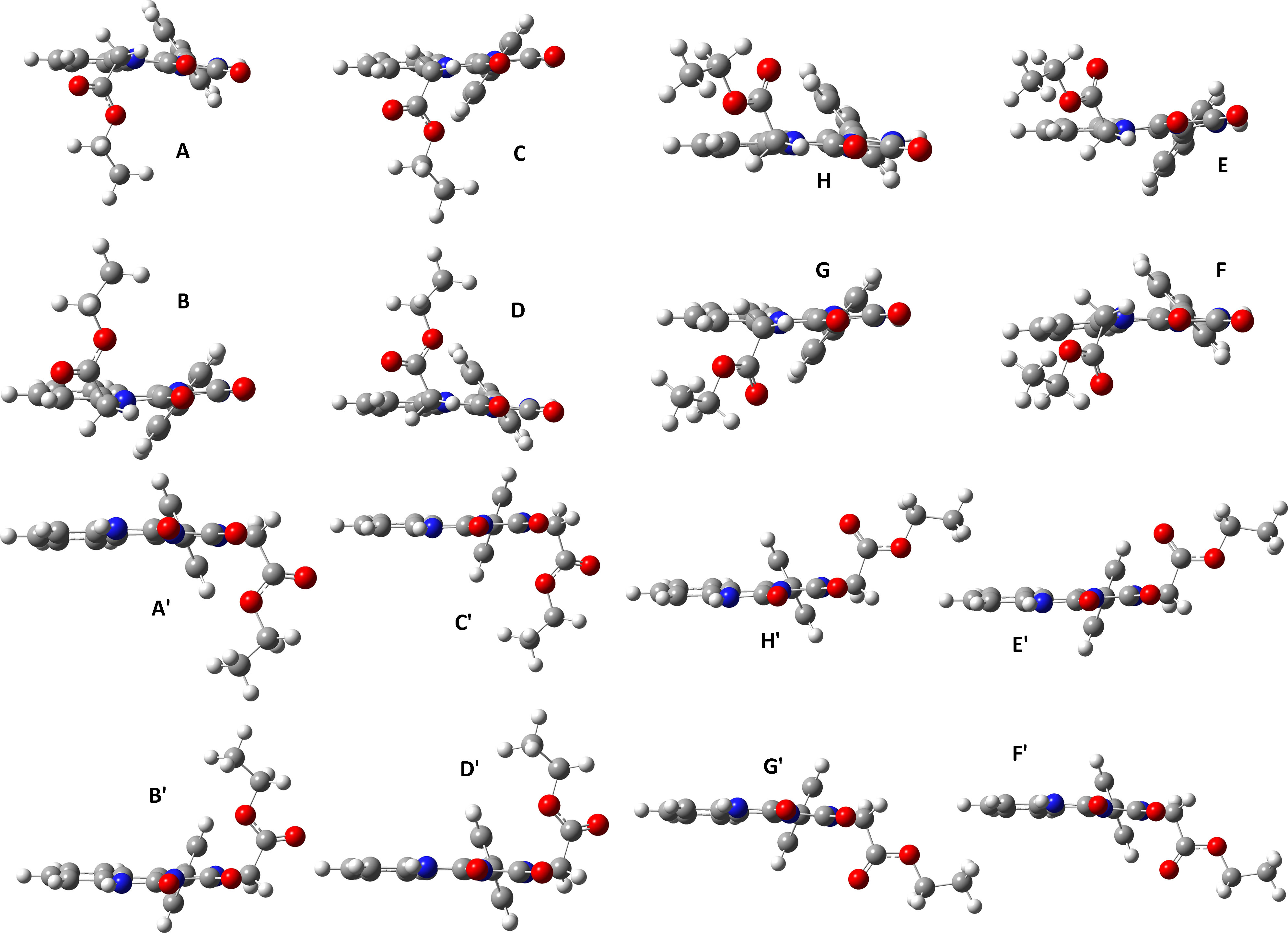


**Fig. 11S.** Conformers of 6-(etoxycarbonylmethyl)-1-phenyl-2H-imidazo[1,5-*c*]quinazoline-3,5-dione (A-H) and 2-(etoxycarbonylmethyl)-1-phenyl-6H-imidazo[1,5-*c*]quinazoline-3,5-dione (A’H’).

**Table 1S. Geometric parameters of benzene-solvated BEPIQ (Å, º) – bond lengths.**

| Bond signature | Bond length | Bond signature | Bond length |
| --- | --- | --- | --- |
| C1B—C13B | 1.357 (2) | C1A—C13A | 1.362 (2) |
| C1B—C14B | 1.484 (2) | C1A—C14A | 1.481 (2) |
| C1B—N2B | 1.399 (2) | C1A—N2A | 1.409 (2) |
| C3B—N2B | 1.371 (2) | C3A—N2A | 1.373 (2) |
| C3B—N4B | 1.409 (2) | C3A—N4A | 1.406 (2) |
| C3B—O26B | 1.218 (2) | C3A—O26A | 1.222 (2) |
| C5B—N4B | 1.399 (2) | C5A—N4A | 1.404 (2) |
| C5B—N6B | 1.377 (2) | C5A—N6A | 1.375 (2) |
| C5B—O27B | 1.213 (2) | C5A—O27A | 1.213 (2) |
| C7B—C8B | 1.401 (2) | C7A—C8A | 1.398 (3) |
| C7B—C12B | 1.409 (2) | C7A—C12A | 1.406 (2) |
| C7B—N6B | 1.421 (2) | C7A—N6A | 1.419 (2) |
| C8B—H8B | 0.9300 | C8A—H8A | 0.9300 |
| C8B—C9B | 1.383 (3) | C8A—C9A | 1.383 (3) |
| C9B—H9B | 0.9300 | C9A—H9A | 0.9300 |
| C9B—C10B | 1.387 (3) | C9A—C10A | 1.390 (3) |
| C10B—H10B | 0.9300 | C10A—H10A | 0.9300 |
| C10B—C11B | 1.383 (3) | C10A—C11A | 1.385 (3) |
| C11B—H11B | 0.9300 | C11A—H11A | 0.9300 |
| C11B—C12B | 1.400 (2) | C11A—C12A | 1.398 (3) |
| C12B—C13B | 1.445 (2) | C12A—C13A | 1.448 (2) |
| C13B—N4B | 1.420 (2) | C13A—N4A | 1.413 (2) |
| C14B—C15B | 1.401 (3) | C14A—C15A | 1.386 (3) |
| C14B—C19B | 1.393 (3) | C14A—C19A | 1.405 (3) |
| C15B—H15B | 0.9300 | C15A—H15A | 0.9300 |
| C15B—C16B | 1.387 (3) | C15A—C16A | 1.400 (3) |
| C16B—H16B | 0.9300 | C16A—H16A | 0.9300 |
| C16B—C17B | 1.381 (3) | C16A—C17A | 1.384 (4) |
| C17B—H17B | 0.9300 | C17A—H17A | 0.9300 |
| C17B—C18B | 1.392 (3) | C17A—C18A | 1.374 (3) |
| C18B—H18B | 0.9300 | C18A—H18A | 0.9300 |
| C18B—C19B | 1.392 (3) | C18A—C19A | 1.389 (3) |
| C19B—H19B | 0.9300 | C19A—H19A | 0.9300 |
| C20B—H20C | 0.9700 | C20A—H20A | 0.9700 |
| C20B—H20D | 0.9700 | C20A—H20B | 0.9700 |
| C20B—C21B | 1.517 (2) | C20A—C21A | 1.515 (2) |
| C20B—N2B | 1.450 (2) | C20A—N2A | 1.451 (2) |
| C21B—O22B | 1.329 (2) | C21A—O22A | 1.335 (2) |
| C21B—O25B | 1.207 (2) | C21A—O25A | 1.204 (2) |
| C23B—H23C | 0.9700 | C23A—H23A | 0.9700 |
| C23B—H23D | 0.9700 | C23A—H23B | 0.9700 |
| C23B—C24B | 1.508 (3) | C23A—C24A | 1.504 (3) |
| C23B—O22B | 1.461 (2) | C23A—O22A | 1.467 (2) |
| C24B—H24D | 0.9600 | C24A—H24A | 0.9600 |
| C24B—H24E | 0.9600 | C24A—H24B | 0.9600 |
| C24B—H24F | 0.9600 | C24A—H24C | 0.9600 |
| C28B—H28C | 0.9700 | C28A—H28A | 0.9700 |
| C28B—H28D | 0.9700 | C28A—H28B | 0.9700 |
| C28B—C29B | 1.512 (3) | C28A—C29A | 1.514 (3) |
| C28B—N6B | 1.452 (2) | C28A—N6A | 1.457 (2) |
| C29B—O30B | 1.341 (2) | C29A—O30A | 1.338 (2) |
| C29B—O33B | 1.197 (2) | C29A—O33A | 1.202 (2) |
| C31B—H31C | 0.9700 | C31A—H31A | 0.9700 |
| C31B—H31D | 0.9700 | C31A—H31B | 0.9700 |
| C31B—C32B | 1.495 (3) | C31A—C32A | 1.489 (3) |
| C31B—O30B | 1.454 (2) | C31A—O30A | 1.466 (2) |
| C32B—H32D | 0.9600 | C32A—H32A | 0.9600 |
| C32B—H32E | 0.9600 | C32A—H32B | 0.9600 |
| C32B—H32F | 0.9600 | C32A—H32C | 0.9600 |
|  |  |  |  |

**Table 2S. Geometric parameters of benzene-solvated BEPIQ (Å, º) – bond angles.**

| Bond signature | Bond length | Bond signature | Bond length |
| --- | --- | --- | --- |
| C13B—C1B—C14B | 131.66 (16) | C13A—C1A—C14A | 129.08 (16) |
| C13B—C1B—N2B | 107.42 (15) | C13A—C1A—N2A | 107.41 (15) |
| N2B—C1B—C14B | 120.90 (15) | N2A—C1A—C14A | 123.36 (15) |
| N2B—C3B—N4B | 103.68 (14) | N2A—C3A—N4A | 104.10 (14) |
| O26B—C3B—N2B | 126.55 (16) | O26A—C3A—N2A | 126.55 (16) |
| O26B—C3B—N4B | 129.76 (16) | O26A—C3A—N4A | 129.35 (16) |
| N6B—C5B—N4B | 114.56 (15) | N6A—C5A—N4A | 114.00 (15) |
| O27B—C5B—N4B | 122.14 (15) | O27A—C5A—N4A | 122.25 (15) |
| O27B—C5B—N6B | 123.28 (16) | O27A—C5A—N6A | 123.74 (16) |
| C8B—C7B—C12B | 119.57 (16) | C8A—C7A—C12A | 119.44 (17) |
| C8B—C7B—N6B | 120.56 (16) | C8A—C7A—N6A | 120.69 (16) |
| C12B—C7B—N6B | 119.87 (15) | C12A—C7A—N6A | 119.86 (16) |
| C7B—C8B—H8B | 119.8 | C7A—C8A—H8A | 119.7 |
| C9B—C8B—C7B | 120.33 (17) | C9A—C8A—C7A | 120.59 (17) |
| C9B—C8B—H8B | 119.8 | C9A—C8A—H8A | 119.7 |
| C8B—C9B—H9B | 119.7 | C8A—C9A—H9A | 119.8 |
| C8B—C9B—C10B | 120.62 (17) | C8A—C9A—C10A | 120.37 (18) |
| C10B—C9B—H9B | 119.7 | C10A—C9A—H9A | 119.8 |
| C9B—C10B—H10B | 120.3 | C9A—C10A—H10A | 120.3 |
| C11B—C10B—C9B | 119.41 (17) | C11A—C10A—C9A | 119.39 (18) |
| C11B—C10B—H10B | 120.3 | C11A—C10A—H10A | 120.3 |
| C10B—C11B—H11B | 119.3 | C10A—C11A—H11A | 119.4 |
| C10B—C11B—C12B | 121.45 (17) | C10A—C11A—C12A | 121.28 (17) |
| C12B—C11B—H11B | 119.3 | C12A—C11A—H11A | 119.4 |
| C7B—C12B—C13B | 118.12 (15) | C7A—C12A—C13A | 117.50 (16) |
| C11B—C12B—C7B | 118.61 (16) | C11A—C12A—C7A | 118.90 (16) |
| C11B—C12B—C13B | 123.27 (16) | C11A—C12A—C13A | 123.49 (16) |
| C1B—C13B—C12B | 135.79 (16) | C1A—C13A—C12A | 135.03 (16) |
| C1B—C13B—N4B | 106.75 (14) | C1A—C13A—N4A | 106.74 (15) |
| N4B—C13B—C12B | 117.43 (15) | N4A—C13A—C12A | 117.99 (15) |
| C15B—C14B—C1B | 119.56 (16) | C15A—C14A—C1A | 122.38 (17) |
| C19B—C14B—C1B | 120.89 (16) | C15A—C14A—C19A | 119.10 (18) |
| C19B—C14B—C15B | 119.55 (16) | C19A—C14A—C1A | 118.48 (16) |
| C14B—C15B—H15B | 119.9 | C14A—C15A—H15A | 120.0 |
| C16B—C15B—C14B | 120.26 (18) | C14A—C15A—C16A | 120.0 (2) |
| C16B—C15B—H15B | 119.9 | C16A—C15A—H15A | 120.0 |
| C15B—C16B—H16B | 120.1 | C15A—C16A—H16A | 120.0 |
| C17B—C16B—C15B | 119.86 (18) | C17A—C16A—C15A | 120.0 (2) |
| C17B—C16B—H16B | 120.1 | C17A—C16A—H16A | 120.0 |
| C16B—C17B—H17B | 119.8 | C16A—C17A—H17A | 119.6 |
| C16B—C17B—C18B | 120.49 (17) | C18A—C17A—C16A | 120.72 (19) |
| C18B—C17B—H17B | 119.8 | C18A—C17A—H17A | 119.6 |
| C17B—C18B—H18B | 120.0 | C17A—C18A—H18A | 120.2 |
| C19B—C18B—C17B | 119.94 (18) | C17A—C18A—C19A | 119.7 (2) |
| C19B—C18B—H18B | 120.0 | C19A—C18A—H18A | 120.2 |
| C14B—C19B—H19B | 120.1 | C14A—C19A—H19A | 119.7 |
| C18B—C19B—C14B | 119.88 (18) | C18A—C19A—C14A | 120.5 (2) |
| C18B—C19B—H19B | 120.1 | C18A—C19A—H19A | 119.7 |
| H20C—C20B—H20D | 108.0 | H20A—C20A—H20B | 108.0 |
| C21B—C20B—H20C | 109.4 | C21A—C20A—H20A | 109.3 |
| C21B—C20B—H20D | 109.4 | C21A—C20A—H20B | 109.3 |
| N2B—C20B—H20C | 109.4 | N2A—C20A—H20A | 109.3 |
| N2B—C20B—H20D | 109.4 | N2A—C20A—H20B | 109.3 |
| N2B—C20B—C21B | 111.22 (14) | N2A—C20A—C21A | 111.62 (14) |
| O22B—C21B—C20B | 109.71 (14) | O22A—C21A—C20A | 110.46 (15) |
| O25B—C21B—C20B | 125.25 (16) | O25A—C21A—C20A | 124.54 (16) |
| O25B—C21B—O22B | 125.04 (17) | O25A—C21A—O22A | 124.98 (17) |
| H23C—C23B—H23D | 108.1 | H23A—C23A—H23B | 108.0 |
| C24B—C23B—H23C | 109.6 | C24A—C23A—H23A | 109.4 |
| C24B—C23B—H23D | 109.6 | C24A—C23A—H23B | 109.4 |
| O22B—C23B—H23C | 109.6 | O22A—C23A—H23A | 109.4 |
| O22B—C23B—H23D | 109.6 | O22A—C23A—H23B | 109.4 |
| O22B—C23B—C24B | 110.36 (17) | O22A—C23A—C24A | 111.20 (15) |
| C23B—C24B—H24D | 109.5 | C23A—C24A—H24A | 109.5 |
| C23B—C24B—H24E | 109.5 | C23A—C24A—H24B | 109.5 |
| C23B—C24B—H24F | 109.5 | C23A—C24A—H24C | 109.5 |
| H24D—C24B—H24E | 109.5 | H24A—C24A—H24B | 109.5 |
| H24D—C24B—H24F | 109.5 | H24A—C24A—H24C | 109.5 |
| H24E—C24B—H24F | 109.5 | H24B—C24A—H24C | 109.5 |
| H28C—C28B—H28D | 107.6 | H28A—C28A—H28B | 107.9 |
| C29B—C28B—H28C | 108.7 | C29A—C28A—H28A | 109.2 |
| C29B—C28B—H28D | 108.7 | C29A—C28A—H28B | 109.2 |
| N6B—C28B—H28C | 108.7 | N6A—C28A—H28A | 109.2 |
| N6B—C28B—H28D | 108.7 | N6A—C28A—H28B | 109.2 |
| N6B—C28B—C29B | 114.07 (15) | N6A—C28A—C29A | 112.22 (15) |
| O30B—C29B—C28B | 108.40 (16) | O30A—C29A—C28A | 108.81 (16) |
| O33B—C29B—C28B | 125.61 (17) | O33A—C29A—C28A | 125.37 (17) |
| O33B—C29B—O30B | 125.98 (17) | O33A—C29A—O30A | 125.78 (18) |
| H31C—C31B—H31D | 108.2 | H31A—C31A—H31B | 108.5 |
| C32B—C31B—H31C | 109.8 | C32A—C31A—H31A | 110.2 |
| C32B—C31B—H31D | 109.8 | C32A—C31A—H31B | 110.2 |
| O30B—C31B—H31C | 109.8 | O30A—C31A—H31A | 110.2 |
| O30B—C31B—H31D | 109.8 | O30A—C31A—H31B | 110.2 |
| O30B—C31B—C32B | 109.55 (18) | O30A—C31A—C32A | 107.55 (18) |
| C31B—C32B—H32D | 109.5 | C31A—C32A—H32A | 109.5 |
| C31B—C32B—H32E | 109.5 | C31A—C32A—H32B | 109.5 |
| C31B—C32B—H32F | 109.5 | C31A—C32A—H32C | 109.5 |
| H32D—C32B—H32E | 109.5 | H32A—C32A—H32B | 109.5 |
| H32D—C32B—H32F | 109.5 | H32A—C32A—H32C | 109.5 |
| H32E—C32B—H32F | 109.5 | H32B—C32A—H32C | 109.5 |
| C1B—N2B—C20B | 127.87 (14) | C1A—N2A—C20A | 128.34 (14) |
| C3B—N2B—C1B | 112.01 (14) | C3A—N2A—C1A | 111.28 (14) |
| C3B—N2B—C20B | 120.11 (14) | C3A—N2A—C20A | 120.37 (15) |
| C3B—N4B—C13B | 110.14 (13) | C3A—N4A—C13A | 110.44 (14) |
| C5B—N4B—C3B | 124.22 (14) | C5A—N4A—C3A | 124.36 (14) |
| C5B—N4B—C13B | 125.48 (14) | C5A—N4A—C13A | 125.15 (14) |
| C5B—N6B—C7B | 124.41 (14) | C5A—N6A—C7A | 124.97 (15) |
| C5B—N6B—C28B | 115.36 (15) | C5A—N6A—C28A | 115.62 (15) |
| C7B—N6B—C28B | 120.04 (14) | C7A—N6A—C28A | 119.31 (14) |
| C21B—O22B—C23B | 116.16 (14) | C21A—O22A—C23A | 116.09 (14) |
| C29B—O30B—C31B | 117.47 (16) | C29A—O30A—C31A | 117.28 (16) |

**Table 3S. Geometric parameters of benzene-solvated BEPIQ (Å, º) – dihedral angles.**

| Bond signature | Bond length | Bond signature | Bond length |
| --- | --- | --- | --- |
| C1B—C13B—N4B—C3B | 0.42 (19) | C1A—C13A—N4A—C3A | −1.51 (19) |
| C1B—C13B—N4B—C5B | 176.03 (15) | C1A—C13A—N4A—C5A | 175.92 (15) |
| C1B—C14B—C15B—C16B | −179.07 (16) | C1A—C14A—C15A—C16A | 176.27 (17) |
| C1B—C14B—C19B—C18B | 178.72 (17) | C1A—C14A—C19A—C18A | −176.76 (17) |
| C7B—C8B—C9B—C10B | 0.0 (3) | C7A—C8A—C9A—C10A | −0.3 (3) |
| C7B—C12B—C13B—C1B | −175.27 (19) | C7A—C12A—C13A—C1A | 178.08 (19) |
| C7B—C12B—C13B—N4B | 2.5 (2) | C7A—C12A—C13A—N4A | 4.6 (2) |
| C8B—C7B—C12B—C11B | 0.0 (2) | C8A—C7A—C12A—C11A | −1.5 (3) |
| C8B—C7B—C12B—C13B | 179.43 (16) | C8A—C7A—C12A—C13A | −177.87 (16) |
| C8B—C7B—N6B—C5B | 177.39 (16) | C8A—C7A—N6A—C5A | 175.35 (16) |
| C8B—C7B—N6B—C28B | −8.0 (2) | C8A—C7A—N6A—C28A | −0.9 (2) |
| C8B—C9B—C10B—C11B | −0.1 (3) | C8A—C9A—C10A—C11A | −0.9 (3) |
| C9B—C10B—C11B—C12B | 0.1 (3) | C9A—C10A—C11A—C12A | 0.9 (3) |
| C10B—C11B—C12B—C7B | −0.1 (3) | C10A—C11A—C12A—C7A | 0.3 (3) |
| C10B—C11B—C12B—C13B | −179.47 (17) | C10A—C11A—C12A—C13A | 176.49 (17) |
| C11B—C12B—C13B—C1B | 4.1 (3) | C11A—C12A—C13A—C1A | 1.9 (3) |
| C11B—C12B—C13B—N4B | −178.14 (16) | C11A—C12A—C13A—N4A | −171.63 (16) |
| C12B—C7B—C8B—C9B | 0.0 (3) | C12A—C7A—C8A—C9A | 1.5 (3) |
| C12B—C7B—N6B—C5B | −3.3 (2) | C12A—C7A—N6A—C5A | −3.6 (3) |
| C12B—C7B—N6B—C28B | 171.33 (15) | C12A—C7A—N6A—C28A | −179.92 (16) |
| C12B—C13B—N4B—C3B | −177.93 (14) | C12A—C13A—N4A—C3A | 173.70 (15) |
| C12B—C13B—N4B—C5B | −2.3 (2) | C12A—C13A—N4A—C5A | −8.9 (2) |
| C13B—C1B—C14B—C15B | 114.8 (2) | C13A—C1A—C14A—C15A | −120.9 (2) |
| C13B—C1B—C14B—C19B | −65.4 (3) | C13A—C1A—C14A—C19A | 56.6 (3) |
| C13B—C1B—N2B—C3B | −0.3 (2) | C13A—C1A—N2A—C3A | −1.38 (19) |
| C13B—C1B—N2B—C20B | −178.87 (16) | C13A—C1A—N2A—C20A | 179.64 (16) |
| C14B—C1B—C13B—C12B | −0.4 (4) | C14A—C1A—C13A—C12A | 12.1 (3) |
| C14B—C1B—C13B—N4B | −178.24 (17) | C14A—C1A—C13A—N4A | −173.85 (17) |
| C14B—C1B—N2B—C3B | 178.09 (16) | C14A—C1A—N2A—C3A | 174.49 (16) |
| C14B—C1B—N2B—C20B | −0.5 (3) | C14A—C1A—N2A—C20A | −4.5 (3) |
| C14B—C15B—C16B—C17B | −0.2 (3) | C14A—C15A—C16A—C17A | 0.8 (3) |
| C15B—C14B—C19B—C18B | −1.5 (3) | C15A—C14A—C19A—C18A | 0.8 (3) |
| C15B—C16B—C17B—C18B | −0.4 (3) | C15A—C16A—C17A—C18A | 0.1 (3) |
| C16B—C17B—C18B—C19B | 0.0 (3) | C16A—C17A—C18A—C19A | −0.5 (3) |
| C17B—C18B—C19B—C14B | 0.9 (3) | C17A—C18A—C19A—C14A | 0.0 (3) |
| C19B—C14B—C15B—C16B | 1.2 (3) | C19A—C14A—C15A—C16A | −1.2 (3) |
| C20B—C21B—O22B—C23B | 171.42 (15) | C20A—C21A—O22A—C23A | −172.43 (14) |
| C21B—C20B—N2B—C1B | 94.1 (2) | C21A—C20A—N2A—C1A | −89.6 (2) |
| C21B—C20B—N2B—C3B | −84.36 (19) | C21A—C20A—N2A—C3A | 91.46 (19) |
| C24B—C23B—O22B—C21B | −75.2 (2) | C24A—C23A—O22A—C21A | 78.4 (2) |
| C28B—C29B—O30B—C31B | 176.91 (16) | C28A—C29A—O30A—C31A | 175.56 (17) |
| C29B—C28B—N6B—C5B | −107.00 (18) | C29A—C28A—N6A—C5A | 109.74 (18) |
| C29B—C28B—N6B—C7B | 77.9 (2) | C29A—C28A—N6A—C7A | −73.6 (2) |
| C32B—C31B—O30B—C29B | −109.0 (2) | C32A—C31A—O30A—C29A | 147.78 (19) |
| N2B—C1B—C13B—C12B | 177.83 (19) | N2A—C1A—C13A—C12A | −172.31 (18) |
| N2B—C1B—C13B—N4B | −0.07 (19) | N2A—C1A—C13A—N4A | 1.71 (18) |
| N2B—C1B—C14B—C15B | −63.2 (2) | N2A—C1A—C14A—C15A | 64.2 (2) |
| N2B—C1B—C14B—C19B | 116.59 (19) | N2A—C1A—C14A—C19A | −118.33 (19) |
| N2B—C3B—N4B—C5B | −176.27 (15) | N2A—C3A—N4A—C5A | −176.80 (15) |
| N2B—C3B—N4B—C13B | −0.59 (18) | N2A—C3A—N4A—C13A | 0.66 (18) |
| N2B—C20B—C21B—O22B | 173.93 (14) | N2A—C20A—C21A—O22A | −163.49 (14) |
| N2B—C20B—C21B—O25B | −6.0 (3) | N2A—C20A—C21A—O25A | 17.9 (3) |
| N4B—C3B—N2B—C1B | 0.55 (18) | N4A—C3A—N2A—C1A | 0.43 (18) |
| N4B—C3B—N2B—C20B | 179.23 (14) | N4A—C3A—N2A—C20A | 179.50 (14) |
| N4B—C5B—N6B—C7B | 3.4 (2) | N4A—C5A—N6A—C7A | 0.0 (2) |
| N4B—C5B—N6B—C28B | −171.41 (15) | N4A—C5A—N6A—C28A | 176.40 (14) |
| N6B—C5B—N4B—C3B | 174.46 (15) | N6A—C5A—N4A—C3A | −176.48 (15) |
| N6B—C5B—N4B—C13B | −0.6 (2) | N6A—C5A—N4A—C13A | 6.4 (2) |
| N6B—C7B—C8B—C9B | 179.38 (16) | N6A—C7A—C8A—C9A | −177.53 (16) |
| N6B—C7B—C12B—C11B | −179.35 (15) | N6A—C7A—C12A—C11A | 177.52 (16) |
| N6B—C7B—C12B—C13B | 0.1 (2) | N6A—C7A—C12A—C13A | 1.1 (2) |
| N6B—C28B—C29B—O30B | 177.88 (15) | N6A—C28A—C29A—O30A | 168.56 (15) |
| N6B—C28B—C29B—O33B | −2.4 (3) | N6A—C28A—C29A—O33A | −13.5 (3) |
| O25B—C21B—O22B—C23B | −8.6 (3) | O25A—C21A—O22A—C23A | 6.1 (3) |
| O26B—C3B—N2B—C1B | −179.78 (17) | O26A—C3A—N2A—C1A | −179.44 (16) |
| O26B—C3B—N2B—C20B | −1.1 (3) | O26A—C3A—N2A—C20A | −0.4 (3) |
| O26B—C3B—N4B—C5B | 4.1 (3) | O26A—C3A—N4A—C5A | 3.1 (3) |
| O26B—C3B—N4B—C13B | 179.77 (18) | O26A—C3A—N4A—C13A | −179.48 (17) |
| O27B—C5B—N4B—C3B | −4.0 (3) | O27A—C5A—N4A—C3A | 3.0 (3) |
| O27B—C5B—N4B—C13B | −179.06 (16) | O27A—C5A—N4A—C13A | −174.07 (16) |
| O27B—C5B—N6B—C7B | −178.11 (16) | O27A—C5A—N6A—C7A | −179.49 (17) |
| O27B—C5B—N6B—C28B | 7.1 (2) | O27A—C5A—N6A—C28A | −3.1 (2) |
| O33B—C29B—O30B—C31B | −2.8 (3) | O33A—C29A—O30A—C31A | −2.4 (3) |

**Table 4S.** The geometry of hydrogen bonds in the crystal of benzene-solvated BEPIQ.

| **D–H** | **A** | **d(D···A) (Å)** | **< D–H···A (°)** |
| --- | --- | --- | --- |
| C9A–H9A | O25B^i^ | 3.254(2) | 133 |
| C15A–H15A | O25A | 3.322(2) | 145 |
| C15B–H15B | O25B | 3.395(2) | 144 |
| C17A–H17A | O33A^ii^ | 3.279(3) | 131 |
| C20A–H20B | O26B^iii^ | 3.130(2) | 137 |
| C20A–H20B | O27B^iii^ | 3.301(2) | 145 |
| C20B–H20C | O26A^iii^ | 3.326(2) | 148 |
| C23A–H23A | O22B | 3.406(2) | 172 |
| C23B–H23C | O25A | 3.380(3) | 141 |
| C28A–H28A | O27A | 2.696(2) | 107 |
| C28B–H28D | O27B | 2.685(2) | 106 |
| C31B–H31D | O33B | 2.734(3) | 105 |
| C37A–H37A | O26B | 3.353(3) | 155 |

Symmetry codes: (i) –x + 3/2, –y + 1, –z + 1; (ii) x + 1, y, z; (iii) x + 1/2, –y + 1/2, –z + 1.

**Table 5S.** The geometry of π–π interactions in the crystal of benzene-solvated BEPIQ.

| ***Cg*I** | ***Cg*J** | ***Cg*I···*Cg*J (Å)** | **Dihedral angle (°)** | **Interplanar distance (Å)** | **Offset (Å)** |
| --- | --- | --- | --- | --- | --- |
| 1 | 10^iv^ | 3.879(2) | 6.66(9) | 3.278(2) | 2.074(2) |
| 1 | 11^v^ | 3.644(2) | 12.49(9) | -3.140(2) | 1.845(2) |
| 2 | 10^iv^ | 3.430(2) | 3.02(8) | 3.428(2) | 0.117(2) |
| 3 | 8^iv^ | 3.954(2) | 3.45(9) | 3.470(2) | 1.896(2) |
| 3 | 9^iv^ | 3.409(2) | 2.75(8) | 3.409(2) | 0.143(2) |
| 4 | 8^iii^ | 3.588(2) | 8.18(10) | -3.341(2) | 1.308(2) |
| 4 | 9^iii^ | 3.853(2) | 11.07(9) | -3.330(2) | 1.938(2) |
| 8 | 3^vi^ | 3.954(2) | 3.45(9) | 3.367(2) | 2.073(2) |
| 8 | 4^v^ | 3.588(2) | 8.18(10) | -3.121(2) | 1.170(2) |
| 9 | 3^vi^ | 3.409(2) | 2.75(8) | 3.406(2) | 0.143(2) |
| 9 | 4^v^ | 3.853(2) | 11.07(9) | -3.350(2) | 1.903(2) |
| 10 | 1^vi^ | 3.879(2) | 6.66(9) | 3.433(2) | 1.806(2) |
| 10 | 2^vi^ | 3.430(2) | 3.02(8) | 3.420(2) | 0.262(2) |
| 11 | 1^iii^ | 3.644(2) | 12.49(9) | -3.463(2) | 1.134(2) |

Symmetry codes: (iii) x + 1/2, –y + 1/2, –z + 1; (iv) –x + 3/2, –y + 1, z + 1/2; (v) x – 1/2, –y + 1/2, –z + 1; (vi) –x + 3/2, –y + 1, z – 1/2.

*Cg*1, *Cg*2, *Cg*3, *Cg*4, *Cg*8, *Cg*9, *Cg*10 and *Cg*11 denote the geometric centers of gravity of the rings delineated by the C1A–N4A/C13A, N4A–C7A/C12A/C13A, C7A–C12A, C14A–C19A, C1B–N4B/C13B, N4B–C7B/C12B/C13B, C7B–C12B, C14B–C19B atoms, respectively (Fig. 1S).

*Cg*I···*Cg*J is the distance between ring centroids.

The dihedral angle is the angle between the mean planes of *Cg*I and *Cg*J.

The interplanar distance is the perpendicular distance from *Cg*I to ring J.

The offset is the perpendicular distance from ring I to ring J.

**Table 6S.** The geometry of C=O···π contacts in the crystal of benzene-solvated BEPIQ.

| **Y–X** | ***Cg*I** | **X···*Cg*I (Å)** | **< Y–X···*Cg*I (°)** |
| --- | --- | --- | --- |
| C3A–O26A | 11^v^ | 3.741(2) | 68.50(10) |
| C3B–O26B | 4^v^ | 3.858(2) | 64.74(10) |
| C5B–O27B | 4^v^ | 3.400(2) | 67.32(10) |

Symmetry code: (v) x – 1/2, –y + 1/2, –z + 1.

*Cg*4 and *Cg*11 denote the geometric centers of gravity of the rings delineated by the C14A–C19AC14B–C19B atoms, respectively (Fig. 1S).

**Tab. 7S. The comparison of the bond lengths of BEPIQ calculated by Gaussian and measured in crystal.**

|  | Bond length [Å] | |
| --- | --- | --- |
| Bond signature | crystal measured | calculated in Gaussian |
| C1B—C13B | 1.357 | 1.357 |
| C1B—C14B | 1.484 | 1.476 |
| C1B—N2B | 1.399 | 1.403 |
| C3B—N2B | 1.371 | 1.385 |
| C3B—N4B | 1.409 | 1.415 |
| C3B—O26B | 1.218 | 1.213 |
| C5B—N4B | 1.399 | 1.398 |
| C5B—N6B | 1.377 | 1.393 |
| C5B—O27B | 1.213 | 1.212 |
| C7B—C8B | 1.401 | 1.401 |
| C7B—C12B | 1.409 | 1.412 |
| C7B—N6B | 1.421 | 1.415 |
| C8B—H8B | 0.930 | 1.080 |
| C8B—C9B | 1.383 | 1.391 |
| C9B—H9B | 0.930 | 1.084 |
| C9B—C10B | 1.387 | 1.394 |
| C10B—H10B | 0.930 | 1.083 |
| C10B—C11B | 1.383 | 1.388 |
| C11B—H11B | 0.930 | 1.081 |
| C11B—C12B | 1.400 | 1.403 |
| C12B—C13B | 1.445 | 1.445 |
| C13B—N4B | 1.420 | 1.419 |
| C14B—C15B | 1.401 | 1.401 |
| C14B—C19B | 1.393 | 1.401 |
| C15B—H15B | 0.930 | 1.083 |
| C15B—C16B | 1.387 | 1.394 |
| C16B—H16B | 0.930 | 1.084 |
| C16B—C17B | 1.381 | 1.395 |
| C17B—H17B | 0.930 | 1.084 |
| C17B—C18B | 1.392 | 1.395 |
| C18B—H18B | 0.930 | 1.084 |
| C18B—C19B | 1.392 | 1.393 |
| C19B—H19B | 0.930 | 1.084 |
| C20B—H20C | 0.970 | 1.091 |
| C20B—H20D | 0.970 | 1.091 |
| C20B—C21B | 1.517 | 1.526 |
| C20B—N2B | 1.450 | 1.440 |
| C21B—O22B | 1.329 | 1.337 |
| C21B—O25B | 1.207 | 1.206 |
| C23B—H23C | 0.970 | 1.092 |
| C23B—H23D | 0.970 | 1.092 |
| C23B—C24B | 1.508 | 1.513 |
| C23B—O22B | 1.461 | 1.457 |
| C24B—H24D | 0.960 | 1.092 |
| C24B—H24E | 0.960 | 1.092 |
| C24B—H24F | 0.960 | 1.093 |
| C28B—H28C | 0.970 | 1.090 |
| C28B—H28D | 0.970 | 1.089 |
| C28B—C29B | 1.512 | 1.524 |
| C28B—N6B | 1.452 | 1.452 |
| C29B—O30B | 1.341 | 1.340 |
| C29B—O33B | 1.197 | 1.205 |
| C31B—H31C | 0.970 | 1.092 |
| C31B—H31D | 0.970 | 1.092 |
| C31B—C32B | 1.495 | 1.513 |
| C31B—O30B | 1.454 | 1.457 |
| C32B—H32D | 0.960 | 1.093 |
| C32B—H32E | 0.960 | 1.092 |
| C32B—H32F | 0.960 | 1.092 |
| C1A—C13A | 1.362 | 1.361 |
| C1A—C14A | 1.481 | 1.477 |
| C1A—N2A | 1.409 | 1.405 |
| C3A—N2A | 1.373 | 1.385 |
| C3A—N4A | 1.406 | 1.415 |
| C3A—O26A | 1.222 | 1.221 |
| C5A—N4A | 1.404 | 1.398 |
| C5A—N6A | 1.375 | 1.393 |
| C5A—O27A | 1.213 | 1.220 |
| C7A—C8A | 1.398 | 1.404 |
| C7A—C12A | 1.406 | 1.415 |
| C7A—N6A | 1.419 | 1.416 |
| C8A—H8A | 0.930 | 1.082 |
| C8A—C9A | 1.383 | 1.394 |
| C9A—H9A | 0.930 | 1.085 |
| C9A—C10A | 1.390 | 1.397 |
| C10A—H10A | 0.930 | 1.085 |
| C10A—C11A | 1.385 | 1.391 |
| C11A—H11A | 0.930 | 1.083 |
| C11A—C12A | 1.398 | 1.405 |
| C12A—C13A | 1.448 | 1.447 |
| C13A—N4A | 1.413 | 1.420 |
| C14A—C15A | 1.386 | 1.404 |
| C14A—C19A | 1.405 | 1.404 |
| C15A—H15A | 0.930 | 1.085 |
| C15A—C16A | 1.400 | 1.397 |
| C16A—H16A | 0.930 | 1.086 |
| C16A—C17A | 1.384 | 1.399 |
| C17A—H17A | 0.930 | 1.086 |
| C17A—C18A | 1.374 | 1.399 |
| C18A—H18A | 0.930 | 1.086 |
| C18A—C19A | 1.389 | 1.397 |
| C19A—H19A | 0.930 | 1.086 |
| C20A—H20A | 0.970 | 1.093 |
| C20A—H20B | 0.970 | 1.093 |
| C20A—C21A | 1.515 | 1.527 |
| C20A—N2A | 1.451 | 1.440 |
| C21A—O22A | 1.335 | 1.339 |
| C21A—O25A | 1.204 | 1.214 |
| C23A—H23A | 0.970 | 1.094 |
| C23A—H23B | 0.970 | 1.094 |
| C23A—C24A | 1.504 | 1.515 |
| C23A—O22A | 1.467 | 1.458 |
| C24A—H24A | 0.960 | 1.095 |
| C24A—H24B | 0.960 | 1.094 |
| C24A—H24C | 0.960 | 1.094 |
| C28A—H28A | 0.970 | 1.091 |
| C28A—H28B | 0.970 | 1.092 |
| C28A—C29A | 1.514 | 1.525 |
| C28A—N6A | 1.457 | 1.453 |
| C29A—O30A | 1.338 | 1.342 |
| C29A—O33A | 1.202 | 1.213 |
| C31A—H31A | 0.970 | 1.094 |
| C31A—H31B | 0.970 | 1.094 |
| C31A—C32A | 1.489 | 1.515 |
| C31A—O30A | 1.466 | 1.458 |
| C32A—H32A | 0.960 | 1.094 |
| C32A—H32B | 0.960 | 1.094 |
| C32A—H32C | 0.960 | 1.095 |

**Tab. 8S.** Angles value of BEPIQ calculated by Gaussian and measured in crystal

|  | Angle value [°] | |
| --- | --- | --- |
| Angle signature | crystal measured | calculated in Gaussian |
| C13B—C1B—C14B | 131.660 | 131.200 |
| C13B—C1B—N2B | 107.420 | 107.285 |
| N2B—C1B—C14B | 120.900 | 121.496 |
| N2B—C3B—N4B | 103.680 | 103.564 |
| O26B—C3B—N2B | 126.550 | 126.550 |
| O26B—C3B—N4B | 129.760 | 129.885 |
| N6B—C5B—N4B | 114.560 | 114.436 |
| O27B—C5B—N4B | 122.140 | 122.751 |
| O27B—C5B—N6B | 123.280 | 122.811 |
| C8B—C7B—C12B | 119.570 | 119.316 |
| C8B—C7B—N6B | 120.560 | 120.893 |
| C12B—C7B—N6B | 119.870 | 119.791 |
| C7B—C8B—H8B | 119.800 | 120.930 |
| C9B—C8B—C7B | 120.330 | 120.576 |
| C9B—C8B—H8B | 119.800 | 118.493 |
| C8B—C9B—H9B | 119.700 | 119.213 |
| C8B—C9B—C10B | 120.620 | 120.320 |
| C10B—C9B—H9B | 119.700 | 120.467 |
| C9B—C10B—H10B | 120.300 | 120.572 |
| C11B—C10B—C9B | 119.410 | 119.524 |
| C11B—C10B—H10B | 120.300 | 119.903 |
| C10B—C11B—H11B | 119.300 | 119.747 |
| C10B—C11B—C12B | 121.450 | 121.102 |
| C12B—C11B—H11B | 119.300 | 119.151 |
| C7B—C12B—C13B | 118.120 | 117.740 |
| C11B—C12B—C7B | 118.610 | 119.144 |
| C11B—C12B—C13B | 123.270 | 123.115 |
| C1B—C13B—C12B | 135.790 | 134.462 |
| C1B—C13B—N4B | 106.750 | 107.256 |
| N4B—C13B—C12B | 117.430 | 118.265 |
| C15B—C14B—C1B | 119.560 | 120.584 |
| C19B—C14B—C1B | 120.890 | 120.016 |
| C19B—C14B—C15B | 119.550 | 119.396 |
| C14B—C15B—H15B | 119.900 | 119.274 |
| C16B—C15B—C14B | 120.260 | 120.140 |
| C16B—C15B—H15B | 119.900 | 120.585 |
| C15B—C16B—H16B | 120.100 | 119.659 |
| C17B—C16B—C15B | 119.860 | 120.210 |
| C17B—C16B—H16B | 120.100 | 120.130 |
| C16B—C17B—H17B | 119.800 | 120.079 |
| C16B—C17B—C18B | 120.490 | 119.893 |
| C18B—C17B—H17B | 119.800 | 120.028 |
| C17B—C18B—H18B | 120.000 | 120.182 |
| C19B—C18B—C17B | 119.940 | 120.058 |
| C19B—C18B—H18B | 120.000 | 119.760 |
| C14B—C19B—H19B | 120.100 | 119.306 |
| C18B—C19B—C14B | 119.880 | 120.301 |
| C18B—C19B—H19B | 120.100 | 120.391 |
| H20C—C20B—H20D | 108.000 | 108.256 |
| C21B—C20B—H20C | 109.400 | 108.777 |
| C21B—C20B—H20D | 109.400 | 108.578 |
| N2B—C20B—H20C | 109.400 | 110.089 |
| N2B—C20B—H20D | 109.400 | 108.510 |
| N2B—C20B—C21B | 111.220 | 112.532 |
| O22B—C21B—C20B | 109.710 | 109.884 |
| O25B—C21B—C20B | 125.250 | 125.143 |
| O25B—C21B—O22B | 125.040 | 124.970 |
| H23C—C23B—H23D | 108.100 | 108.257 |
| C24B—C23B—H23C | 109.600 | 112.368 |
| C24B—C23B—H23D | 109.600 | 112.330 |
| O22B—C23B—H23C | 109.600 | 108.226 |
| O22B—C23B—H23D | 109.600 | 108.204 |
| O22B—C23B—C24B | 110.360 | 107.302 |
| C23B—C24B—H24D | 109.500 | 110.958 |
| C23B—C24B—H24E | 109.500 | 110.865 |
| C23B—C24B—H24F | 109.500 | 109.447 |
| H24D—C24B—H24E | 109.500 | 108.649 |
| H24D—C24B—H24F | 109.500 | 108.431 |
| H24E—C24B—H24F | 109.500 | 108.419 |
| H28C—C28B—H28D | 107.600 | 107.436 |
| C29B—C28B—H28C | 108.700 | 110.411 |
| C29B—C28B—H28D | 108.700 | 107.465 |
| N6B—C28B—H28C | 108.700 | 111.130 |
| N6B—C28B—H28D | 108.700 | 107.873 |
| N6B—C28B—C29B | 114.070 | 112.302 |
| O30B—C29B—C28B | 108.400 | 109.994 |
| O33B—C29B—C28B | 125.610 | 125.080 |
| O33B—C29B—O30B | 125.980 | 124.913 |
| H31C—C31B—H31D | 108.200 | 108.205 |
| C32B—C31B—H31C | 109.800 | 112.324 |
| C32B—C31B—H31D | 109.800 | 112.352 |
| O30B—C31B—H31C | 109.800 | 108.239 |
| O30B—C31B—H31D | 109.800 | 108.269 |
| O30B—C31B—C32B | 109.550 | 107.302 |
| C31B—C32B—H32D | 109.500 | 109.467 |
| C31B—C32B—H32E | 109.500 | 110.949 |
| C31B—C32B—H32F | 109.500 | 110.905 |
| H32D—C32B—H32E | 109.500 | 108.401 |
| H32D—C32B—H32F | 109.500 | 108.413 |
| H32E—C32B—H32F | 109.500 | 108.632 |
| C1B—N2B—C20B | 127.870 | 126.940 |
| C3B—N2B—C1B | 112.010 | 111.798 |
| C3B—N2B—C20B | 120.110 | 121.173 |
| C3B—N4B—C13B | 110.140 | 110.083 |
| C5B—N4B—C3B | 124.220 | 124.858 |
| C5B—N4B—C13B | 125.480 | 125.006 |
| C5B—N6B—C7B | 124.410 | 124.621 |
| C5B—N6B—C28B | 115.360 | 115.530 |
| C7B—N6B—C28B | 120.040 | 119.763 |
| C21B—O22B—C23B | 116.160 | 116.726 |
| C29B—O30B—C31B | 117.470 | 116.633 |
| C13A—C1A—C14A | 129.080 | 131.288 |
| C13A—C1A—N2A | 107.410 | 107.283 |
| N2A—C1A—C14A | 123.360 | 121.470 |
| N2A—C3A—N4A | 104.100 | 103.565 |
| O26A—C3A—N2A | 126.550 | 126.537 |
| O26A—C3A—N4A | 129.350 | 129.895 |
| N6A—C5A—N4A | 114.000 | 114.435 |
| O27A—C5A—N4A | 122.250 | 122.755 |
| O27A—C5A—N6A | 123.740 | 122.808 |
| C8A—C7A—C12A | 119.440 | 119.310 |
| C8A—C7A—N6A | 120.690 | 120.897 |
| C12A—C7A—N6A | 119.860 | 119.792 |
| C7A—C8A—H8A | 119.700 | 120.937 |
| C9A—C8A—C7A | 120.590 | 120.573 |
| C9A—C8A—H8A | 119.700 | 118.490 |
| C8A—C9A—H9A | 119.800 | 119.211 |
| C8A—C9A—C10A | 120.370 | 120.327 |
| C10A—C9A—H9A | 119.800 | 120.462 |
| C9A—C10A—H10A | 120.300 | 120.573 |
| C11A—C10A—C9A | 119.390 | 119.520 |
| C11A—C10A—H10A | 120.300 | 119.906 |
| C10A—C11A—H11A | 119.400 | 119.754 |
| C10A—C11A—C12A | 121.280 | 121.100 |
| C12A—C11A—H11A | 119.400 | 119.146 |
| C7A—C12A—C13A | 117.500 | 117.741 |
| C11A—C12A—C7A | 118.900 | 119.151 |
| C11A—C12A—C13A | 123.490 | 123.107 |
| C1A—C13A—C12A | 135.030 | 134.456 |
| C1A—C13A—N4A | 106.740 | 107.258 |
| N4A—C13A—C12A | 117.990 | 118.270 |
| C15A—C14A—C1A | 122.380 | 120.561 |
| C15A—C14A—C19A | 119.100 | 119.400 |
| C19A—C14A—C1A | 118.480 | 120.034 |
| C14A—C15A—H15A | 120.000 | 119.275 |
| C14A—C15A—C16A | 120.000 | 120.137 |
| C16A—C15A—H15A | 120.000 | 120.587 |
| C15A—C16A—H16A | 120.000 | 119.661 |
| C17A—C16A—C15A | 120.000 | 120.211 |
| C17A—C16A—H16A | 120.000 | 120.128 |
| C16A—C17A—H17A | 119.600 | 120.077 |
| C18A—C17A—C16A | 120.720 | 119.895 |
| C18A—C17A—H17A | 119.600 | 120.027 |
| C17A—C18A—H18A | 120.200 | 120.183 |
| C17A—C18A—C19A | 119.700 | 120.056 |
| C19A—C18A—H18A | 120.200 | 119.761 |
| C14A—C19A—H19A | 119.700 | 119.308 |
| C18A—C19A—C14A | 120.500 | 120.299 |
| C18A—C19A—H19A | 119.700 | 120.382 |
| H20A—C20A—H20B | 108.000 | 108.253 |
| C21A—C20A—H20A | 109.300 | 108.578 |
| C21A—C20A—H20B | 109.300 | 108.799 |
| N2A—C20A—H20A | 109.300 | 108.499 |
| N2A—C20A—H20B | 109.300 | 110.101 |
| N2A—C20A—C21A | 111.620 | 112.511 |
| O22A—C21A—C20A | 110.460 | 109.896 |
| O25A—C21A—C20A | 124.540 | 125.132 |
| O25A—C21A—O22A | 124.980 | 124.968 |
| H23A—C23A—H23B | 108.000 | 108.258 |
| C24A—C23A—H23A | 109.400 | 112.333 |
| C24A—C23A—H23B | 109.400 | 112.365 |
| O22A—C23A—H23A | 109.400 | 108.227 |
| O22A—C23A—H23B | 109.400 | 108.202 |
| O22A—C23A—C24A | 111.200 | 107.301 |
| C23A—C24A—H24A | 109.500 | 109.446 |
| C23A—C24A—H24B | 109.500 | 110.957 |
| C23A—C24A—H24C | 109.500 | 110.864 |
| H24A—C24A—H24B | 109.500 | 108.432 |
| H24A—C24A—H24C | 109.500 | 108.420 |
| H24B—C24A—H24C | 109.500 | 108.652 |
| H28A—C28A—H28B | 107.900 | 107.444 |
| C29A—C28A—H28A | 109.200 | 107.468 |
| C29A—C28A—H28B | 109.200 | 110.413 |
| N6A—C28A—H28A | 109.200 | 111.116 |
| N6A—C28A—H28B | 109.200 | 107.886 |
| N6A—C28A—C29A | 112.220 | 112.292 |
| O30A—C29A—C28A | 108.810 | 109.999 |
| O33A—C29A—C28A | 125.370 | 125.079 |
| O33A—C29A—O30A | 125.780 | 124.910 |
| H31A—C31A—H31B | 108.500 | 108.203 |
| C32A—C31A—H31A | 110.200 | 112.348 |
| C32A—C31A—H31B | 110.200 | 112.325 |
| O30A—C31A—H31A | 110.200 | 108.270 |
| O30A—C31A—H31B | 110.200 | 108.241 |
| O30A—C31A—C32A | 107.550 | 107.305 |
| C31A—C32A—H32A | 109.500 | 110.910 |
| C31A—C32A—H32B | 109.500 | 110.950 |
| C31A—C32A—H32C | 109.500 | 109.463 |
| H32A—C32A—H32B | 109.500 | 108.633 |
| H32A—C32A—H32C | 109.500 | 108.412 |
| H32B—C32A—H32C | 109.500 | 108.399 |
| C1A—N2A—C20A | 128.340 | 126.941 |
| C3A—N2A—C1A | 111.280 | 111.801 |
| C3A—N2A—C20A | 120.370 | 121.161 |
| C3A—N4A—C13A | 110.440 | 110.079 |
| C5A—N4A—C3A | 124.360 | 124.862 |
| C5A—N4A—C13A | 125.150 | 125.015 |
| C5A—N6A—C7A | 124.970 | 124.624 |
| C5A—N6A—C28A | 115.620 | 115.534 |
| C7A—N6A—C28A | 119.310 | 119.755 |
| C21A—O22A—C23A | 116.090 | 116.722 |
| C29A—O30A—C31A | 117.280 | 116.633 |

**Tab. 9S.** Dihedral angles value of BEPIQ calculated by Gaussian and measured in crystal.

|  | Dihedral angle value [°] | |
| --- | --- | --- |
| Dihedral angle signature | crystal measured | calculated in Gaussian |
| C1B—C13B—N4B—C3B | 0.420 | 0.800 |
| C1B—C13B—N4B—C5B | 176.030 | -176.650 |
| C1B—C14B—C15B—C16B | -179.070 | 179.605 |
| C1B—C14B—C19B—C18B | 178.720 | -179.831 |
| C7B—C8B—C9B—C10B | 0.000 | -0.081 |
| C7B—C12B—C13B—C1B | -175.270 | 178.425 |
| C7B—C12B—C13B—N4B | 2.500 | -3.335 |
| C8B—C7B—C12B—C11B | 0.000 | 1.642 |
| C8B—C7B—C12B—C13B | 179.430 | -178.779 |
| C8B—C7B—N6B—C5B | 177.390 | 179.708 |
| C8B—C7B—N6B—C28B | -8.000 | 3.246 |
| C8B—C9B—C10B—C11B | -0.100 | 0.647 |
| C9B—C10B—C11B—C12B | 0.100 | -0.048 |
| C10B—C11B—C12B—C7B | -0.100 | -1.099 |
| C10B—C11B—C12B—C13B | -179.470 | 179.346 |
| C11B—C12B—C13B—C1B | 4.100 | -2.014 |
| C11B—C12B—C13B—N4B | -178.140 | 176.226 |
| C12B—C7B—C8B—C9B | 0.000 | -1.071 |
| C12B—C7B—N6B—C5B | -3.300 | -0.520 |
| C12B—C7B—N6B—C28B | 171.330 | -176.982 |
| C12B—C13B—N4B—C3B | -177.930 | -177.885 |
| C12B—C13B—N4B—C5B | -2.300 | 4.666 |
| C13B—C1B—C14B—C15B | 114.800 | 107.504 |
| C13B—C1B—C14B—C19B | -65.400 | 107.504 |
| C13B—C1B—N2B—C3B | -0.300 | -0.703 |
| C13B—C1B—N2B—C20B | -178.870 | -177.281 |
| C14B—C1B—C13B—C12B | -0.400 | -3.309 |
| C14B—C1B—C13B—N4B | -178.240 | 178.315 |
| C14B—C1B—N2B—C3B | 178.090 | -179.279 |
| C14B—C1B—N2B—C20B | -0.500 | 4.143 |
| C14B—C15B—C16B—C17B | -0.200 | 0.030 |
| C15B—C14B—C19B—C18B | -1.500 | -0.517 |
| C15B—C16B—C17B—C18B | -0.400 | -0.136 |
| C16B—C17B—C18B—C19B | 0.000 | -0.086 |
| C17B—C18B—C19B—C14B | 0.900 | 0.415 |
| C19B—C14B—C15B—C16B | 1.200 | 0.295 |
| C20B—C21B—O22B—C23B | 171.420 | 179.977 |
| C21B—C20B—N2B—C1B | 94.100 | 85.464 |
| C21B—C20B—N2B—C3B | -84.360 | -90.822 |
| C24B—C23B—O22B—C21B | -75.200 | 178.889 |
| C28B—C29B—O30B—C31B | 176.910 | -176.618 |
| C29B—C28B—N6B—C5B | -107.000 | -103.139 |
| C29B—C28B—N6B—C7B | 77.900 | 73.635 |
| C32B—C31B—O30B—C29B | -109.000 | 179.736 |
| N2B—C1B—C13B—C12B | 177.830 | 178.305 |
| N2B—C1B—C13B—N4B | -0.070 | -0.072 |
| N2B—C1B—C14B—C15B | -63.200 | -74.303 |
| N2B—C1B—C14B—C19B | 116.590 | 105.002 |
| N2B—C3B—N4B—C5B | -176.270 | 176.280 |
| N2B—C3B—N4B—C13B | -0.590 | -1.174 |
| N2B—C20B—C21B—O22B | 173.930 | 176.593 |
| N2B—C20B—C21B—O25B | -6.000 | -4.073 |
| N4B—C3B—N2B—C1B | 0.550 | 1.147 |
| N4B—C3B—N2B—C20B | 179.230 | 177.950 |
| N4B—C5B—N6B—C7B | 3.400 | 1.442 |
| N4B—C5B—N6B—C28B | -171.410 | 178.039 |
| N6B—C5B—N4B—C3B | 174.460 | 179.325 |
| N6B—C5B—N4B—C13B | -0.600 | -3.594 |
| N6B—C7B—C8B—C9B | 179.380 | 178.702 |
| N6B—C7B—C12B—C11B | -179.350 | -178.133 |
| N6B—C7B—C12B—C13B | 0.100 | 1.445 |
| N6B—C28B—C29B—O30B | 177.880 | -165.726 |
| N6B—C28B—C29B—O33B | -2.400 | 15.507 |
| O25B—C21B—O22B—C23B | -8.600 | 0.641 |
| O26B—C3B—N2B—C1B | -179.780 | -179.343 |
| O26B—C3B—N2B—C20B | -1.100 | -2.539 |
| O26B—C3B—N4B—C5B | 4.100 | -3.207 |
| O26B—C3B—N4B—C13B | 179.770 | 179.338 |
| O27B—C5B—N4B—C3B | -4.000 | -0.154 |
| O27B—C5B—N4B—C13B | -179.060 | 176.927 |
| O27B—C5B—N6B—C7B | -178.110 | -179.079 |
| O27B—C5B—N6B—C28B | 7.100 | -2.482 |
| O33B—C29B—O30B—C31B | -2.800 | 2.151 |
| C1A—C13A—N4A—C3A | -1.510 | -0.753 |
| C1A—C13A—N4A—C5A | 175.920 | 176.925 |
| C1A—C14A—C15A—C16A | 176.270 | -179.530 |
| C1A—C14A—C19A—C18A | -176.760 | 179.752 |
| C7A—C8A—C9A—C10A | -0.300 | 0.079 |
| C7A—C12A—C13A—C1A | 178.080 | -178.484 |
| C7A—C12A—C13A—N4A | 4.600 | 3.178 |
| C8A—C7A—C12A—C11A | -1.500 | -1.649 |
| C8A—C7A—C12A—C13A | -177.870 | 178.735 |
| C8A—C7A—N6A—C5A | 175.350 | -179.602 |
| C8A—C7A—N6A—C28A | -0.900 | -3.132 |
| C8A—C9A—C10A—C11A | -0.900 | -0.654 |
| C9A—C10A—C11A—C12A | 0.900 | 0.057 |
| C10A—C11A—C12A—C7A | 0.300 | 1.097 |
| C10A—C11A—C12A—C13A | 176.490 | -179.309 |
| C11A—C12A—C13A—C1A | 1.900 | 1.916 |
| C11A—C12A—C13A—N4A | -171.630 | -176.422 |
| C12A—C7A—C8A—C9A | 1.500 | 1.079 |
| C12A—C7A—N6A—C5A | -3.600 | 0.641 |
| C12A—C7A—N6A—C28A | -179.920 | 177.110 |
| C12A—C13A—N4A—C3A | 173.700 | 178.004 |
| C12A—C13A—N4A—C5A | -8.900 | -4.317 |
| C13A—C1A—C14A—C15A | -120.900 | -107.306 |
| C13A—C1A—C14A—C19A | 56.600 | 73.451 |
| C13A—C1A—N2A—C3A | -1.380 | 0.716 |
| C13A—C1A—N2A—C20A | 179.640 | 177.147 |
| C14A—C1A—C13A—C12A | 12.100 | 3.191 |
| C14A—C1A—C13A—N4A | -173.850 | -178.342 |
| C14A—C1A—N2A—C3A | 174.490 | 179.286 |
| C14A—C1A—N2A—C20A | -4.500 | -4.282 |
| C14A—C15A—C16A—C17A | 0.800 | -0.025 |
| C15A—C14A—C19A—C18A | 0.800 | 0.500 |
| C15A—C16A—C17A—C18A | 0.100 | 0.118 |
| C16A—C17A—C18A—C19A | -0.500 | 0.099 |
| C17A—C18A—C19A—C14A | 0.000 | -0.410 |
| C19A—C14A—C15A—C16A | -1.200 | -0.282 |
| C20A—C21A—O22A—C23A | -172.430 | 179.971 |
| C21A—C20A—N2A—C1A | -89.600 | -85.382 |
| C21A—C20A—N2A—C3A | 91.460 | 90.746 |
| C24A—C23A—O22A—C21A | 78.400 | -178.920 |
| C28A—C29A—O30A—C31A | 175.560 | 176.654 |
| C29A—C28A—N6A—C5A | 109.740 | 103.125 |
| C29A—C28A—N6A—C7A | -73.600 | -73.656 |
| C32A—C31A—O30A—C29A | 147.780 | -179.834 |
| N2A—C1A—C13A—C12A | -172.310 | -178.431 |
| N2A—C1A—C13A—N4A | 1.710 | 0.037 |
| N2A—C1A—C14A—C15A | 64.200 | 74.509 |
| N2A—C1A—C14A—C19A | -118.330 | -104.734 |
| N2A—C3A—N4A—C5A | -176.800 | -176.546 |
| N2A—C3A—N4A—C13A | 0.660 | 1.137 |
| N2A—C20A—C21A—O22A | -163.490 | -176.615 |
| N2A—C20A—C21A—O25A | 17.900 | 4.024 |
| N4A—C3A—N2A—C1A | 0.430 | -1.131 |
| N4A—C3A—N2A—C20A | 179.500 | -177.798 |
| N4A—C5A—N6A—C7A | 0.000 | -1.398 |
| N4A—C5A—N6A—C28A | 176.400 | -178.002 |
| N6A—C5A—N4A—C3A | -176.480 | -179.348 |
| N6A—C5A—N4A—C13A | 6.400 | 3.309 |
| N6A—C7A—C8A—C9A | -177.530 | -178.679 |
| N6A—C7A—C12A—C11A | 177.520 | 178.112 |
| N6A—C7A—C12A—C13A | 1.100 | -1.504 |
| N6A—C28A—C29A—O30A | 168.560 | 165.644 |
| N6A—C28A—C29A—O33A | -13.500 | -15.581 |
| O25A—C21A—O22A—C23A | 6.100 | -0.667 |
| O26A—C3A—N2A—C1A | -179.440 | 179.380 |
| O26A—C3A—N2A—C20A | -0.400 | 2.713 |
| O26A—C3A—N4A—C5A | 3.100 | 2.918 |
| O26A—C3A—N4A—C13A | -179.480 | -179.399 |
| O27A—C5A—N4A—C3A | 3.000 | 0.121 |
| O27A—C5A—N4A—C13A | -174.070 | -177.222 |
| O27A—C5A—N6A—C7A | -179.490 | 2.530 |
| O27A—C5A—N6A—C28A | -3.100 | 179.133 |
| O33A—C29A—O30A—C31A | -2.400 | -2.123 |

**Tab. 10S.** Absolute energies of conformers MEPIQ and BEPIQ.

| Conformer | Absolute energy [kJ/mol] | Relative energy [kJ/mol]  |E_min_-E| |
| --- | --- | --- |
| A | -3248576.5528 | 18.8983 ^a^ |
| B | -3248576.4530 | 18.9981 ^a^ |
| C | -3248576.0566 | 19.3945 ^a^ |
| D | -3248575.9620 | 19.4891 ^a^ |
| E | -3248577.9679 | 17.4832 ^a^ |
| F | -3248577.8865 | 15.5646 ^a^ |
| G | -3248577.0542 | 18.3969 ^a^ |
| H | -3248577.2118 | 18.2393 ^a^ |
| A’ | -3248591.7833 | 3.6678 ^a^ |
| B’ | -3248591.2556 | 4.1955 ^a^ |
| C’ | -3248591.7885 | 3.6626 ^a^ |
| D’ | -3248591.7912 | 3.6599 ^a^ |
| E’ | -3248595.3093 | 0.1418 ^a^ |
| F’ | -3248595.2778 | 0.1733 ^a^ |
| G’ | -3248595.3592 | 0.0919 ^a^ |
| H’ | -3248595.4511 | 0.0000 ^a^ |
| A-F’ | -4053146.2200 | 2.2946 ^b^ |
| B-E’ | -4053146.1753 | 2.3393 ^b^ |
| C-E’ | -4053145.4271 | 3.0875 ^b^ |
| D-F’ | -4053145.4034 | 3.1112 ^b^ |
| E-E’ | -4053147.3699 | 1.1447 ^b^ |
| F-F’ | -4053147.0444 | 1.4702 ^b^ |
| G-E’ | -4053148.5146 | 0.0000 ^b^ |
| H-F’ | -4053147.7112 | 0.8034 ^b^ |

^a^ relative to H’ conformer (MEPIQ lowest absolute energy)

^b^ relative to G-E’ conformer (BEPIQ lowest absolute energy)

**Tab. 11S.** Atom coordinates of conformer A’.

| No | Symbol | X | Y | Z |
| --- | --- | --- | --- | --- |
| 1 | C | 0.0670360 | -0.1482020 | -0.0438560 |
| 2 | C | 0.0590560 | -0.1735290 | 1.3544310 |
| 3 | C | 1.2732820 | -0.0883400 | -0.7336900 |
| 4 | C | 1.2588820 | -0.1468920 | 2.0590000 |
| 5 | C | 2.4970590 | -0.0465710 | -0.0445550 |
| 6 | C | 2.4727760 | -0.0877460 | 1.3668880 |
| 7 | C | 3.7992050 | 0.0136970 | -0.6812740 |
| 8 | N | 4.9621770 | -0.0513730 | 0.1392760 |
| 9 | N | 3.6818530 | -0.0738520 | 2.0710980 |
| 10 | C | 4.9558920 | -0.0832030 | 1.5436440 |
| 11 | C | 4.2265010 | 0.0748140 | -1.9737390 |
| 12 | N | 5.6301360 | 0.0525140 | -1.9527260 |
| 13 | C | 6.1247490 | -0.0495230 | -0.6611560 |
| 14 | C | 3.4741680 | 0.1797330 | -3.2380830 |
| 15 | C | 2.7433370 | 1.3422890 | -3.5300790 |
| 16 | C | 3.4978150 | -0.8677200 | -4.1730780 |
| 17 | C | 2.0395710 | 1.4495810 | -4.7292490 |
| 18 | C | 2.8003370 | -0.7529690 | -5.3751130 |
| 19 | C | 2.0691180 | 0.4037130 | -5.6547130 |
| 20 | O | 7.2968080 | -0.1103420 | -0.3358480 |
| 21 | O | 5.9542890 | -0.1107800 | 2.2359540 |
| 22 | H | -0.8663790 | -0.1778750 | -0.5963830 |
| 23 | H | -0.8798700 | -0.2190400 | 1.8968380 |
| 24 | H | 1.2738020 | -0.0757120 | -1.8160660 |
| 25 | H | 1.2627610 | -0.1761950 | 3.1450120 |
| 26 | H | 2.7302250 | 2.1540940 | -2.8095760 |
| 27 | H | 4.0551690 | -1.7694810 | -3.9433920 |
| 28 | H | 1.4753530 | 2.3520160 | -4.9438940 |
| 29 | H | 2.8233590 | -1.5691690 | -6.0907590 |
| 30 | H | 1.5250960 | 0.4902570 | -6.5903660 |
| 31 | C | 6.8782570 | -1.3801750 | -3.5904060 |
| 32 | O | 7.6347300 | -1.5612570 | -4.5193690 |
| 33 | O | 6.2613760 | -2.3499210 | -2.9004220 |
| 34 | C | 6.5703620 | -3.7143230 | -3.2927670 |
| 35 | H | 7.6461840 | -3.8721370 | -3.1732530 |
| 36 | H | 6.3285560 | -3.8353810 | -4.3531030 |
| 37 | C | 5.7505550 | -4.6252920 | -2.4025650 |
| 38 | H | 5.9578750 | -5.6702760 | -2.6510350 |
| 39 | H | 5.9991920 | -4.4620310 | -1.3505220 |
| 40 | H | 4.6805790 | -4.4427370 | -2.5384510 |
| 41 | C | 6.5246280 | 0.0164880 | -3.0828580 |
| 42 | H | 7.4630190 | 0.4942670 | -2.7912910 |
| 43 | H | 6.0979100 | 0.5770380 | -3.9169550 |
| 44 | H | 3.6646890 | -0.1007690 | 3.0811910 |

**Tab. 12S.** Atom coordinates of conformer B’.

| No | Symbol | X | Y | Z |
| --- | --- | --- | --- | --- |
| 1 | C | 0.0666500 | 0.1470240 | -0.0352280 |
| 2 | C | 0.0610870 | 0.1666000 | 1.3631630 |
| 3 | C | 1.2716590 | 0.0889980 | -0.7273800 |
| 4 | C | 1.2621030 | 0.1361450 | 2.0655440 |
| 5 | C | 2.4965830 | 0.0434040 | -0.0405320 |
| 6 | C | 2.4747490 | 0.0788970 | 1.3710960 |
| 7 | C | 3.7975720 | -0.0155760 | -0.6797230 |
| 8 | N | 4.9620460 | 0.0449420 | 0.1390540 |
| 9 | N | 3.6850210 | 0.0613020 | 2.0731510 |
| 10 | C | 4.9581580 | 0.0717330 | 1.5435460 |
| 11 | C | 4.2225470 | -0.0721500 | -1.9731490 |
| 12 | N | 5.6262320 | -0.0515850 | -1.9545080 |
| 13 | C | 6.1232520 | 0.0445480 | -0.6633970 |
| 14 | C | 3.4678800 | -0.1714200 | -3.2365740 |
| 15 | C | 3.4906150 | 0.8797550 | -4.1674020 |
| 16 | C | 2.7356440 | -1.3322480 | -3.5319110 |
| 17 | C | 2.7908550 | 0.7703500 | -5.3686080 |
| 18 | C | 2.0296210 | -1.4342100 | -4.7302200 |
| 19 | C | 2.0582590 | -0.3846510 | -5.6515220 |
| 20 | O | 7.2959710 | 0.1021510 | -0.3398580 |
| 21 | O | 5.9577250 | 0.0962230 | 2.2342770 |
| 22 | H | -0.8676860 | 0.1797250 | -0.5860230 |
| 23 | H | -0.8768690 | 0.2106010 | 1.9073700 |
| 24 | H | 1.2703100 | 0.0807890 | -1.8097950 |
| 25 | H | 1.2679010 | 0.1609660 | 3.1516580 |
| 26 | H | 4.0490300 | 1.7801910 | -3.9351340 |
| 27 | H | 2.7232190 | -2.1469500 | -2.8146730 |
| 28 | H | 2.8131850 | 1.5894040 | -6.0810110 |
| 29 | H | 1.4643370 | -2.3353580 | -4.9474510 |
| 30 | H | 1.5124640 | -0.4670250 | -6.5865200 |
| 31 | C | 6.8717970 | 1.3851750 | -3.5906310 |
| 32 | O | 7.6251130 | 1.5684150 | -4.5217380 |
| 33 | O | 6.2585020 | 2.3533810 | -2.8953190 |
| 34 | C | 6.5673660 | 3.7186760 | -3.2846720 |
| 35 | H | 6.3223500 | 3.8429790 | -4.3438930 |
| 36 | H | 7.6436870 | 3.8752780 | -3.1680790 |
| 37 | C | 5.7511170 | 4.6277330 | -2.3892610 |
| 38 | H | 5.9585740 | 5.6732620 | -2.6353140 |
| 39 | H | 4.6805620 | 4.4465090 | -2.5223410 |
| 40 | H | 6.0028780 | 4.4611780 | -1.3384780 |
| 41 | C | 6.5186820 | -0.0127160 | -3.0861520 |
| 42 | H | 6.0904130 | -0.5709260 | -3.9210230 |
| 43 | H | 7.4574350 | -0.4915140 | -2.7973990 |
| 44 | H | 3.6696110 | 0.0847770 | 3.0833590 |

**Tab. 13S.** Atom coordinates of conformer C’

| No | Symbol | X | Y | Z |
| --- | --- | --- | --- | --- |
| 1 | C | 0.0718680 | -0.1768490 | 0.0008200 |
| 2 | C | 0.0485420 | -0.1771950 | 1.3993100 |
| 3 | C | 1.2843780 | -0.1015450 | -0.6761570 |
| 4 | C | 1.2379350 | -0.0921260 | 2.1166520 |
| 5 | C | 2.4993710 | -0.0306150 | 0.0260150 |
| 6 | C | 2.4579740 | -0.0134670 | 1.4373130 |
| 7 | C | 3.8070510 | 0.0445000 | -0.5973260 |
| 8 | N | 4.9485500 | 0.2271470 | 0.2353120 |
| 9 | N | 3.6550210 | 0.0901110 | 2.1541800 |
| 10 | C | 4.9253670 | 0.2460840 | 1.6402040 |
| 11 | C | 4.2518190 | 0.0324760 | -1.8844760 |
| 12 | N | 5.6416920 | 0.2379980 | -1.8538920 |
| 13 | C | 6.1169390 | 0.3193830 | -0.5506180 |
| 14 | C | 3.5490080 | -0.1978540 | -3.1580070 |
| 15 | C | 3.5577680 | 0.7725570 | -4.1715570 |
| 16 | C | 2.9095990 | -1.4262530 | -3.3933520 |
| 17 | C | 2.9386220 | 0.5190720 | -5.3954660 |
| 18 | C | 2.2832940 | -1.6731150 | -4.6134600 |
| 19 | C | 2.3004780 | -0.7024340 | -5.6186130 |
| 20 | O | 7.2777270 | 0.4360610 | -0.2017940 |
| 21 | O | 5.9075140 | 0.3788220 | 2.3430940 |
| 22 | H | -0.8545380 | -0.2323050 | -0.5613790 |
| 23 | H | -0.8950610 | -0.2380310 | 1.9319970 |
| 24 | H | 1.2968680 | -0.0950320 | -1.7586140 |
| 25 | H | 1.2290920 | -0.0818900 | 3.2028540 |
| 26 | H | 4.0422910 | 1.7276570 | -3.9924560 |
| 27 | H | 2.9151950 | -2.1797870 | -2.6127860 |
| 28 | H | 2.9521180 | 1.2769210 | -6.1725840 |
| 29 | H | 1.7916230 | -2.6260730 | -4.7838480 |
| 30 | H | 1.8205560 | -0.8986820 | -6.5724460 |
| 31 | C | 6.5121740 | -1.3116200 | -3.6113590 |
| 32 | O | 6.8842950 | -1.5163340 | -4.7463050 |
| 33 | O | 6.0465500 | -2.2474270 | -2.7732070 |
| 34 | C | 5.9067580 | -3.5835960 | -3.3209560 |
| 35 | H | 6.8814120 | -3.9188080 | -3.6872140 |
| 36 | H | 5.2232120 | -3.5313990 | -4.1743960 |
| 37 | C | 5.3731910 | -4.4637560 | -2.2096030 |
| 38 | H | 5.2480400 | -5.4874210 | -2.5745140 |
| 39 | H | 6.0632160 | -4.4786180 | -1.3616630 |
| 40 | H | 4.4033460 | -4.1001450 | -1.8579670 |
| 41 | C | 6.5500000 | 0.0709330 | -2.9657250 |
| 42 | H | 7.5565730 | 0.2440730 | -2.5735070 |
| 43 | H | 6.3609930 | 0.8001290 | -3.7552290 |
| 44 | H | 3.6240540 | 0.1158530 | 3.1640910 |

**Tab. 14S.** Atom coordinates of conformer D’.

| No | Symbol | X | Y | Z |
| --- | --- | --- | --- | --- |
| 1 | C | 0.0737610 | 0.1839760 | -0.0037630 |
| 2 | C | 0.0491450 | 0.1771100 | 1.3946880 |
| 3 | C | 1.2867060 | 0.1090810 | -0.6800020 |
| 4 | C | 1.2376590 | 0.0853770 | 2.1126680 |
| 5 | C | 2.5008620 | 0.0314510 | 0.0229080 |
| 6 | C | 2.4581380 | 0.0072340 | 1.4340570 |
| 7 | C | 3.8088940 | -0.0439950 | -0.5996430 |
| 8 | N | 4.9491620 | -0.2337430 | 0.2330910 |
| 9 | N | 3.6542760 | -0.1028720 | 2.1514770 |
| 10 | C | 4.9247110 | -0.2593760 | 1.6378670 |
| 11 | C | 4.2548160 | -0.0269800 | -1.8863410 |
| 12 | N | 5.6441000 | -0.2363860 | -1.8555390 |
| 13 | C | 6.1179580 | -0.3254990 | -0.5522760 |
| 14 | C | 3.5537710 | 0.2114280 | -3.1593720 |
| 15 | C | 2.9180930 | 1.4427800 | -3.3893660 |
| 16 | C | 3.5607670 | -0.7540510 | -4.1776240 |
| 17 | C | 2.2937390 | 1.6974000 | -4.6088770 |
| 18 | C | 2.9435750 | -0.4928250 | -5.4008960 |
| 19 | C | 2.3091640 | 0.7315800 | -5.6187330 |
| 20 | O | 7.2780930 | -0.4473190 | -0.2030380 |
| 21 | O | 5.9059300 | -0.3978120 | 2.3409580 |
| 22 | H | -0.8519810 | 0.2446920 | -0.5665150 |
| 23 | H | -0.8947980 | 0.2375160 | 1.9268240 |
| 24 | H | 1.3002140 | 0.1080850 | -1.7624630 |
| 25 | H | 1.2277820 | 0.0694970 | 3.1987900 |
| 26 | H | 2.9251010 | 2.1925380 | -2.6051780 |
| 27 | H | 4.0423380 | -1.7114140 | -4.0027010 |
| 28 | H | 1.8049960 | 2.6525950 | -4.7751070 |
| 29 | H | 2.9557070 | -1.2469070 | -6.1816900 |
| 30 | H | 1.8308020 | 0.9338600 | -6.5720900 |
| 31 | C | 6.5194250 | 1.3182070 | -3.6061810 |
| 32 | O | 6.8917850 | 1.5263100 | -4.7404310 |
| 33 | O | 6.0565170 | 2.2520280 | -2.7643110 |
| 34 | C | 5.9201280 | 3.5906340 | -3.3069370 |
| 35 | H | 5.2358360 | 3.5435100 | -4.1600760 |
| 36 | H | 6.8954630 | 3.9244770 | -3.6726300 |
| 37 | C | 5.3898010 | 4.4680980 | -2.1919070 |
| 38 | H | 5.2671540 | 5.4934550 | -2.5528960 |
| 39 | H | 4.4192520 | 4.1057950 | -1.8408710 |
| 40 | H | 6.0805110 | 4.4779200 | -1.3444490 |
| 41 | C | 6.5538480 | -0.0668830 | -2.9658400 |
| 42 | H | 6.3642470 | -0.7926830 | -3.7583340 |
| 43 | H | 7.5596670 | -0.2435280 | -2.5732370 |
| 44 | H | 3.6223640 | -0.1331100 | 3.1612370 |

**Tab. 15S.** Atom coordinates of conformer E’.

| No | Symbol | X | Y | Z |
| --- | --- | --- | --- | --- |
| 1 | C | 0.0117470 | 0.0379490 | -0.0372100 |
| 2 | C | -0.0022680 | 0.0438550 | 1.3611730 |
| 3 | C | 1.2216410 | 0.0191310 | -0.7229480 |
| 4 | C | 1.1953570 | 0.0411640 | 2.0698320 |
| 5 | C | 2.4439220 | 0.0002090 | -0.0300890 |
| 6 | C | 2.4128750 | 0.0252190 | 1.3817220 |
| 7 | C | 3.7500900 | -0.0204640 | -0.6619200 |
| 8 | N | 4.9064380 | 0.0776420 | 0.1651670 |
| 9 | N | 3.6188540 | 0.0389310 | 2.0913190 |
| 10 | C | 4.8936350 | 0.0975720 | 1.5696880 |
| 11 | C | 4.1856890 | -0.0617250 | -1.9531480 |
| 12 | N | 5.5873620 | 0.0103130 | -1.9231660 |
| 13 | C | 6.0715430 | 0.1180190 | -0.6284300 |
| 14 | C | 3.4429720 | -0.1858320 | -3.2206750 |
| 15 | C | 3.4718790 | 0.8493320 | -4.1696130 |
| 16 | C | 2.7136500 | -1.3529480 | -3.4993180 |
| 17 | C | 2.7775140 | 0.7163830 | -5.3719160 |
| 18 | C | 2.0136520 | -1.4769220 | -4.6989040 |
| 19 | C | 2.0460380 | -0.4433660 | -5.6381760 |
| 20 | O | 7.2410770 | 0.2066440 | -0.2989340 |
| 21 | O | 5.8880320 | 0.1521400 | 2.2662010 |
| 22 | H | -0.9199720 | 0.0511650 | -0.5932550 |
| 23 | H | -0.9440370 | 0.0564250 | 1.9004370 |
| 24 | H | 1.2257900 | 0.0221690 | -1.8052670 |
| 25 | H | 1.1945430 | 0.0561300 | 3.1561810 |
| 26 | H | 4.0330920 | 1.7517530 | -3.9517820 |
| 27 | H | 2.6977750 | -2.1545390 | -2.7674510 |
| 28 | H | 2.8013830 | 1.5235250 | -6.0979270 |
| 29 | H | 1.4498300 | -2.3821560 | -4.9028280 |
| 30 | H | 1.5035700 | -0.5418840 | -6.5736650 |
| 31 | C | 6.8331770 | 1.4642190 | -3.4700780 |
| 32 | O | 6.2228490 | 2.4552750 | -3.1305070 |
| 33 | O | 7.8836590 | 1.4544720 | -4.2997540 |
| 34 | C | 8.3064270 | 2.7472480 | -4.8146160 |
| 35 | H | 8.5124160 | 3.4067840 | -3.9667820 |
| 36 | H | 7.4788870 | 3.1814750 | -5.3845070 |
| 37 | C | 9.5332420 | 2.5089520 | -5.6705190 |
| 38 | H | 9.8863180 | 3.4595370 | -6.0810810 |
| 39 | H | 10.3388900 | 2.0673430 | -5.0777050 |
| 40 | H | 9.3042330 | 1.8364490 | -6.5019630 |
| 41 | C | 6.4987750 | 0.0381680 | -3.0370650 |
| 42 | H | 6.0647200 | -0.4935090 | -3.8875050 |
| 43 | H | 7.4230120 | -0.4662990 | -2.7476040 |
| 44 | H | 3.5965050 | 0.0641490 | 3.1013130 |

**Tab. 16S.** Atom coordinates of conformer F’.

| No | Symbol | X | Y | Z |
| --- | --- | --- | --- | --- |
| 1 | C | 0.0116780 | -0.0378330 | -0.0372490 |
| 2 | C | -0.0024330 | -0.0439950 | 1.3611310 |
| 3 | C | 1.2216180 | -0.0187530 | -0.7228990 |
| 4 | C | 1.1951420 | -0.0412900 | 2.0698740 |
| 5 | C | 2.4438490 | 0.0001890 | -0.0299510 |
| 6 | C | 2.4127060 | -0.0250710 | 1.3818530 |
| 7 | C | 3.7500590 | 0.0211120 | -0.6616880 |
| 8 | N | 4.9063570 | -0.0770480 | 0.1654610 |
| 9 | N | 3.6186390 | -0.0387540 | 2.0915310 |
| 10 | C | 4.8934620 | -0.0971710 | 1.5699790 |
| 11 | C | 4.1857440 | 0.0626440 | -1.9528800 |
| 12 | N | 5.5874210 | -0.0092880 | -1.9228120 |
| 13 | C | 6.0715180 | -0.1172030 | -0.6280620 |
| 14 | C | 3.4431080 | 0.1869120 | -3.2204360 |
| 15 | C | 2.7136660 | 1.3539960 | -3.4989040 |
| 16 | C | 3.4722110 | -0.8480620 | -4.1695760 |
| 17 | C | 2.0137420 | 1.4781210 | -4.6985170 |
| 18 | C | 2.7779190 | -0.7149590 | -5.3719050 |
| 19 | C | 2.0463230 | 0.4447540 | -5.6379900 |
| 20 | O | 7.2410350 | -0.2058230 | -0.2985050 |
| 21 | O | 5.8878190 | -0.1517120 | 2.2665520 |
| 22 | H | -0.9200000 | -0.0510590 | -0.5933620 |
| 23 | H | -0.9442380 | -0.0567780 | 1.9003280 |
| 24 | H | 1.2258430 | -0.0216000 | -1.8052180 |
| 25 | H | 1.1942520 | -0.0564600 | 3.1562210 |
| 26 | H | 2.6976420 | 2.1554400 | -2.7668800 |
| 27 | H | 4.0335180 | -1.7504570 | -3.9518810 |
| 28 | H | 1.4498270 | 2.3833280 | -4.9023050 |
| 29 | H | 2.8019400 | -1.5219550 | -6.0980730 |
| 30 | H | 1.5039140 | 0.5433910 | -6.5735010 |
| 31 | C | 6.8335640 | -1.4627940 | -3.4698340 |
| 32 | O | 6.2232610 | -2.4539700 | -3.1305670 |
| 33 | O | 7.8842190 | -1.4527970 | -4.2992860 |
| 34 | C | 8.3072230 | -2.7454370 | -4.8142950 |
| 35 | H | 7.4798450 | -3.1796440 | -5.3844350 |
| 36 | H | 8.5131030 | -3.4051050 | -3.9665360 |
| 37 | C | 9.5341910 | -2.5068620 | -5.6699000 |
| 38 | H | 9.8874510 | -3.4573380 | -6.0805580 |
| 39 | H | 9.3052850 | -1.8342350 | -6.5012720 |
| 40 | H | 10.3396700 | -2.0652750 | -5.0768410 |
| 41 | C | 6.4989230 | -0.0368520 | -3.0366450 |
| 42 | H | 7.4230650 | 0.4677130 | -2.7470520 |
| 43 | H | 6.0648460 | 0.4948760 | -3.8870420 |
| 44 | H | 3.5962250 | -0.0640950 | 3.1015210 |

**Tab. 17S.** Atom coordinates of conformer G’.

| No | Symbol | X | Y | Z |
| --- | --- | --- | --- | --- |
| 1 | C | 0.0121290 | -0.0362350 | -0.0359080 |
| 2 | C | -0.0009760 | -0.0439810 | 1.3624750 |
| 3 | C | 1.2215840 | -0.0174400 | -0.7224210 |
| 4 | C | 1.1971180 | -0.0431700 | 2.0703440 |
| 5 | C | 2.4443380 | -0.0004060 | -0.0303460 |
| 6 | C | 2.4141910 | -0.0273030 | 1.3814480 |
| 7 | C | 3.7501090 | 0.0201500 | -0.6630000 |
| 8 | N | 4.9069350 | -0.0798620 | 0.1631980 |
| 9 | N | 3.6206180 | -0.0429620 | 2.0902360 |
| 10 | C | 4.8950180 | -0.1017920 | 1.5677020 |
| 11 | C | 4.1848870 | 0.0628170 | -1.9544610 |
| 12 | N | 5.5865270 | -0.0102400 | -1.9254990 |
| 13 | C | 6.0714850 | -0.1200220 | -0.6312310 |
| 14 | C | 3.4414370 | 0.1889600 | -3.2213560 |
| 15 | C | 2.7126140 | 1.3568350 | -3.4981110 |
| 16 | C | 3.4691890 | -0.8450440 | -4.1715920 |
| 17 | C | 2.0119630 | 1.4826810 | -4.6971210 |
| 18 | C | 2.7741760 | -0.7102220 | -5.3733110 |
| 19 | C | 2.0431970 | 0.4502710 | -5.6376910 |
| 20 | O | 7.2411820 | -0.2099300 | -0.3026590 |
| 21 | O | 5.8898100 | -0.1580910 | 2.2635140 |
| 22 | H | -0.9199660 | -0.0479770 | -0.5913560 |
| 23 | H | -0.9424000 | -0.0565090 | 1.9023420 |
| 24 | H | 1.2250120 | -0.0190130 | -1.8047460 |
| 25 | H | 1.1970300 | -0.0595200 | 3.1566750 |
| 26 | H | 2.6976360 | 2.1575240 | -2.7652390 |
| 27 | H | 4.0300400 | -1.7480360 | -3.9552050 |
| 28 | H | 1.4485290 | 2.3884840 | -4.8995880 |
| 29 | H | 2.7971530 | -1.5164800 | -6.1003320 |
| 30 | H | 1.5002160 | 0.5502520 | -6.5727270 |
| 31 | C | 6.8306380 | -1.4628490 | -3.4750010 |
| 32 | O | 6.2200300 | -2.4540300 | -3.1363000 |
| 33 | O | 7.8806050 | -1.4525720 | -4.3053240 |
| 34 | C | 8.3024150 | -2.7448920 | -4.8221020 |
| 35 | H | 7.4742250 | -3.1781030 | -5.3918200 |
| 36 | H | 8.5088570 | -3.4055340 | -3.9752370 |
| 37 | C | 9.5286100 | -2.5060510 | -5.6787440 |
| 38 | H | 9.8809860 | -3.4562850 | -6.0907190 |
| 39 | H | 9.2991620 | -1.8324830 | -6.5092030 |
| 40 | H | 10.3349170 | -2.0654440 | -5.0860810 |
| 41 | C | 6.4972090 | -0.0371730 | -3.0400190 |
| 42 | H | 7.4218860 | 0.4664900 | -2.7505590 |
| 43 | H | 6.0628520 | 0.4957450 | -3.8895270 |
| 44 | H | 3.5988880 | -0.0696720 | 3.1002050 |

**Tab. 18S.** Atom coordinates of conformer H’.

| No | Symbol | X | Y | Z |
| --- | --- | --- | --- | --- |
| 1 | C | 0.0120690 | 0.0358480 | -0.0353090 |
| 2 | C | -0.0006810 | 0.0428980 | 1.3630820 |
| 3 | C | 1.2213530 | 0.0176740 | -0.7221410 |
| 4 | C | 1.1975930 | 0.0420280 | 2.0706440 |
| 5 | C | 2.4442870 | 0.0005940 | -0.0303860 |
| 6 | C | 2.4144920 | 0.0268020 | 1.3814270 |
| 7 | C | 3.7499010 | -0.0193760 | -0.6633810 |
| 8 | N | 4.9069260 | 0.0803820 | 0.1625680 |
| 9 | N | 3.6210950 | 0.0424340 | 2.0899140 |
| 10 | C | 4.8953500 | 0.1017720 | 1.5670830 |
| 11 | C | 4.1843490 | -0.0613150 | -1.9549730 |
| 12 | N | 5.5859870 | 0.0119110 | -1.9263410 |
| 13 | C | 6.0712720 | 0.1210660 | -0.6321400 |
| 14 | C | 3.4405820 | -0.1869530 | -3.2217370 |
| 15 | C | 3.4677450 | 0.8476030 | -4.1713850 |
| 16 | C | 2.7120400 | -1.3548930 | -3.4989590 |
| 17 | C | 2.7724370 | 0.7132580 | -5.3729870 |
| 18 | C | 2.0110940 | -1.4802690 | -4.6978470 |
| 19 | C | 2.0417440 | -0.4473090 | -5.6378310 |
| 20 | O | 7.2410440 | 0.2108910 | -0.3038130 |
| 21 | O | 5.8902980 | 0.1580530 | 2.2626730 |
| 22 | H | -0.9201700 | 0.0476410 | -0.5905130 |
| 23 | H | -0.9419710 | 0.0549260 | 1.9031940 |
| 24 | H | 1.2245050 | 0.0197710 | -1.8044650 |
| 25 | H | 1.1977870 | 0.0578270 | 3.1569830 |
| 26 | H | 4.0283690 | 1.7506480 | -3.9546360 |
| 27 | H | 2.6975080 | -2.1560080 | -2.7665430 |
| 28 | H | 2.7949600 | 1.5199420 | -6.0995500 |
| 29 | H | 1.4478800 | -2.3861290 | -4.9006750 |
| 30 | H | 1.4985310 | -0.5469210 | -6.5727710 |
| 31 | C | 6.8290840 | 1.4656280 | -3.4756200 |
| 32 | O | 6.2185040 | 2.4564900 | -3.1359380 |
| 33 | O | 7.8784230 | 1.4560290 | -4.3067460 |
| 34 | C | 8.2995380 | 2.7487250 | -4.8231530 |
| 35 | H | 8.5064570 | 3.4089680 | -3.9760940 |
| 36 | H | 7.4708150 | 3.1820390 | -5.3920180 |
| 37 | C | 9.5251460 | 2.5106360 | -5.6808430 |
| 38 | H | 9.8769730 | 3.4611740 | -6.0925880 |
| 39 | H | 10.3320100 | 2.0699220 | -5.0890190 |
| 40 | H | 9.2952420 | 1.8374440 | -6.5114820 |
| 41 | C | 6.4963370 | 0.0396490 | -3.0411080 |
| 42 | H | 6.0619820 | -0.4931250 | -3.8907070 |
| 43 | H | 7.4212960 | -0.4637460 | -2.7520800 |
| 44 | H | 3.5996090 | 0.0687870 | 3.0998970 |

**Tab. 19S.** Atom coordinates of conformer A-F’.

| No | Symbol | X | Y | Z |
| --- | --- | --- | --- | --- |
| 1 | C | 0.0780600 | -0.1251170 | -0.0351160 |
| 2 | C | 0.0596510 | -0.1149290 | 1.3618940 |
| 3 | C | 1.2961820 | -0.0728190 | -0.7051450 |
| 4 | C | 1.2524530 | -0.0558020 | 2.0819490 |
| 5 | C | 2.5099300 | -0.0014530 | -0.0002140 |
| 6 | C | 2.4861330 | 0.0035620 | 1.4144980 |
| 7 | C | 3.8015950 | 0.0399540 | -0.6513420 |
| 8 | N | 4.9646970 | -0.0166290 | 0.1611150 |
| 9 | C | 4.9677030 | -0.0121310 | 1.5601180 |
| 10 | C | 4.2153210 | 0.0679400 | -1.9481160 |
| 11 | N | 5.6191060 | 0.0274120 | -1.9386170 |
| 12 | C | 6.1229610 | -0.0420710 | -0.6505300 |
| 13 | C | 3.4526970 | 0.1492180 | -3.2099660 |
| 14 | C | 2.8203220 | 1.3491400 | -3.5745200 |
| 15 | C | 3.3626060 | -0.9638260 | -4.0621010 |
| 16 | C | 2.0994790 | 1.4319300 | -4.7678820 |
| 17 | C | 2.6447600 | -0.8757390 | -5.2570990 |
| 18 | C | 2.0114770 | 0.3198520 | -5.6115050 |
| 19 | O | 7.3011240 | -0.0933410 | -0.3331710 |
| 20 | O | 5.9935320 | -0.0500130 | 2.2179830 |
| 21 | H | -0.8474980 | -0.1804030 | -0.5989000 |
| 22 | H | -0.8815450 | -0.1605630 | 1.9005840 |
| 23 | H | 1.3152690 | -0.0937000 | -1.7873840 |
| 24 | H | 1.2023560 | -0.0624590 | 3.1629090 |
| 25 | H | 2.8922010 | 2.2094210 | -2.9156630 |
| 26 | H | 3.8502090 | -1.8905530 | -3.7780900 |
| 27 | H | 1.6109020 | 2.3629940 | -5.0392300 |
| 28 | H | 2.5752540 | -1.7427400 | -5.9073930 |
| 29 | H | 1.4513240 | 0.3847190 | -6.5396300 |
| 30 | C | 6.7477480 | -1.4355430 | -3.5738480 |
| 31 | O | 6.2125670 | -2.4320160 | -3.1332710 |
| 32 | O | 7.6374080 | -1.4202890 | -4.5744060 |
| 33 | C | 7.9894000 | -2.7089320 | -5.1588570 |
| 34 | H | 7.0769080 | -3.1672300 | -5.5518380 |
| 35 | H | 8.3794630 | -3.3496070 | -4.3628590 |
| 36 | C | 9.0151910 | -2.4467650 | -6.2425830 |
| 37 | H | 9.3061520 | -3.3948900 | -6.7058980 |
| 38 | H | 8.6059150 | -1.7935940 | -7.0190700 |
| 39 | H | 9.9106560 | -1.9765290 | -5.8258840 |
| 40 | C | 6.4943720 | -0.0123150 | -3.0816440 |
| 41 | H | 7.4515740 | 0.4349930 | -2.8026540 |
| 42 | H | 6.0682410 | 0.5729740 | -3.9006700 |
| 43 | N | 3.7020340 | 0.0615410 | 2.1382360 |
| 44 | C | 3.6735150 | 0.0241940 | 3.5941030 |
| 45 | C | 3.3105590 | -1.3335680 | 4.1880900 |
| 46 | H | 2.9830770 | 0.7712480 | 3.9887950 |
| 47 | H | 4.6741710 | 0.2725230 | 3.9518980 |
| 48 | O | 2.8944450 | -1.4614140 | 5.3243310 |
| 49 | O | 3.5323390 | -2.3353240 | 3.3342490 |
| 50 | C | 3.2472810 | -3.6837290 | 3.8039460 |
| 51 | H | 2.2056710 | -3.7173230 | 4.1369860 |
| 52 | H | 3.8902440 | -3.8888930 | 4.6649640 |
| 53 | C | 3.5150490 | -4.6226060 | 2.6451830 |
| 54 | H | 3.3176070 | -5.6534310 | 2.9562590 |
| 55 | H | 2.8672890 | -4.3862640 | 1.7957670 |
| 56 | H | 4.5568480 | -4.5515690 | 2.3196600 |

**Tab. 20S.** Atom coordinates of conformer B-E’.

| No | Symbol | X | Y | Z |
| --- | --- | --- | --- | --- |
| 1 | C | 0.0591460 | 0.0971470 | -0.053057 |
| 2 | C | 0.0336990 | 0.0899310 | 1.3438570 |
| 3 | C | 1.2810080 | 0.0514570 | -0.716728 |
| 4 | C | 1.2231630 | 0.0402210 | 2.0701320 |
| 5 | C | 2.4915560 | -0.0102840 | -0.005440 |
| 6 | C | 2.4606210 | -0.0124660 | 1.4091480 |
| 7 | C | 3.7867590 | -0.0440000 | -0.649940 |
| 8 | N | 4.9452110 | 0.0217890 | 0.1683990 |
| 9 | C | 4.9412630 | 0.0195620 | 1.5673730 |
| 10 | C | 4.2073710 | -0.0707660 | -1.944526 |
| 11 | N | 5.6107890 | -0.0202220 | -1.927816 |
| 12 | C | 6.1074010 | 0.0545640 | -0.637215 |
| 13 | C | 3.4519460 | -0.1589930 | -3.210252 |
| 14 | C | 3.3573770 | 0.9527350 | -4.063609 |
| 15 | C | 2.8311630 | -1.3641990 | -3.577299 |
| 16 | C | 2.6466520 | 0.8580950 | -5.262361 |
| 17 | C | 2.1173850 | -1.4535610 | -4.774439 |
| 18 | C | 2.0249690 | -0.3428050 | -5.619329 |
| 19 | O | 7.2834240 | 0.1148820 | -0.313633 |
| 20 | O | 5.9636260 | 0.0648500 | 2.2301580 |
| 21 | H | -0.8638720 | 0.1450310 | -0.621664 |
| 22 | H | -0.9105160 | 0.1305260 | 1.8776450 |
| 23 | H | 1.3055860 | 0.0701540 | -1.798903 |
| 24 | H | 1.1674170 | 0.0489530 | 3.1508130 |
| 25 | H | 3.8359830 | 1.8835310 | -3.777631 |
| 26 | H | 2.9064850 | -2.2234800 | -2.917521 |
| 27 | H | 2.5736300 | 1.7240940 | -5.913602 |
| 28 | H | 1.6377700 | -2.3887100 | -5.047729 |
| 29 | H | 1.4703620 | -0.4128070 | -6.550405 |
| 30 | C | 6.7361990 | 1.4495820 | -3.559131 |
| 31 | O | 6.1913330 | 2.4424230 | -3.122243 |
| 32 | O | 7.6305980 | 1.4400150 | -4.555530 |
| 33 | C | 7.9756640 | 2.7306700 | -5.139652 |
| 34 | H | 8.3591610 | 3.3743060 | -4.342843 |
| 35 | H | 7.0613110 | 3.1827040 | -5.535526 |
| 36 | C | 9.0063600 | 2.4747820 | -6.220225 |
| 37 | H | 9.2924840 | 3.4246020 | -6.683080 |
| 38 | H | 9.9036500 | 2.0105800 | -5.800691 |
| 39 | H | 8.6036320 | 1.8186320 | -6.997621 |
| 40 | C | 6.4916930 | 0.0250140 | -3.066291 |
| 41 | H | 6.0749050 | -0.5649880 | -3.886735 |
| 42 | H | 7.4510020 | -0.4140300 | -2.781472 |
| 43 | N | 3.6731610 | -0.0608850 | 2.1392370 |
| 44 | C | 3.6369050 | -0.0200550 | 3.5948360 |
| 45 | C | 3.2616210 | 1.3367720 | 4.1833080 |
| 46 | H | 4.6373900 | -0.2605790 | 3.9583730 |
| 47 | H | 2.9495740 | -0.7708060 | 3.9879410 |
| 48 | O | 2.8383570 | 1.4647930 | 5.3168870 |
| 49 | O | 3.4816090 | 2.3377780 | 3.3281280 |
| 50 | C | 3.1851980 | 3.6854990 | 3.7927290 |
| 51 | H | 3.8216720 | 3.8968760 | 4.6570600 |
| 52 | H | 2.1414140 | 3.7133130 | 4.1194420 |
| 53 | C | 3.4539340 | 4.6231880 | 2.6332230 |
| 54 | H | 3.2480570 | 5.6534990 | 2.9405120 |
| 55 | H | 4.4981020 | 4.5580140 | 2.3141240 |
| 56 | H | 2.8128100 | 4.3805770 | 1.7805460 |

**Tab. 21S.** Atom coordinates of conformer C-E’.

| No | Symbol | X | Y | Z |
| --- | --- | --- | --- | --- |
| 1 | C | 0.1635920 | -0.0778630 | -0.052470 |
| 2 | C | 0.1502100 | -0.0955630 | 1.3445320 |
| 3 | C | 1.3805150 | -0.0482150 | -0.726118 |
| 4 | C | 1.3463090 | -0.0714590 | 2.0611930 |
| 5 | C | 2.5982020 | -0.0355220 | -0.024520 |
| 6 | C | 2.5788670 | -0.0316600 | 1.3902120 |
| 7 | C | 3.8877370 | -0.0195850 | -0.680247 |
| 8 | N | 5.0530540 | 0.0399320 | 0.1289340 |
| 9 | C | 5.0614680 | 0.0201820 | 1.5275830 |
| 10 | C | 4.2972980 | -0.0289360 | -1.977952 |
| 11 | N | 5.7007020 | 0.0219260 | -1.973160 |
| 12 | C | 6.2086130 | 0.0840500 | -0.686133 |
| 13 | C | 3.5334770 | -0.1089960 | -3.240299 |
| 14 | C | 3.3599070 | 1.0308540 | -4.041874 |
| 15 | C | 2.9877460 | -1.3343220 | -3.656330 |
| 16 | C | 2.6445190 | 0.9440550 | -5.238628 |
| 17 | C | 2.2698500 | -1.4165870 | -4.851600 |
| 18 | C | 2.0980040 | -0.2775090 | -5.644819 |
| 19 | O | 7.3867110 | 0.1495520 | -0.371166 |
| 20 | O | 6.0915430 | 0.0183440 | 2.1801890 |
| 21 | H | -0.7652440 | -0.0883500 | -0.613469 |
| 22 | H | -0.7900750 | -0.1259130 | 1.8858700 |
| 23 | H | 1.3956710 | -0.0338960 | -1.808574 |
| 24 | H | 1.2996710 | -0.0838710 | 3.1422560 |
| 25 | H | 3.7847340 | 1.9756690 | -3.719505 |
| 26 | H | 3.1230540 | -2.2149590 | -3.035326 |
| 27 | H | 2.5103100 | 1.8314450 | -5.850103 |
| 28 | H | 1.8477970 | -2.3674470 | -5.163043 |
| 29 | H | 1.5396290 | -0.3416840 | -6.574075 |
| 30 | C | 6.7454600 | 1.5425820 | -3.610645 |
| 31 | O | 6.1876780 | 2.5140520 | -3.143304 |
| 32 | O | 7.6012160 | 1.5693370 | -4.640213 |
| 33 | C | 7.8842520 | 2.8753150 | -5.223673 |
| 34 | H | 8.2760970 | 3.5240210 | -4.435082 |
| 35 | H | 6.9422510 | 3.3013430 | -5.581700 |
| 36 | C | 8.8821800 | 2.6618120 | -6.343485 |
| 37 | H | 9.1205920 | 3.6242050 | -6.807404 |
| 38 | H | 9.8087280 | 2.2233730 | -5.961466 |
| 39 | H | 8.4723000 | 1.9995010 | -7.111867 |
| 40 | C | 6.5650400 | 0.1071530 | -3.121759 |
| 41 | H | 6.1644330 | -0.4968530 | -3.940113 |
| 42 | H | 7.5442780 | -0.2934350 | -2.848201 |
| 43 | N | 3.7967450 | 0.0105130 | 2.1114430 |
| 44 | C | 3.7733770 | -0.0762210 | 3.5652660 |
| 45 | C | 3.3885330 | -1.4475590 | 4.1101210 |
| 46 | H | 3.0992370 | 0.6680620 | 3.9924090 |
| 47 | H | 4.7806800 | 0.1421580 | 3.9245550 |
| 48 | O | 2.9469060 | -1.6079220 | 5.2322910 |
| 49 | O | 3.6248360 | -2.4266800 | 3.2322980 |
| 50 | C | 3.3170510 | -3.7844770 | 3.6576980 |
| 51 | H | 2.2660330 | -3.8191570 | 3.9594610 |
| 52 | H | 3.9330570 | -4.0183670 | 4.5310010 |
| 53 | C | 3.6110190 | -4.6950060 | 2.4826420 |
| 54 | H | 3.3954340 | -5.7318370 | 2.7597260 |
| 55 | H | 2.9904000 | -4.4298710 | 1.6214160 |
| 56 | H | 4.6629470 | -4.6257060 | 2.1905970 |

**Tab. 22S.** Atom coordinates of conformer D-F’.

| No | Symbol | X | Y | Z |
| --- | --- | --- | --- | --- |
| 1 | C | 0.1578340 | 0.0701900 | -0.052650 |
| 2 | C | 0.1446500 | 0.0880670 | 1.3443510 |
| 3 | C | 1.3747010 | 0.0428980 | -0.726500 |
| 4 | C | 1.3409160 | 0.0665030 | 2.0608100 |
| 5 | C | 2.5925310 | 0.0327740 | -0.025107 |
| 6 | C | 2.5734390 | 0.0291210 | 1.3896270 |
| 7 | C | 3.8819900 | 0.0193290 | -0.681043 |
| 8 | N | 5.0475600 | -0.0378290 | 0.1279510 |
| 9 | C | 5.0561660 | -0.0177340 | 1.5266000 |
| 10 | C | 4.2913140 | 0.0294170 | -1.978819 |
| 11 | N | 5.6948150 | -0.0186200 | -1.974258 |
| 12 | C | 6.2030650 | -0.0797020 | -0.687317 |
| 13 | C | 3.5271170 | 0.1079150 | -3.241033 |
| 14 | C | 2.9786930 | 1.3321000 | -3.656887 |
| 15 | C | 3.3558390 | -1.0322520 | -4.042654 |
| 16 | C | 2.2604130 | 1.4129200 | -4.852024 |
| 17 | C | 2.6400500 | -0.9468930 | -5.239270 |
| 18 | C | 2.0908600 | 0.2735300 | -5.645287 |
| 19 | O | 7.3813490 | -0.1428950 | -0.372572 |
| 20 | O | 6.0863380 | -0.0137120 | 2.1790450 |
| 21 | H | -0.7711150 | 0.0786990 | -0.613494 |
| 22 | H | -0.7956040 | 0.1165690 | 1.8858420 |
| 23 | H | 1.3896940 | 0.0284450 | -1.808956 |
| 24 | H | 1.2944450 | 0.0789820 | 3.1418800 |
| 25 | H | 3.1122390 | 2.2129800 | -3.035846 |
| 26 | H | 3.7827050 | -1.9761960 | -3.720424 |
| 27 | H | 1.8362850 | 2.3629010 | -5.163331 |
| 28 | H | 2.5076080 | -1.8345270 | -5.850778 |
| 29 | H | 1.5321850 | 0.3365710 | -6.574441 |
| 30 | C | 6.7425500 | -1.5372770 | -3.611677 |
| 31 | O | 6.1867260 | -2.5098210 | -3.144239 |
| 32 | O | 7.5984240 | -1.5624200 | -4.641188 |
| 33 | C | 7.8842110 | -2.8679000 | -5.224422 |
| 34 | H | 6.9431550 | -3.2958330 | -5.582663 |
| 35 | H | 8.2771150 | -3.5157380 | -4.435646 |
| 36 | C | 8.8820310 | -2.6525410 | -6.343975 |
| 37 | H | 9.1224790 | -3.6145080 | -6.807727 |
| 38 | H | 8.4710650 | -1.9911220 | -7.112546 |
| 39 | H | 9.8075990 | -2.2122290 | -5.961737 |
| 40 | C | 6.5591510 | -0.1021630 | -3.122987 |
| 41 | H | 7.5375760 | 0.3005670 | -2.849662 |
| 42 | H | 6.1571020 | 0.5008590 | -3.941361 |
| 43 | N | 3.7915210 | -0.0103980 | 2.1106660 |
| 44 | C | 3.7682260 | 0.0769380 | 3.5644570 |
| 45 | C | 3.3806700 | 1.4477400 | 4.1087370 |
| 46 | H | 4.7760390 | -0.1392030 | 3.9236590 |
| 47 | H | 3.0957030 | -0.6685260 | 3.9920810 |
| 48 | O | 2.9388700 | 1.6077250 | 5.2308920 |
| 49 | O | 3.6148960 | 2.4269310 | 3.2304350 |
| 50 | C | 3.3044430 | 3.7843100 | 3.6552350 |
| 51 | H | 3.9203220 | 4.0199490 | 4.5281590 |
| 52 | H | 2.2534810 | 3.8169650 | 3.9574170 |
| 53 | C | 3.5960310 | 4.6948210 | 2.4795740 |
| 54 | H | 3.3784080 | 5.7313500 | 2.7561960 |
| 55 | H | 4.6479790 | 4.6275550 | 2.1871210 |
| 56 | H | 2.9756050 | 4.4279330 | 1.6187500 |

**Tab. 23S.** Atom coordinates of conformer E-E’.

| No | Symbol | X | Y | Z |
| --- | --- | --- | --- | --- |
| 1 | C | 0.1131230 | 0.1694450 | 0.0270730 |
| 2 | C | 0.1060860 | 0.1549410 | 1.4242280 |
| 3 | C | 1.3255680 | 0.1187410 | -0.6529020 |
| 4 | C | 1.3044790 | 0.0917370 | 2.1338090 |
| 5 | C | 2.5446790 | 0.0414340 | 0.0420290 |
| 6 | C | 2.5322050 | 0.0301550 | 1.4567280 |
| 7 | C | 3.8310020 | -0.0002270 | -0.6198320 |
| 8 | N | 5.0012310 | 0.0585400 | 0.1822250 |
| 9 | C | 5.0156900 | 0.0404230 | 1.5809050 |
| 10 | C | 4.2334170 | -0.0303920 | -1.9200390 |
| 11 | N | 5.6372300 | 0.0114340 | -1.9230260 |
| 12 | C | 6.1524660 | 0.0802630 | -0.6392960 |
| 13 | C | 3.4595430 | -0.1140640 | -3.1748400 |
| 14 | C | 3.3622280 | 0.9968880 | -4.0288260 |
| 15 | C | 2.8228130 | -1.3143890 | -3.5304740 |
| 16 | C | 2.6332950 | 0.9062280 | -5.2169110 |
| 17 | C | 2.0908200 | -1.3997380 | -4.7168350 |
| 18 | C | 1.9958500 | -0.2897430 | -5.5624730 |
| 19 | O | 7.3336620 | 0.1247440 | -0.3320370 |
| 20 | O | 6.0456700 | 0.0675640 | 2.2327290 |
| 21 | H | -0.8168540 | 0.2297570 | -0.5288640 |
| 22 | H | -0.8307180 | 0.2042970 | 1.9702340 |
| 23 | H | 1.3361280 | 0.1455490 | -1.7351120 |
| 24 | H | 1.2650750 | 0.1035420 | 3.2150350 |
| 25 | H | 3.8526020 | 1.9240620 | -3.7513260 |
| 26 | H | 2.8999610 | -2.1728910 | -2.8698670 |
| 27 | H | 2.5580840 | 1.7718560 | -5.8683940 |
| 28 | H | 1.5988490 | -2.3310760 | -4.9811460 |
| 29 | H | 1.4268510 | -0.3564690 | -6.4850910 |
| 30 | C | 6.7537370 | 1.4497990 | -3.5899920 |
| 31 | O | 6.2263710 | 2.4543500 | -3.1593710 |
| 32 | O | 7.6327230 | 1.4165360 | -4.6002440 |
| 33 | C | 7.9813630 | 2.6953000 | -5.2075630 |
| 34 | H | 8.3790480 | 3.3478980 | -4.4251530 |
| 35 | H | 7.0662210 | 3.1489680 | -5.5998140 |
| 36 | C | 8.9973340 | 2.4148490 | -6.2960050 |
| 37 | H | 9.2854110 | 3.3551930 | -6.7766770 |
| 38 | H | 9.8958630 | 1.9499670 | -5.8798320 |
| 39 | H | 8.5805500 | 1.7501070 | -7.0586040 |
| 40 | C | 6.5025010 | 0.0347130 | -3.0740580 |
| 41 | H | 6.0690100 | -0.5619190 | -3.8810460 |
| 42 | H | 7.4616240 | -0.4095770 | -2.7966320 |
| 43 | N | 3.7547090 | -0.0440660 | 2.1680330 |
| 44 | C | 3.7467240 | -0.0774500 | 3.6203890 |
| 45 | C | 3.4789710 | 1.2927630 | 4.2359390 |
| 46 | H | 4.7327100 | -0.4007560 | 3.9578880 |
| 47 | H | 3.0194120 | -0.8050700 | 3.9861600 |
| 48 | O | 3.4964800 | 2.3440160 | 3.6322920 |
| 49 | O | 3.2405470 | 1.1644520 | 5.5508080 |
| 50 | C | 3.0307920 | 2.3975600 | 6.2993340 |
| 51 | H | 2.1765540 | 2.9230180 | 5.8622090 |
| 52 | H | 3.9173970 | 3.0266400 | 6.1778850 |
| 53 | C | 2.7918950 | 2.0121530 | 7.7451180 |
| 54 | H | 2.6320850 | 2.9158080 | 8.3419220 |
| 55 | H | 1.9072680 | 1.3751080 | 7.8394130 |
| 56 | H | 3.6537900 | 1.4763200 | 8.1535710 |

**Tab. 24S.** Atom coordinates of conformer F-F’.

| No | Symbol | X | Y | Z |
| --- | --- | --- | --- | --- |
| 1 | C | 0.1738040 | -0.1967640 | 0.0988820 |
| 2 | C | 0.1929770 | -0.1811390 | 1.4959260 |
| 3 | C | 1.3727830 | -0.1385770 | -0.6039860 |
| 4 | C | 1.4041370 | -0.1090280 | 2.1825490 |
| 5 | C | 2.6043360 | -0.0519770 | 0.0675980 |
| 6 | C | 2.6182850 | -0.0390640 | 1.4822650 |
| 7 | C | 3.8778370 | -0.0036690 | -0.6183200 |
| 8 | N | 5.0631960 | -0.0584700 | 0.1615630 |
| 9 | C | 5.1034750 | -0.0371770 | 1.5597470 |
| 10 | C | 4.2555860 | 0.0274910 | -1.9259470 |
| 11 | N | 5.6592480 | -0.0104760 | -1.9554230 |
| 12 | C | 6.1988750 | -0.0772180 | -0.6816130 |
| 13 | C | 3.4576760 | 0.1089040 | -3.1656340 |
| 14 | C | 2.8014380 | 1.3039890 | -3.5030900 |
| 15 | C | 3.3562300 | -0.9986660 | -4.0235590 |
| 16 | C | 2.0461030 | 1.3871970 | -4.6748380 |
| 17 | C | 2.6040740 | -0.9100460 | -5.1972240 |
| 18 | C | 1.9471090 | 0.2805270 | -5.5243800 |
| 19 | O | 7.3858980 | -0.1179920 | -0.3970130 |
| 20 | O | 6.1451880 | -0.0594550 | 2.1927460 |
| 21 | H | -0.7660420 | -0.2641660 | -0.4393670 |
| 22 | H | -0.7329590 | -0.2368460 | 2.0595810 |
| 23 | H | 1.3628990 | -0.1669080 | -1.6861460 |
| 24 | H | 1.3856920 | -0.1211300 | 3.2642680 |
| 25 | H | 2.8817870 | 2.1599820 | -2.8396240 |
| 26 | H | 3.8611450 | -1.9220980 | -3.7599930 |
| 27 | H | 1.5391090 | 2.3144110 | -4.9248830 |
| 28 | H | 2.5259640 | -1.7732050 | -5.8516330 |
| 29 | H | 1.3599540 | 0.3456720 | -6.4356620 |
| 30 | C | 6.7540210 | -1.4457860 | -3.6394010 |
| 31 | O | 6.2387470 | -2.4523340 | -3.1988650 |
| 32 | O | 7.6165900 | -1.4096700 | -4.6635790 |
| 33 | C | 7.9617060 | -2.6875370 | -5.2748270 |
| 34 | H | 7.0428750 | -3.1451970 | -5.6536080 |
| 35 | H | 8.3731230 | -3.3379030 | -4.4976970 |
| 36 | C | 8.9607970 | -2.4039100 | -6.3779740 |
| 37 | H | 9.2454600 | -3.3434570 | -6.8622240 |
| 38 | H | 8.5305810 | -1.7412140 | -7.1348770 |
| 39 | H | 9.8634580 | -1.9353870 | -5.9750470 |
| 40 | C | 6.5030200 | -0.0310030 | -3.1223700 |
| 41 | H | 7.4641950 | 0.4206740 | -2.8646510 |
| 42 | H | 6.0498230 | 0.5600720 | -3.9226000 |
| 43 | N | 3.8534890 | 0.0449650 | 2.1701340 |
| 44 | C | 3.8736710 | 0.0828830 | 3.6223110 |
| 45 | C | 3.6371590 | -1.2888260 | 4.2472870 |
| 46 | H | 3.1433170 | 0.8012860 | 3.9999160 |
| 47 | H | 4.8610470 | 0.4215000 | 3.9399810 |
| 48 | O | 3.6436550 | -2.3406030 | 3.6443610 |
| 49 | O | 3.4400970 | -1.1608900 | 5.5690120 |
| 50 | C | 3.2647060 | -2.3949090 | 6.3248960 |
| 51 | H | 4.1505830 | -3.0182430 | 6.1726920 |
| 52 | H | 2.3992330 | -2.9262410 | 5.9180220 |
| 53 | C | 3.0740890 | -2.0102530 | 7.7780290 |
| 54 | H | 2.9398310 | -2.9145090 | 8.3801970 |
| 55 | H | 3.9468080 | -1.4697320 | 8.1562010 |
| 56 | H | 2.1899310 | -1.3778380 | 7.9028540 |

**Tab. 25S.** Atom coordinates of conformer G-E’.

| No | Symbol | X | Y | Z |
| --- | --- | --- | --- | --- |
| 1 | C | 0.2748530 | -0.0656150 | 0.1598010 |
| 2 | C | 0.3165960 | -0.1042480 | 1.5558650 |
| 3 | C | 1.4641840 | -0.0353060 | -0.5609780 |
| 4 | C | 1.5402840 | -0.0988450 | 2.2238020 |
| 5 | C | 2.7089500 | -0.0418750 | 0.0915840 |
| 6 | C | 2.7454130 | -0.0563340 | 1.5057230 |
| 7 | C | 3.9716790 | -0.0251340 | -0.6145170 |
| 8 | N | 5.1674190 | 0.0485750 | 0.1477630 |
| 9 | C | 5.2319350 | 0.0140490 | 1.5442790 |
| 10 | C | 4.3299140 | -0.0354920 | -1.9275130 |
| 11 | N | 5.7319060 | 0.0327370 | -1.9781330 |
| 12 | C | 6.2893560 | 0.1031540 | -0.7124460 |
| 13 | C | 3.5171590 | -0.1332960 | -3.1570320 |
| 14 | C | 3.3381680 | 0.9859660 | -3.9862000 |
| 15 | C | 2.9271750 | -1.3569260 | -3.5137570 |
| 16 | C | 2.5744590 | 0.8809230 | -5.1511510 |
| 17 | C | 2.1604670 | -1.4569900 | -4.6768090 |
| 18 | C | 1.9836770 | -0.3384450 | -5.4977650 |
| 19 | O | 7.4782940 | 0.1827480 | -0.4447730 |
| 20 | O | 6.2865600 | 0.0255930 | 2.1564860 |
| 21 | H | -0.6754680 | -0.0615790 | -0.3640680 |
| 22 | H | -0.6019360 | -0.1384570 | 2.1332070 |
| 23 | H | 1.4360390 | -0.0048670 | -1.6427810 |
| 24 | H | 1.5381690 | -0.1314870 | 3.3052510 |
| 25 | H | 3.7947670 | 1.9302430 | -3.7087090 |
| 26 | H | 3.0668470 | -2.2215570 | -2.8717310 |
| 27 | H | 2.4366050 | 1.7528110 | -5.7838070 |
| 28 | H | 1.7047180 | -2.4063300 | -4.9419670 |
| 29 | H | 1.3874950 | -0.4168310 | -6.4021310 |
| 30 | C | 6.7278740 | 1.5324100 | -3.6659860 |
| 31 | O | 6.1866140 | 2.5117970 | -3.1955510 |
| 32 | O | 7.5571770 | 1.5444590 | -4.7175150 |
| 33 | C | 7.8299260 | 2.8424950 | -5.3225840 |
| 34 | H | 8.2402820 | 3.4998120 | -4.5507210 |
| 35 | H | 6.8812070 | 3.2665640 | -5.6650450 |
| 36 | C | 8.8025050 | 2.6133270 | -6.4614850 |
| 37 | H | 9.0325500 | 3.5694700 | -6.9422710 |
| 38 | H | 9.7363460 | 2.1774080 | -6.0946300 |
| 39 | H | 8.3746100 | 1.9425830 | -7.2125520 |
| 40 | C | 6.5527740 | 0.1027420 | -3.1590080 |
| 41 | H | 6.1148530 | -0.5024930 | -3.9571510 |
| 42 | H | 7.5382100 | -0.3055020 | -2.9212200 |
| 43 | N | 3.9915340 | -0.0230680 | 2.1778350 |
| 44 | C | 4.0312540 | -0.1042570 | 3.6279270 |
| 45 | C | 3.7281900 | -1.5096460 | 4.1375050 |
| 46 | H | 3.3515210 | 0.6201540 | 4.0815350 |
| 47 | H | 5.0426060 | 0.1499810 | 3.9500880 |
| 48 | O | 3.7418860 | -2.5150900 | 3.4592600 |
| 49 | O | 3.4699160 | -1.4746500 | 5.4541780 |
| 50 | C | 3.2348830 | -2.7558990 | 6.1082240 |
| 51 | H | 4.1173210 | -3.3844830 | 5.9570000 |
| 52 | H | 2.3833460 | -3.2390680 | 5.6200320 |
| 53 | C | 2.9743010 | -2.4726380 | 7.5737640 |
| 54 | H | 2.7943170 | -3.4149030 | 8.1010740 |
| 55 | H | 3.8341640 | -1.9775580 | 8.0345650 |
| 56 | H | 2.0948710 | -1.8336680 | 7.6983640 |

**Tab. 26S.** Atom coordinates of conformer H-F’.

| No | Symbol | X | Y | Z |
| --- | --- | --- | --- | --- |
| 1 | C | 0.2059810 | 0.0642010 | 0.0879170 |
| 2 | C | 0.2232640 | 0.1017180 | 1.4845350 |
| 3 | C | 1.4078140 | 0.0365570 | -0.6119150 |
| 4 | C | 1.4350300 | 0.0977050 | 2.1738910 |
| 5 | C | 2.6408870 | 0.0445300 | 0.0624110 |
| 6 | C | 2.6526600 | 0.0576930 | 1.4769990 |
| 7 | C | 3.9157560 | 0.0313530 | -0.6215900 |
| 8 | N | 5.0984010 | -0.0369500 | 0.1613800 |
| 9 | C | 5.1383150 | -0.0071810 | 1.5589510 |
| 10 | C | 4.2966320 | 0.0428100 | -1.9281700 |
| 11 | N | 5.6995510 | -0.0185740 | -1.9543930 |
| 12 | C | 6.2353070 | -0.0859090 | -0.6792470 |
| 13 | C | 3.5054380 | 0.1361370 | -3.1720740 |
| 14 | C | 2.9205000 | 1.3579170 | -3.5431450 |
| 15 | C | 3.3434370 | -0.9857590 | -4.0011720 |
| 16 | C | 2.1756550 | 1.4536650 | -4.7207160 |
| 17 | C | 2.6015430 | -0.8850330 | -5.1805160 |
| 18 | C | 2.0159260 | 0.3325910 | -5.5416930 |
| 19 | O | 7.4199160 | -0.1589630 | -0.3912220 |
| 20 | O | 6.1820040 | -0.0190200 | 2.1896240 |
| 21 | H | -0.7350240 | 0.0591810 | -0.4524940 |
| 22 | H | -0.7053090 | 0.1340710 | 2.0456790 |
| 23 | H | 1.3988370 | 0.0073260 | -1.6940780 |
| 24 | H | 1.4137790 | 0.1296050 | 3.2552150 |
| 25 | H | 3.0470350 | 2.2245680 | -2.9011050 |
| 26 | H | 3.7962520 | -1.9285440 | -3.7126030 |
| 27 | H | 1.7236870 | 2.4016110 | -4.9970870 |
| 28 | H | 2.4766770 | -1.7588640 | -5.8131840 |
| 29 | H | 1.4368300 | 0.4076270 | -6.4573750 |
| 30 | C | 6.7299890 | -1.5180390 | -3.6211900 |
| 31 | O | 6.1846900 | -2.4982490 | -3.1571760 |
| 32 | O | 7.5770010 | -1.5297650 | -4.6585080 |
| 33 | C | 7.8653970 | -2.8285280 | -5.2547110 |
| 34 | H | 6.9243370 | -3.2579960 | -5.6112330 |
| 35 | H | 8.2660060 | -3.4814470 | -4.4740360 |
| 36 | C | 8.8555330 | -2.5986920 | -6.3782560 |
| 37 | H | 9.0977250 | -3.5553740 | -6.8519550 |
| 38 | H | 8.4370000 | -1.9323930 | -7.1384910 |
| 39 | H | 9.7812400 | -2.1573310 | -5.9975650 |
| 40 | C | 6.5410580 | -0.0878420 | -3.1207160 |
| 41 | H | 7.5207170 | 0.3243190 | -2.8664410 |
| 42 | H | 6.1151720 | 0.5143660 | -3.9275890 |
| 43 | N | 3.8868770 | 0.0253560 | 2.1707280 |
| 44 | C | 3.9008140 | 0.1024150 | 3.6214980 |
| 45 | C | 3.5859510 | 1.5058220 | 4.1294240 |
| 46 | H | 4.9067350 | -0.1508560 | 3.9609820 |
| 47 | H | 3.2143630 | -0.6245310 | 4.0607010 |
| 48 | O | 3.6108800 | 2.5132990 | 3.4545250 |
| 49 | O | 3.3027300 | 1.4665690 | 5.4408260 |
| 50 | C | 3.0525550 | 2.7454850 | 6.0938270 |
| 51 | H | 2.2105430 | 3.2290430 | 5.5897650 |
| 52 | H | 3.9370230 | 3.3757310 | 5.9626600 |
| 53 | C | 2.7623050 | 2.4574420 | 7.5528320 |
| 54 | H | 2.5701750 | 3.3979090 | 8.0790770 |
| 55 | H | 1.8813710 | 1.8169520 | 7.6574350 |
| 56 | H | 3.6131830 | 1.9621690 | 8.0298190 |
